# Supplementary material for: Workforce predictive risk modelling: development of a model to identify general practices at risk of a supply−demand imbalance
Source: BMJ Open. 2020 Jan 23;10(1):e027934. doi: 10.1136/bmjopen-2018-027934 (PMC7044996; doi:10.1136/bmjopen-2018-027934)
Supplement: Supplementary data [file bmjopen-2018-027934supp001.pdf]

Appendix 1 - Data sources and preparation

Except where specified, national data for England were obtained and processed. A summary of data sources and data flow used in the modelling process is presented in Appendix 2.

GP Patient Survey

The General Practice Patient Survey (GPPS) is a national postal survey of patients' experience of primary care in England. Patients from practices that are known from prior surveys to have low response rates are oversampled. Full details of the sampling strategy are published elsewhere.[1] We used data from the 2011/12 and 2015/16 surveys. The contents of the survey have remained largely consistent over this time period. Response rates were 38% in 2011/12 (1,037,946 responses) and 39% in 2015/16 (836,312 responses).

We made use of three items from the GPPS reflecting access, continuity of care and overall experience. For patient's experience of access the following question was used:

- *"Last time you wanted to see or speak to a GP or nurse from your GP surgery:" "Were you able to get an appointment to see or speak to someone?"*

Responses of "Yes" and "Yes, but I had to call back closer to or on the day I wanted the appointment" were coded as a positive response and responses of "No" were coded as a negative response. Responses of "Can't remember" were treated as uninformative and excluded from the analysis.

The item on ability to see a preferred doctor is taken as a proxy measure for continuity of care:

- *"How often do you see or speak to the GP you prefer?"*

Responses of "Always or almost always" and "A lot of the time" were coded as a positive response and responses of "Some of the time" and "Never or almost never" were coded as a negative response. Responses of "Not tried at this GP surgery" were treated as uninformative and excluded from the analysis.

Finally, an item capturing data on the patient's overall experience of care is included:

- *"Overall, how would you describe your experience of your GP surgery?"*

Responses of "Very good" and "Fairly good" were coded as a positive response and responses of "Neither good nor poor", "Fairly poor" and "Very poor" were coded as a negative response. There were no uninformative options for this question.

Due to certain patient groups tending to give more positive responses in patient surveys, case-mix adjusted practice scores were created. This was achieved using mixed effects logistic regression adjusting for patient age, gender, and ethnicity, presence of a long-term condition, and deprivation

(using the Index of Multiple Deprivation [IMD], an area based measure assigned according to the patient's residential postcode[2 3]) and a random intercept for practice. The case-mix adjustment provides scores for individual practices based on a standardised mix of patients. The case-mix adjusted scores were used in the form of log-odds ratios relative to the average practice nationally.

Workforce

Workforce data were obtained from NHS Digital and related to the GP Census data taken as at 30 September 2012, 2013 and 2016.[4-6] Each dataset gave the headcount of GPs in 5-year age-bands for each practice. The 2012 dataset contained total GP headcount by gender as did the 2016 dataset. In the 2016 dataset additional detail of GP FTE by gender was provided. Both datasets contained total GP FTE as well as GP FTE broken down by GP role. We also extracted the total nurse FTE from the 2016 dataset. As nurse FTE data were not available in 2012, the relevant data were extracted from the 2013 dataset in its place. From these data two further variables were derived: the ratio of nurse FTE to GP FTE; the ratio "other" category FTE to total GP FTE (where "other" is assumed to mostly be Locum GPs given that GP registrars, salaried GPs, and those on retainer schemes are captured in other categories). These data were also used in the derivation of workload and the predicted remaining future workforce.

Practices with less than 0.5 FTE GP (38 out of 7,484 practices in 2012 data and 41 out of 6,709 practices in 2016 data) were excluded from all analyses on the basis that such a low staff record indicated either that these were unusual practices or that the workforce data were in error. In the former case such unusual practices are not the focus of this work and in the latter case, erroneous inferences may have been made if they had been included.

GP quitting intentions

To predict remaining future workforce we utilised self-reported GP intentions to cease practice collected through a survey which formed part of the ReGROUP project and has been reported.[7] Briefly, a questionnaire was administered to all active GPs in South West England in April-June 2016, enquiring about their intentions to cease/interrupt practice in the next 2 and 5 years (3370 questionnaires sent, 2248 returned, response rate 67%). We combined responses to two questions:

- *"How likely is it that you will permanently leave direct patient care within the next 5 years?"*
- *"How likely is it that you will take a career break (or another career break) within the next 5 years?"*

Each question had response options of "Very Likely", "Likely", "Unlikely" and "Very unlikely". Where GPs gave different response options for these two questions, the response with the highest likelihood

of cessation or interruption was taken. This reflects the most likely chance of impact to future GP workforce in the next 5 years. We also used respondents' answers to the question:

- *"In your current/most recent direct patient role, how many sessions do/did you work in a typical week?"*

Free text responses to this question provided data from which an estimate of each responder's current FTE work commitment could be calculated. Working eight sessions per week was taken as 1 FTE, consistent with the approach used in the GP census.[6] When more than eight sessions was given as a response the FTE was capped at 1. If more than 24 sessions was given as a response it was assumed the question had been answered incorrectly and the data were treated as missing. Data for all GPs surveyed on age, gender and affiliated practice were obtained from the Performers List.

Practice rurality and deprivation

Practice rurality was contained within the GPPS 2016 dataset and was based on an Office for National Statistics (ONS) categorisation of the postcode of the practice. We used a rural/urban version of this categorisation. Practice deprivation score was obtained from Public Health England and was based on the 2015 IMD. Individual patient IMD is based on each patient's residential postcode, and the practice score is the mean of individual patient scores using all patients registered at the practice.[8]

Practice registered population

Data on the registered populations for each general practice were obtained from NHS Digital for each quarter from April 2014 to April 2016 (9 datasets); as well as April 2012. These datasets provided the count of patients in each gender by 5-year age-band (with the highest age-band being 95 and over). We aggregated the top three age-bands resulting in a top age-band category of 80+ years.

The April 2012 and April 2016 datasets were used to calculate list sizes weighted for the demographics of the populations and adjusted for deprivation. The reason for weighting for patient demographics is that certain types of patients (older, female and very young) place a higher demand on practices than others. The adjustment for deprivation acknowledges that deprived populations have higher health needs than less deprived populations with a similar demographic profile. To calculate weighted list sizes the practice populations were weighted according to the average time spent consulting with patients in 14 age by gender groups in 2013/14 according to a recent study based on routine patient records from 674 practices.[9] Weighted lists sizes ($\boldsymbol{P_W}$) were then normalised so the total population across the country remained unchanged. These weighted list sizes are taken as a measure of workload on the basis that they represent a measure of the expected time spent consulting. This assumes that, on average, patients in the same demographic group require the same amount of consultation time. Because age and gender do not capture the health status of the population the weighted list sizes

were then adjusted for deprivation (IMD decile, $\boldsymbol{IMD_i}$, taking a value between 1 and 10, based on all practices in England) assigning a 10% weighting to a deprived population. The adjusted weighted population will thus be given by

$$\boldsymbol{P_{AW} = 0.9P_W + 0.1\left(\frac{P_W IMD_i}{\sum P_W IMD_i}\sum P_W\right)} \qquad 1$$

This approach is intended to mirror that used in the current resource allocation to CCGs. However, the CCG allocations do not use deprivation, but rather make use of a measure of premature mortality (the <75 standardised mortality ratio, which is the ratio of mortality in under 75 year olds to that expected given the age and sex composition of the CCG population). We chose to use deprivation here as standardised mortality ratios are not published for individual practices.

The adjusted weighted list sizes were divided by the total GP FTE to obtain a measure of workload per GP. Initial inspection of the workload figures showed that the distribution contained some infeasibly large and small values. These may have arisen from errors in either the workforce or practice population data. Unfortunately, there was no clear separation between typical values and those that were infeasible. A pragmatic approach was taken whereby practices in the top and bottom 2.5% of the distribution were excluded from all further analysis. This exclusion took place following the removal of practices with less than 0.5 GP FTE.

The data from April 2014 to April 2016 were used in the prediction of future practice populations along with the subnational population projections described below.

Subnational population projections

We made use of ONS subnational population projections at the level of CCGs (used to inform local planning of healthcare and other public services[10 1110 11]) in the prediction of future practice populations (see below). The subnational ONS projections are demographic, trend-based projections that indicate the 'likely levels of future population' and are currently produced every 2 years; they present projections for every year for the next 25 years from the base year.[12] The underlying data sources that inform the calculations include: national population projections; registration of births and deaths (General Registrar Office); armed forces data (MOD); data extracts from the Patient Register Data System (NHS); student location data (Higher Education Statistics Agency [HESA]); and data on asylum seekers (Home Office). Adjustments were then made to the datasets for factors such as assumed fertility and mortality rates, internal and international migration. However, the projections do not account for local development aims and policies, economic factors, and indeed any international factors that are likely to affect the UK population.[10] We extracted projected populations

for 2021 for the eight CCGs within the scope of the ReGROUP project: NHS Bath and North East Somerset CCG; NHS Kernow CCG; NHS North, East, West Devon CCG; NHS South Devon and Torbay CCG; NHS Bristol CCG; NHS North Somerset CCG; NHS Somerset CCG; NHS South Gloucestershire CCG. Projections are made in 5-year age-bands for each gender. As with practice population data the upper age groups were combined to form an 80+ age-band.

Projecting future workload

Our projections of future practice workloads were based on the number of patients registered at each of the 423 GP practices in South West England, in 5-year age bands, split by gender combined with subnational population projections from the ONS as described above. The approach comprises the following five steps.

1. Assess congruency of ONS predictions with list size
   ONS subnational population projections were compared with GP list size data aggregated to CCG level for 2014, 2015 and 2016. This provided an assessment of the degree to which ONS predications reflect the actual GP list size data in those years. This difference between the two data sources is most likely due to "list inflation", caused by patients that have not been removed from the list following death, dual registrations for patients when moving homes or by a registered patient's failure to complete the national census.[9] Given that the average consultation times used to weight the populations (described above) are based on registered patients, we did not consider it appropriate to resize practice list sizes to reflect the identified difference.
2. Calculate the proportion of CCG population registered at each GP practice
   For each practice, and for each age-band by gender stratum, we identified the number of patients registered with the practice and the expected number of patients within a CCG for nine time-points between April 2014 to April 2016. This allowed us to derive the proportion of the total CCG population by gender/age-interval registered at each practice. If the number of practices in a CCG is declining over time we might expect the proportion of the CCG population to be rising at the remaining practices.
3. Quantify trends in the proportion of the CCG population registered at each general practice
   The data from step 2 were used as the outcome variable in a logistic regression model that included a linear term for time as well as a categorical variable for quarter to quantify trends. A separate regression model was used for each practice by age-band by gender strata.
4. Determine projected count of patients
   We used the resultant regression equation to predict the proportion of CCG patients by practice/gender/age-interval for five years beyond the final data point. Multiplying this

proportion by the ONS predicted population for the same time point gives a projected count of patients.

5. Project adjusted list size
   The projected populations were used to create a projected adjusted weighted list size using the same algorithm used above for observed populations.

Predicting remaining future workforce

When predicting future workforce (supply) we concentrated on predicting what fraction of the existing workforce will remain available to the practice in 5 years' time. We did this in two principal ways: i) based on the age and gender of GPs at the practice; ii) based on responses to the ReGROUP survey of GP quitting intentions. Predictions are made based on 2012 data and 2016 data (with the survey only being available for the 2016 data).

Approach 1 – Using the age and gender profile of GPs at each practice.

Previous work has identified the probability that GPs of a given single year of age and gender will remain in the workforce 12 months later.[13] By multiplying these probabilities over five consecutive single year age bands we obtain the probability that GPs of a given single year of age and gender will remain in the workforce in 5 years' time. As the routinely available GP census data (p.**Error! Bookmark not defined.**) is only available in five-year age-bands, we take the mean of these 5-year probabilities over the 5-year age-bands used in the GP census data. Unfortunately, the GP census data published at practice level gives data by either age or gender, but not both. Furthermore, data by age is only given in terms of headcount, as is data by gender in 2012 (data by gender is given in terms of headcount and FTE in 2016). Thus we adopted the following procedure to estimate remaining workforce.

1. Using the probabilities described above, identify the probability that each GP in the practice will remain in patient care in 5 years' time based on their age-band assuming they are male.
2. Calculate the mean of these probabilities over all GPs in the practice.
3. Repeat steps 1 and 2 assuming they are female.
4. Take a weighted average of the probabilities obtained in steps 2 and 3 weighted by the FTE of male and female GPs in the practice (in 2012 data headcount by gender is used instead).

The resulting probabilities can then be interpreted as the proportion of GP FTE which is expected, on average, to remain at the practice in 5 years' time.

Approach 2 – Using the ReGROUP survey responses.

An alternative approach used in the forecasting utilised the results of the ReGROUP survey where all GPs in South West England were asked about their future career intentions. For GPs who responded to the survey (67%) we used both stated career intentions, stated FTE (as described above), and information on age and gender. For non-responders we simply used age and gender information (provided within the Performers List). To incorporate the survey responses we made use of odds ratios estimated from a previous study which linked stated quit intentions to working status 5 years later and adjusted for age and gender.[14] Odds ratios for their 5-point scale are mapped to our 4-point scale by ignoring the middle (neutral) option.

1. It proved difficult to map the ReGROUP survey responses to the NHS GP census data (due to inconsistent age, gender and FTE information between the two data sources). Therefore, in this methodology, the GP census data are only used in the estimation of FTE of survey non-responders based on difference between the total GP FTE (GP census data) and the total FTE stated by responders linked to each practice within the Performers List. This was done using the following method. We calculated the difference between the total GP FTE given in the GP census data and the stated total GP FTE of responders to the survey linked through the Performers List to each practice in the study. The assumed FTE of non-responders was this difference divided by the number of non-responders linked to the practice. Where this difference was greater than the number of FTEs, the non-responders were assigned an FTE of 1. Where this difference was negative, non-responders were assigned an FTE of 0.

2. We then calculated probabilities of remaining in patient care for the forthcoming 5 years. For the survey non-responders, we assigned a probability of remaining in patient care using the same method as in approach 1 but based on the individual GP's gender and current year of age taken from the Performers List (rather than the GP census). For responders, we similarly assigned a probability of remaining in patient care based on the individual GP's age and gender and then adjusted that probability using the following odds ratios (Calculated from Hann et al. [14] but changing the baseline to the neutral category) "Very likely" 1.94, "Likely" 1.3, "Unlikely" 0.70, and "Very unlikely" 0.43.

3. For each practice, we then took the weighted average of the probabilities obtained in step 2 (over GPs associated with a practice, weighted according to their FTE.

The resulting probabilities can then be interpreted as the proportion of GP FTE that would be expected, on average, to remain at the practice in 5 years' time.

References

1. NHS England. GP Patient Survey: NHS England; [Available from: https://gp-patient.co.uk/surveys-and-reports accessed 05/03/2018.
2. Paddison C, Elliott M, Parker R, et al. Should measures of patient experience in primary care be adjusted for case mix? Evidence from the English General Practice Patient Survey. *BMJ Qual Saf* 2012
3. Abel GA, Saunders CL, Lyratzopoulos G. Cancer patient experience, hospital performance and case mix: evidence from England. *Future Oncol* 2014;10(9):1589-98. doi: 10.2217/fon.13.266
4. NHS Digital. General and personal medical services, England 2002-2012 as at 30 September 2012 [Available from: http://content.digital.nhs.uk/searchcatalogue?productid=10382&topics=1%2fWorkforce%2fStaff+numbers&kwd=GP+staff&geo=England&sort=Relevance&size=10&page=2#top accessed 31/07/2017 2017.
5. NHS Digital. General and personal medical services, England 2003-2013 as at 30 September 2013 [
6. NHS Digital. General and personal medical services, England September 2015-March 2016, Provisional Experimental statistics 2016 [Available from: http://content.digital.nhs.uk/catalogue/PUB21772 accessed 31/07/2017 2017.
7. Fletcher E, Abel GA, Anderson R, et al. Quitting patient care and career break intentions among general practitioners in South West England: findings of a census survey of general practitioners. *BMJ Open* 2017;7(4):11. doi: 10.1136/bmjopen-2017-015853
8. Public Health England. National General Practice Profile 2013 [Available from: http://fingertips.phe.org.uk/profile/general-practice accessed August 2104 2014.
9. Hobbs FR, Bankhead C, Mukhtar T, et al. Clinical workload in UK primary care: a retrospective analysis of 100 million consultations in England, 2007–14. *The Lancet* 2016;387(10035):2323-30.
10. Office for National Statistics (ONS). Subnational population projections for England: 2014-based projections 2014 [Available from: https://www.ons.gov.uk/peoplepopulationandcommunity/populationandmigration/populationprojections/bulletins/subnationalpopulationprojectionsforengland/2014basedprojections accessed 31/07/2014 2017.
11. Office for National Statistics. Subnational population projections for England: 2014-based projections 2014 [Available from: https://www.ons.gov.uk/peoplepopulationandcommunity/populationandmigration/populationprojections/bulletins/subnationalpopulationprojectionsforengland/2014basedprojections accessed 31/07/2014 2017.
12. Office for National Statistics (ONS). Quality and Methodology Information - Subnational Population Projection. 2016

13. Centre for Workforce Intelligence. In-depth review of the general practitioner workforce: final report. London, 2014.
14. Hann M, Reeves D, Sibbald B. Relationships between job satisfaction, intentions to leave family practice and actually leaving among family physicians in England. *European journal of public health* 2011;21(4):499-503. doi: 10.1093/eurpub/ckq005 [published Online First: 2010/02/10]
