# Supplementary material for: Workforce predictive risk modelling: development of a model to identify general practices at risk of a supply−demand imbalance
Source: BMJ Open. 2020 Jan 23;10(1):e027934. doi: 10.1136/bmjopen-2018-027934 (PMC7044996; doi:10.1136/bmjopen-2018-027934)

Appendix 2a – Data Flow Main development model

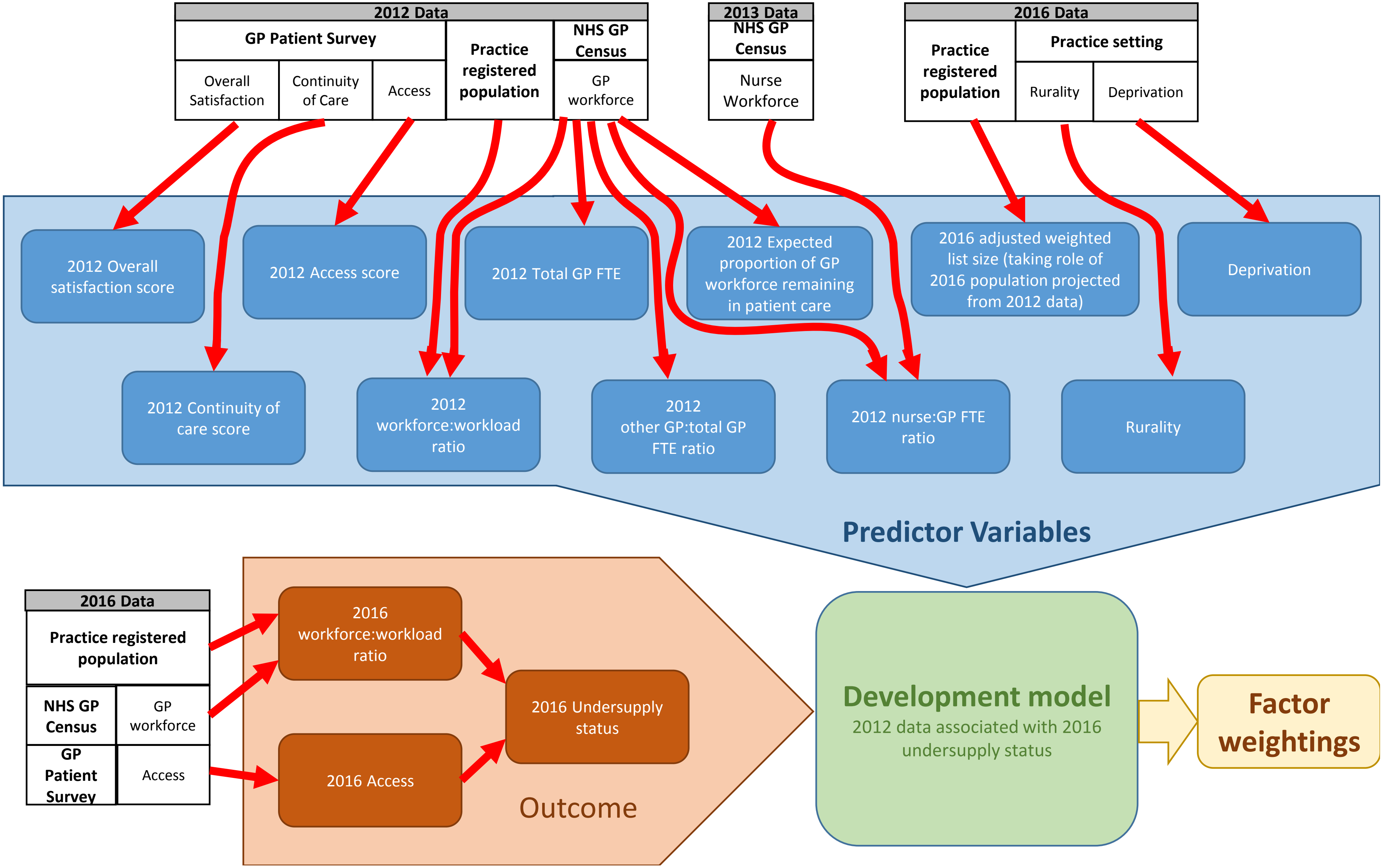

Appendix 2b – Data Flow Main prediction model

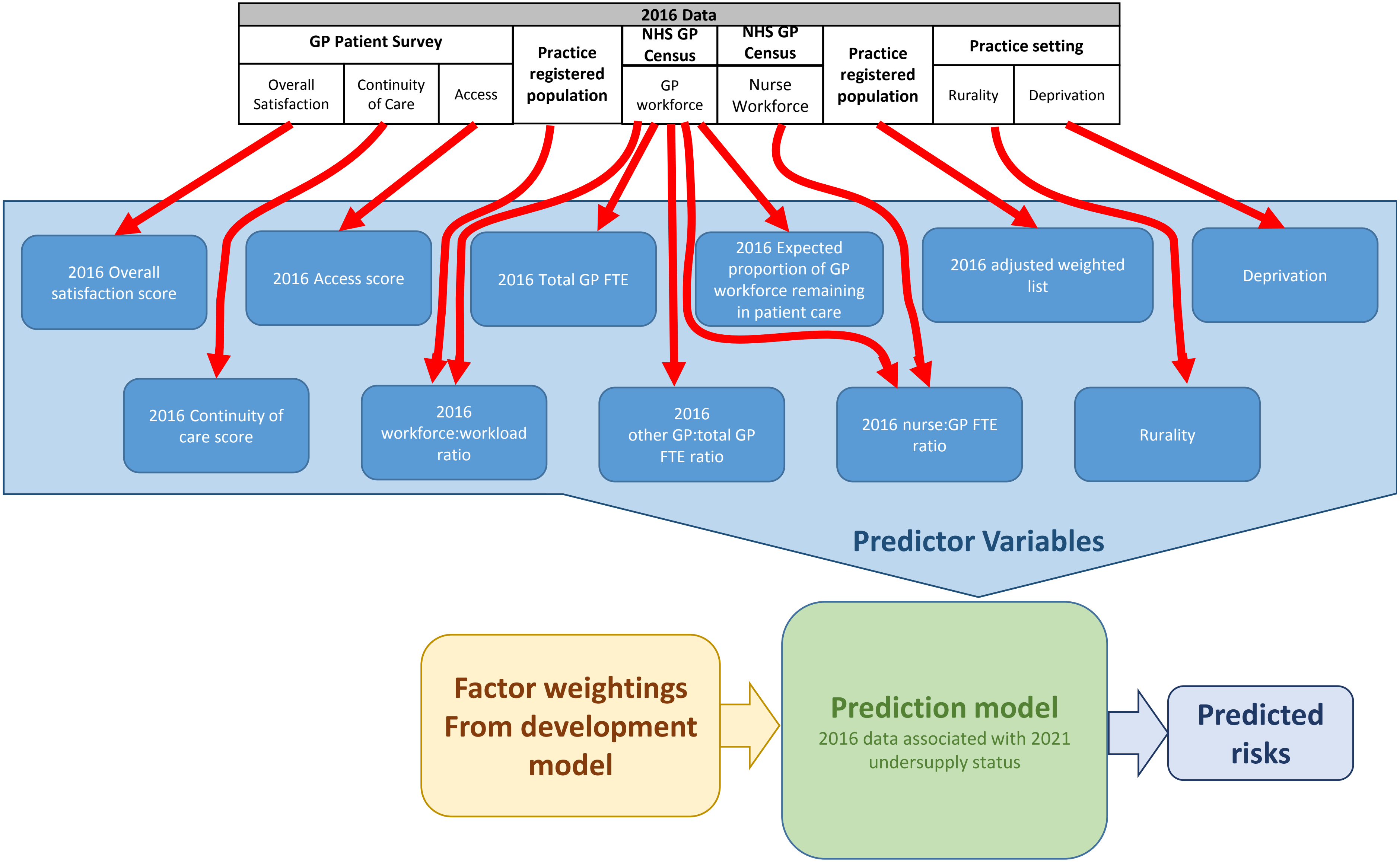

Appendix 2c – Data Flow Simpler development model

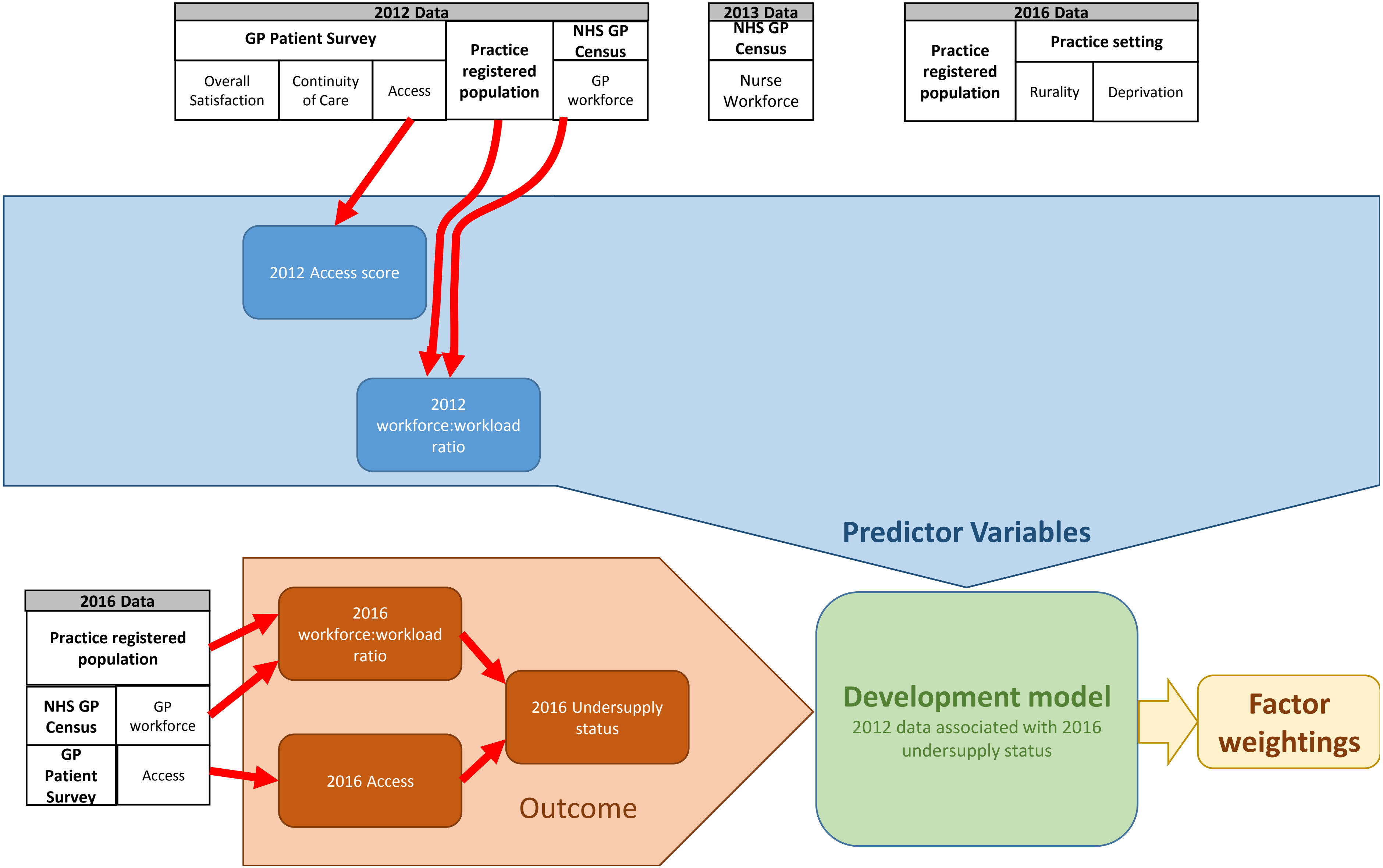

Appendix 2d – Data Flow Simpler prediction model

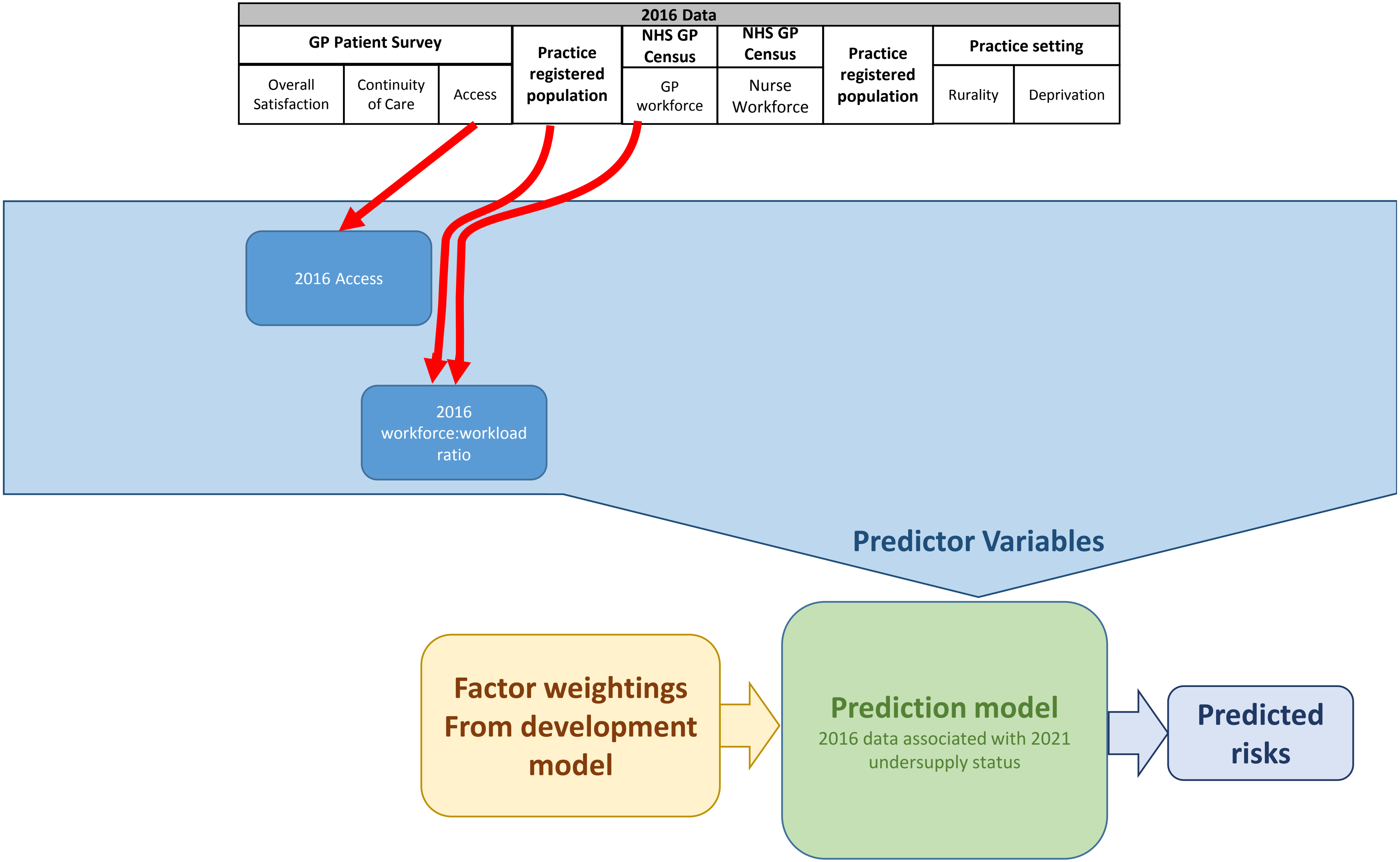

Supplement: Supplementary data [file bmjopen-2018-027934supp002.pdf]
