# Supplementary material for: Workforce predictive risk modelling: development of a model to identify general practices at risk of a supply−demand imbalance
Source: BMJ Open. 2020 Jan 23;10(1):e027934. doi: 10.1136/bmjopen-2018-027934 (PMC7044996; doi:10.1136/bmjopen-2018-027934)

Appendix 3 – Calibration curve

In order to assess the calibration of the model we used predicted probabilities of being in undersupply from the development model (i.e. 2012 covariates and 2016 outcome) and split the practices into 10 groups according to deciles of this predicted probability. We then calculated the mean predicted probability in each group as well as the percentage of practices in undersupply in 2016. The relationship between these two quantities is shown in the figure below.

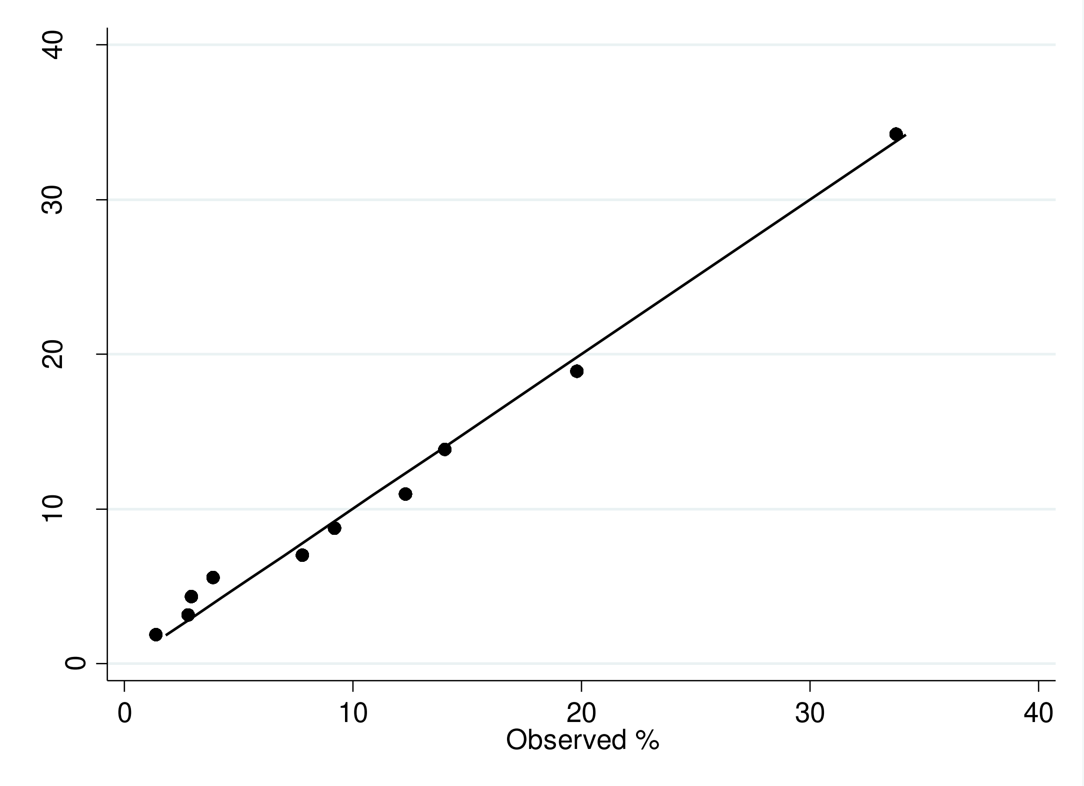

Supplement: Supplementary data [file bmjopen-2018-027934supp003.pdf]
