# Supplementary material for: Workforce predictive risk modelling: development of a model to identify general practices at risk of a supply−demand imbalance
Source: BMJ Open. 2020 Jan 23;10(1):e027934. doi: 10.1136/bmjopen-2018-027934 (PMC7044996; doi:10.1136/bmjopen-2018-027934)
Supplement: Author's manuscript [file bmjopen-2018-027934.draft_revisions.pdf]

BMJ Open is committed to open peer review. As part of this commitment we make the peer review history of every article we publish publicly available.

When an article is published we post the peer reviewers' comments and the authors' responses online. We also post the versions of the paper that were used during peer review. These are the versions that the peer review comments apply to.

The versions of the paper that follow are the versions that were submitted during the peer review process. They are not the versions of record or the final published versions. They should not be cited or distributed as the published version of this manuscript.

BMJ Open is an open access journal and the full, final, typeset and author-corrected version of record of the manuscript is available on our site with no access controls, subscription charges or pay-per-view fees (http://bmjopen.bmj.com).

If you have any questions on BMJ Open's open peer review process please email info.bmjopen@bmj.com

BMJ Open

Workforce predictive risk modelling – development of a model to identify general practices at risk of a supply-demand imbalance.

Journal:	*BMJ Open*
Manuscript ID	bmjopen-2018-027934
Article Type:	Research
Date Submitted by the Author:	23-Nov-2018
Complete List of Authors:	Abel, Gary; University of Exeter, University of Exeter Medical School (Primary Care)<br>Gomez-Cano, Mayam; University of Exeter Medical School<br>Mustafee , Navonil ; University of Exeter Business School<br>Smart, Andi; University of Exeter Business School<br>Fletcher, Emily; University of Exeter Medical School, Primary Care Research Group<br>Salisbury, Chris; University of Bristol, Academic Unit of Primary Health Care<br>Chilvers, Rupa; Tangerine Bee<br>Dean, Sarah; PenCLAHRC University of Exeter Medical School,<br>Richards, Suzanne; University of Leeds, Academic Unit of Primary Care; University of Leeds, Academic Unit of Primary Care<br>Warren, F; University of Exeter,<br>Campbell, John; University of Exeter, Primary Care;
Keywords:	PRIMARY CARE, Workforce, Supply-Demand, Organisation of health services < HEALTH SERVICES ADMINISTRATION & MANAGEMENT

SCHOLARONE™
Manuscripts

Workforce predictive risk modelling – development of a model to identify general practices at risk of a supply–demand imbalance.

Gary Abel *Senior Lecturer*[1]*, Mayam Gomez-Cano *Research Associate*[1], Navonil *Mustafee Associate Professor*[2], Andi Smart *Professor of Operations & Process Management*[1], Emily Fletcher *Research Fellow*[1], Chris Salisbury *Professor of Primary Health Care*[3], Rupa Chilvers *Director of Research and Knowledge Transfer4*, Sarah *Dean Professor in Psychology Applied to Rehabilitation and Health*[1], Suzanne Richards *Professor of Primary Care Research*[,5], Fiona Warren *Senior Lecturer*[1], John Campbell *professor of general practice and primary care*[1]

[1] University of Exeter Medical School, Exeter, UK

[2] University of Exeter Business School, Exeter, UK

[3] Centre for Academic Primary Care, NIHR School for Primary Care Research, School of Social and Community Medicine, University of Bristol, Bristol, UK

[4] Tangerine Bee, Exeter, UK

[5] Academic Unit of Primary Care, Leeds Institute of Health Sciences, UK

*Corresponding author:

Dr Gary Abel
Primary Care Research Group
University of Exeter Medical School
Smeall Building, St Luke's Campus
Magdalen Road
Exeter
EX1 2LU
g.a.abel@exeter.ac.uk

Word count (excludes title page, abstract, references, figures, tables): 4745

Key Words. Primary Care, workforce, supply-demand, organisation of care

For peer review only

Objective. To develop a risk prediction model identifying general practices at risk of workforce supply–demand imbalance

Design. Secondary analysis of routine data on general practice workforce, patient experience and registered populations (2012 to 2016), combined with a census of general practitioners' career intentions (2016).

Setting/Participants. A hybrid approach was used to develop a model to predict workforce supply–demand imbalance based on practice factors using historical data (2012–2016) on all general practices in England (with over 1000 registered patients n=6,398). The model was applied to current data (2016) to explore future risk for practices in South-West England (n=368).

Primary outcome measure. The primary outcome was a practice being in a state of workforce supply–demand imbalance operationally defined as being in the lowest third nationally of access scores according to the GP Patient Survey and the highest third nationally according to list size per full time equivalent GP (weighted to the demographic distribution of registered patients and adjusted for deprivation)

Results. Based on historic data, the predictive model had fair to good discriminatory ability to predict which practices faced supply–demand imbalance (area under the ROC curve 0.759). Predictions using current data suggested that, on average, practices at highest risk of future supply–demand imbalance have larger patient lists, employ more nurses, serve more deprived and younger populations, and have considerably worse patient experience ratings. Incorporating findings from a survey of GPs career intentions made little difference to predictions of future supply–demand risk status when compared with expected future workforce projections based only on routinely-available data on GPs' gender and age.

Conclusions. It is possible to make reasonable predictions of an individual general practice's future risk of undersupply of general practitioner workforce with respect to its patient population. However, the predictions are inherently limited by the data available.

For peer review only

Strengths and limitations of this study

- This study makes use of freely available data from a range of sources to develop a predictive model of workforce supply-demand imbalance for general practices in England
- Historical data for all of England is used to develop factor weightings which are then applied to current data.
- The additional value of a census survey of career intentions of GPs in south-west England is explored comparing findings to predictions made on the basis of general practice workforce age and Gender alone.
- The predictive model is inherently limited by the data available, and in particular we note that routine data of a measure of a practices difficulty in recruiting staff were not available.

Data sharing statement. Most data used in this study are publicly available from referenced sources. Data from the GP Census survey can be made available on request from the corresponding author of the original publication at john.campbell@exeter.ac.uk.

Funding. The project was funded by the National Institute for Health Research, Health Service and Delivery Research programme (project 14/196/02). The views and opinions expressed herein are those of the authors and do not necessarily reflect those of the Health Service and Delivery Research programme, the National Institute for Health Research, the National Health Service, or the Department of Health.

Competing interests. All authors have completed the ICMJE uniform disclosure form at www.icmje.org/coi_disclosure.pdf (available on request from the corresponding author) and declare: grants from the National Institute for Health Research (Health Service and Delivery Research programme) during the conduct of the study. SD's position is partly supported by the National Institute for Health Research (NIHR) Collaboration for Leadership in Applied Health Research and Care South West Peninsula at the Royal Devon and Exeter NHS Foundation Trust. The views expressed are those of the author(s) and not necessarily those of the NHS, the NIHR or the Department of Health.

For peer review only

Introduction

Against a backdrop of 34,495 full time equivalent (FTE) General Practitioners in 2016, the NHS in England saw a reduction of 3.5% of the English general practitioner (GP) workforce (1193 FTE) in a single year.[1] This reduction has been seen in combination with rising demands of the patient population.[2] Such figures represent a crisis in respect of GP workforce capacity, with particular problems in retaining established GPs in direct patient care [3 4]. Similar problems in respect of family doctor recruitment and retention are evident in other western healthcare economies and jurisdictions [5 6], and many countries have explored what might constitute optimal skill mix amongst primary care health professionals over the last 40 years [7-9].

There is, however, a need for the rational deployment of GP workforce resource.[10] Various models exist to inform that deployment, with GP workload representing a key issue amongst individual GPs electing to quit patient care [3]. Gaining an understanding of GP workload pressures is also the basis of identifying any potential mismatch between the demand for general practice services, and the supply of GPs to meet that demand. In many countries, the general practice represents a key element in the delivery of primary care and acts as the basis for general practice workforce planning. For example, practices are the basis of reporting of patients' experience of primary care in England, captured using the General Practice Patient Survey [11].

The aim of this research was to develop a method to identify NHS general practices, in one region of England, which, may face supply–demand workforce imbalances within the next 5 years. Previous workforce modelling in the UK has focussed upon deriving insights from analyses at the regional or national (macro) level [12]. In contrast, this research focuses on undertaking predictive risk modelling at a practice (micro) level. Routine workforce modelling makes use of data on doctors' age and gender, and historical retirement patterns. Here we consider whether surveying GPs' career intentions adds value to such modelling.

Methods

Overview

The first step in developing a predictive model to identify general practices at risk of future supply–demand imbalance is to define what is meant by a supply–demand imbalance and to operationalise this with measurable quantities. Assessing the supply of GP workforce at any one general practice is reasonably straightforward, however assessing the demand of patients is complex as unmet demand is, by its nature, hard to quantify. Instead here we consider the expected workload given the demographics of the patient population served. The balance between supply and demand within this framework is then represented by the expected workload per practitioner. However, high workload alone may not be an issue. Practices with high workload may meet patient demand through innovative and efficient

For peer review only

systems of service delivery. High workload is considered to have a negative impact only when service delivery is impaired. For the purposes of this study we defined those practices with high workload per practitioner in combination with an inability to meet patient demand as being in a state of 'under-supply'. Here we use the term 'under-supply' to indicate a practice which has a high demand from patients for a given supply of doctors which appears to be having a detrimental impact on services [13]. In this study we used a measure of patient access as a proxy for the ability to meet patient demand, in the belief that access is an important measure, reflecting the ease with which patients might engage with the primary healthcare system [13 14].

Several data sources have been brought together in this work. Analyses were performed at general practice level, firstly, to identify practices which were currently in 'under-supply' and, secondly, to identify those which are likely to have such problems in future. A predictive risk model (to predict the risk of a practice being in a state of 'under-supply' within 5 years) was developed by assessing the associations between current (2016) 'under-supply' status and historical routinely collected data (where available) on GP workforce, practice characteristics (rurality, deprivation, population) patient experience scores from 2012. The model further incorporated projected future populations in each area and considered projected future GP workforce based on GPs stated career intentions (from a survey of GPs). The rationale for this approach was to obtain factor weightings informed by evidence developed on past data. This model was then used to identify practices and areas in South West England that are likely to experience a supply–demand imbalance ('under-supply') in the future.

Data sources

Except where specified, national data for England were obtained and processed. A summary of data sources is given below with full details given in Supplementary Online Material 1 along with a schematic illustrating the data flow used in the modelling process (Supplementary Online Material 2).

GP Patient Survey

The GPPS is a national postal survey of patients' experience of primary care in England distributed to around 2.8 million adult patients each year [11]. We used data from the 2011/12 and 2015/16 surveys, during which the contents of the survey remained largely consistent. Response rates were 38% in 2011/12 (1,037,946 responses) and 39% in 2015/16 (836,312 responses) with an average of around 125 respondents per practice. We used three GPPS items GPPS reflecting access, continuity of care, and overall experience.

Workforce

Workforce data at practice level were obtained from NHS Digital and related to GP Census data taken as at 30 September 2012, 2013 and 2016 [15-17]. Total GP FTE and GP FTE in the "other" category were extracted for 2012 and 2016 (where "other" is assumed to mostly be locum GPs given that registrars, salaried GPs, and those on retainer schemes, are captured

For peer review only

in specific categories). Total nurse FTE was extracted for 2013 and 2016 (nurse data were not available in 2012, so 2013 data were used in its place).

General practitioner Quitting intentions

Self-reported GP intentions to cease practice were collected through a census survey which has been reported elsewhere [18]. Briefly, a questionnaire was administered to all active GPs in South West England enquiring about their intentions to cease/interrupt practice within 2 and 5 years. We make use of responses to three questions:

- *"How likely is it that you will permanently leave direct patient care within the next 5 years?"*
- *"How likely is it that you will take a career break (or another career break) within the next 5 years?"*
- *"In your current/most recent direct patient role, how many sessions do/did you work in a typical week?"*

The first two questions had response options of "Very Likely", "Likely", "Unlikely" and "Very unlikely" whereas the latter had a free text response.

Practice rurality and deprivation

Practice rurality (rural/urban) based on an Office for National Statistics (ONS) categorisation of the postcode of the practice was obtained, as was a practice deprivation score based on the 2015 Index of Multiple Deprivation (IMD) [19].

Practice registered population

Data on the registered populations for each general practice were obtained for each quarter from April 2014 to April 2016 (9 datasets); as well as April 2012. These datasets provided the count of patients of each gender (male, female) by 5-year age-band strata.

Subnational population projections

We made use of the Office for National Statistics (ONS) subnational population projections developed to inform the local planning of healthcare and other public services for geographically defined populations served by Clinical Commissioning Groups (CCGs, organisations responsible for commissioning NHS services) [20]. These projections are demographic, trend-based projections that indicate the 'likely levels of future population' and are routinely produced every 2 years. We extracted projected populations for 2021 for the eight CCGs within South West England. Projections were made in 5-year age-bands for each gender.

Data preparation and variable creation

Brief details are given below with full details in Supplementary Online Material 1

Patient experience

Case-mix adjusted practice scores for patient experience were created following previous methodology [21 22] adjusting for patient age, gender, ethnicity, presence of a long-term

For peer review only

condition, and deprivation, using mixed effects logistic regression. The case-mix adjusted scores were based on dichotomous outcomes and used in the form of log-odds ratios relative to the average practice nationally.

Workforce

Practices with less than 0.5 full-time equivalent (FTE) GPs (38 out of 7,484 practices in 2012 data and 41 out of 6,709 practices in 2016 data) were excluded from all analyses on the basis that such a low staff record indicated either that these were unusual practices or that the workforce data were in error. In addition to total GP FTE, the ratio of nurse FTE to doctor FTE and the ratio of doctor FTE in the "other" category to total doctor FTE were calculated.

Workload

Weights were applied to patient list sizes in order to standardise for the age and gender composition of the practice population, accounting for the fact that GPs spend longer consulting with patients who are very young, are older, or are female [2]. Further adjustment was made for the deprivation of the practice population to reflect higher health needs. These adjusted weighted list sizes were divided by the total GP FTE to obtain a measure of workload per GP FTE. Initial inspection of the workload figures showed that the distribution contained some infeasibly large and small values. Practices in the top and bottom 2.5% of the distribution were excluded from all further analysis. This exclusion took place following the removal of practices with less than 0.5 GP FTE.

Expected remaining future workforce

We estimated the proportion of GP FTE that would be expected, on average, to remain at the practice in 5 years' time. We did this in two principal ways: (i) based on the age and gender of GPs at the practice and (ii) based on responses to survey of GP career intentions. The former was done for both 2012 and 2016 data and the latter only for 2016 data. The approaches are detailed in full in Supplementary Online Material 1.

Outcome definition

Ability to meet patient demand was quantified using the GPPS access measure, reflecting the ease with which patients might engage with the primary healthcare system. Workload to workforce ratio was quantified using the workload per GP FTE quantity described above. Practices that were simultaneously in the lowest third of GPPS access scores and the highest third of workload per GP FTE nationally were defined as being in 'under-supply' (i.e. demand exceeded supply).

Development of predictive risk model

Historical data were used to produce model coefficients which could then be applied to current data. Model development was based on national data in order to maximise statistical power. Data from 2012 were used to quantify independent associations between the considered factors (three GPPS scores, adjusted weighted list size per GP FTE, total GP FTE, the ratio of "other" GP FTE to total GP FTE, rurality setting, practice deprivation, ratio of

For peer review only

nurse FTE to doctor FTE, the expected proportion of GP FTE still in patient care in 2017) and supply–demand imbalance observed in 2016. We did not attempt to predict the 2016 practice populations using only data available in 2012 and instead included the observed 2016 practice populations as an additional explanatory variable due to a lack of data available for 3 years prior to 2012.

A logistic regression model was used with a binary outcome of a practice being in a state of under-supply. Practices were the unit of analysis. All variables considered were included and retained regardless of statistical significance. We recognised the need to account for the fact that GPs leaving patient care would be most likely to impact the supply–demand balance when recruitment of GPs is difficult. This was accounted for by including an interaction between the expected proportion of the GP workforce remaining in patient care after five years and the ratio of total nurse FTE to total doctor FTE based on NHS workforce data. The rationale for this decision is outlined in Supplementary Online Material 3.

The predictive value of our model was assessed using a ROC (Receiver Operating Characteristic) curve analysis of predicted probabilities for all practices in England based on the data used to build the model (i.e. 2012 data and 2016 supply–demand imbalance classifications). These were compared with a simpler model developed using only defining factors (GPPS access scores and adjusted weighted list size per FTE).

Future risk prediction

The coefficients from the historical model were applied to 2016 data to form our baseline risk predictions with a 5-year forward view for practices in South West England only. The reason for the restriction to those practices was that they were the only ones for which we had survey responses on future career intentions). Practices in the highest 25% of the predicted risk profile were flagged as “high risk” of future under-supply of GP workforce, those in the lowest 25% were flagged as being “low risk”, and those in between were flagged as being at “moderate risk”.

The usefulness of the career intention survey was examined by comparing the above prediction with an alternative prediction using the expected proportion of the GP workforce remaining in patient care in five years’ time based only on the routinely available age and gender profile of GPs in the practice.

In addition to baseline predictions, we explored a number of `stress testing’ scenarios. These scenarios can be considered as stress tests of the model to identify practices that might be more (or less) vulnerable to particular challenges. First, we explored the effect of increased difficulty in recruiting GPs, which we modelled as an increase in the coefficient for the expected proportion of GPs remaining in patient care (where an increased coefficient implies a greater impact of GP workforce leaving patient care). Second, we explored which practices might be at particular risk of a marked increase in local population. This was done by inflating the predicted adjusted weighted list size. The following scenarios were explored:

A. The coefficient for expected proportion of GPs remaining in patient care increased by 2 (equivalent to a 22% increase in the odds of being in supply–demand imbalance

For peer review only

when 10% of GPs are expected to leave representing a modest increase in the difficulty of recruiting GPs);

B. The coefficient for expected proportion of GPs remaining in patient care increased by 4 (equivalent to a 49% increase in the odds of being in supply–demand imbalance when 10% of GPs are expected to leave representing a substantial increase in the difficulty of recruiting GPs);
C. The predicted adjusted weighted list size increased by 20%;
D. The predicted adjusted weighted list size increased by 40%;
E. A modest increase in difficulty recruiting GPs combined with a 20% increase in list size (a and c combined); and
F. A substantial increase in difficulty recruiting GPs combined with a 40% increase in list size (b and d combined).

For each of these scenarios, practices were rated according to relative risk (i.e. top 25% were labelled "high relative risk" as above) and absolute risk. The relative risk cut-offs in the baseline scenario were used for absolute risk cut-offs in the other scenarios.

Results

Mapping the current situation

A total of 6,398 practices in England had available data on all data items and had list sizes>1000, of which 371 were in South West England. Practices with GPPS access scores (our proxy for ability to meet patient demand) in the highest scoring third nationally were over-represented in South West England, with 57% of practices in this region falling in that category. There was also an under-representation of South West practices nationally in respect of workload (only 22% of practices in the region were classified as in the third of practices nationally with the highest workload). As a result, the percentage of practices defined as currently being in under-supply was considerably lower in South West England (5.1%) than in England as a whole (13.5%).

There was no evidence that list size varied between those practices in under-supply and other practices in South West England (Table 1). However, there was evidence that practices in under-supply had fewer FTE GPs. Together, these findings indicate that observed differences in workload are driven more by the supply of GP workforce than the demand of the registered patient population. Practices in undersupply also had a higher ratio of nurse FTE to GP FTE, served more deprived populations, had lower patient experience scores, had fewer patients over the age of 65, and were more likely to be in urban areas.

For peer review only

Predictive risk model

The regression coefficients for the logistic model are shown in Table 2 Predictive risk model coefficients estimated using 2012 data where possible to estimate the independent association with 2016 undersupply status. A negative coefficient implies a reduced risk of future undersupply as the value of the variable increases. We note the interaction between the expected proportion of GP FTE still working in patient care in 5-years' time and the ratio of nurse FTE to doctor FTE had a relatively large p-value (0.177). In initial modelling (before excluding practices on the basis of data quality) this interaction variable had a smaller p-value (0.06) indicating some evidence that it was worth including. When exclusions were applied, the coefficient did not change meaningfully. This fact, combined with the *a-priori* expectation that the effect of expected future GP workforce would be dependent on recruitment, provided support to maintain the interaction term.

For peer review only

Figure 1 shows the ROC curve derived from the development model (i.e. 2012 covariates and 2016 outcome). The area under the curve is 0.759. The ROC curve from a model only including the defining factors (GPPS access scores and adjusted weighted list size per FTE) was 0.718, suggesting that the additional variables included in our model provided a modest, but meaningful, improvement in predictive value.

Future risk predictions

Applying the risk prediction model to data from 2016, seeking to predict the risk of future supply–demand imbalance for individual practices in South West England, we obtained risk scores for 368 practices with available data remaining after applying exclusions. The median probability of future supply–demand imbalance across practices was 5.4% (IQR 2.8% to 10.0%). In total 40 (10.9%) practices had a risk greater than 20%, and 12 (3.3%) had a risk greater than 50%. Table 3 shows the characteristics of those practices in South West England classified as high risk (top 25% of practices, corresponding to an absolute risk of 10% or greater) of being in a state of under-supply compared with other practices. In contrast to the current situation shown in Table 2, there was no evidence (p=0.445) that the total GP FTE varies between high/other risk classification. There was evidence, however, that all other descriptive factors varied between the two groups. Practices at “high risk” of future supply–demand imbalance tended to currently have larger list sizes, to have a higher proportion of nurses in the workforce, to serve more deprived and younger populations, have considerably worse GPPS scores, and were more likely to be in urban areas.

Stress testing scenarios

Figures 2 and 3 illustrate the changes to the relative and absolute risk of future under-supply under different stress testing scenarios. In this figure, each practice is represented by a horizontal bar. The vertical ordering of each practice is the same in each scenario, and is based on the rank ordering of each practice according to the baseline risk prediction. For each scenario, the colouring of every practice’s horizontal bar illustrates the relative or absolute risk classification (Figure 2 and 3 respectively) such that changes in colour indicate changes in risk classification.

Comparing the baseline prediction (where responses to the career intention survey were used to predict the future GP workforce remaining in patient care), with a prediction using only GP age and gender, very little difference was observed in practices categorised as being either at “high relative risk” or “high absolute risk” of undersupply (seen in Figure 2 as limited reclassification of practices, correlation of ranks=0.999).

In general, practices classified as being at “high relative risk” remained so under scenario A (modest increase in the difficulty of GP recruitment to replace those leaving - correlation in ranks between scenario a and baseline=0.97). However, there was a dramatic increase in the number of practices with a predicted absolute risk of future undersupply greater than 10% (seen as an increase in the number of practices coloured red Figure 2, scenario A).

For peer review only

There was an even greater disturbance in the classification of practices under scenario B (illustrating the recruitment of GPs was becoming much harder), though the reclassification in terms of relative risk was still relatively modest (Figure 2, scenario B, correlation in ranks between scenario B and baseline=0.90). Conversely, the reclassification in terms of absolute risk (Figure 3, scenario B) was significantly greater; the majority of practices had a predicted risk above 10%.

Increasing the projected practice population resulted in only modest changes in respect of which practices are classified as being at “high relative risk”. Only a small relative increase was seen when comparing scenarios C and D with the baseline predictions (Figure 2 correlation in ranks between scenario C and baseline=0.99 and scenario D and baseline=0.98). However, substantial changes were seen in the number of practices with an absolute risk of undersupply greater than 10% (Figure 3, scenarios C and D). Combining the effect of scenarios A and C resulted in relative risk classifications closer to the baseline predictions than scenario A alone. However, in terms of absolute risk, more practices had a risk greater than 10% (Figure 3, scenario A and scenario C).

When scenario B and scenario d were combined (illustrating a situation where it was much harder to recruit GPs combined with an increased practice population of 40%) it was evident that nearly all practices (88%) exceeded 10% absolute risk of supply–demand imbalance within 5 years, with only 9 (2.4%) practices classified as being at “low absolute risk” using the cut-offs derived from the baseline predictions.

Discussion

Summary of main findings

We developed a predictive risk model informed by historical data which could be used to predict an individual practice’s future risk of being in a state of GP workforce undersupply. The model produced a range of risk scores attributable to practices across South West England, and has a fair to good discriminatory ability in this context (based on the ROC curve analysis). Applying this to current data suggests that the practices at highest risk of future undersupply of GP workforce have, on average: larger patient lists; employ more nurses relative to doctors; serve more deprived and younger populations; and have considerably worse patient experience ratings.

We modelled scenarios where the recruitment of GPs becomes harder and/or practice populations increase dramatically beyond what would be expected from historical trends (for example, through a new housing development). These scenarios do increase absolute risk dramatically, but by and large, it is the same practices in all scenarios that are at highest risk of future undersupply of GP workforce. This almost certainly reflects the fact that those most likely to have problems in the future are those currently experiencing difficulties. This can be seen in the relatively good predictions from a simple model including only defining factors (i.e. workload per FTE GP and GPPS patient access scores) which had an area under

For peer review only

the ROC curve which was not substantially less than the full model. In particular, we found that inclusion of findings from our own survey of GPs' career intentions had very little impact on the predictions, compared with using expected future workforce projections based only on routinely available data regarding GPs' gender and age.

Strengths and weaknesses

Strengths of this work include the comprehensive use of freely available data as well as the exploratory use of a census survey of career intentions of GPs in the region. The main strength is the novel development of factor weightings based on routinely available historical data. However, we recognise that this assumes that factors driving changes are constant from the historical time period of model development to the future time period of prediction. This is unlikely to be the case given recent problems in GP workforce recruitment and retention in the UK [4]. To this end we have modelled what might be expected if recruitment was harder than it has been historically, and if there were substantive increases in the practice population. These scenarios may be more reflective of what we might expect going forward.

The main weakness of this work concerns our ability to distinguish in what situations, and in which practices, future GP workforce leaving patient care will impact the level of continuing GP workforce and its ability to meet patient demand. For practices that do not encounter problems in recruiting GPs, retiring GPs pose much less of an issue than for practices where recruitment is difficult. Here we relied on the level of nurse staffing in a practice as a proxy for recruitment issues; importantly, this means the association of more nurses with at-risk practice status is likely to be attributable to practices being unable to fill GP vacancies, not that more nurses per se puts a practice at risk. A more direct measure of recruitment problems which was consistently and widely collected (such as duration of advertising for vacant posts, using a consistent methodology to track this) would be expected to provide a better model. Unfortunately, no robust freely available measure exists. The NHS GP census does collect data on time to fill vacancies [17] and existing unfilled vacancies. However, these data are not freely available, and, furthermore, are not mandatory for completion by practices.

Another weakness was that historical workforce data were not available in the same detail as current data. This meant that future workforce predictions using historical data would not be as accurate as those using current data. These inaccuracies would lead to a loss of power, and potentially an attenuation of the associated regression coefficients. This may explain the low statistical significance of associated coefficients in the model.

Finally, we note that our assessment of the performance of our model was made on the same data the model was developed on, and thus is likely to overestimate the performance of the model. Validation of the future risk predictions would be welcome, but can only be undertaken in 5 years' time.

For peer review only

Implications

We have demonstrated that it is possible to make reasonable predictions of an individual general practice's future risk of undersupply of GP workforce with respect to its patient population. With ongoing GP workforce issues in the UK, local models are being developed to identify potentially "at-risk" practices [23]. However, unlike the model we present here, it is not clear to what extent these models are evidence-based or to what extent their limitations are recognised by the users of the models or even what is meant by "at risk".

Whilst the model we present here is set in the context of UK primary care, the general approach could be applied to other settings and in other locations. In all cases the predictions will be inherently limited by the quality of available data. Improvements in data quality going forward will help the situation in the UK, particularly if data are released on GP recruitment. However, it will be some time before robust historical data exist that can be used for the model development process outlined here. If models such as the one outlined here are to be produced and used, it is important that high-quality data continue to be collected. The predictions produced by this model and similar models may facilitate targeting of interventions to retain and attract GP workforce either in specific practices, or in specific regions currently at high risk of problems driven by workforce supply. Although our model provides reasonable discrimination, much could potentially be achieved by focussing efforts on those practices currently experiencing difficulties.

Whilst a policy of targeted interventions may have value, we find that most practices are likely to be at a high risk of workforce undersupply when faced with a substantial increase in demand from an increased patient population combined with major difficulties in recruiting GPs. As such, local knowledge of drivers of increased practice populations, such as housing developments, will be key to being able to suitably apply targeted interventions. Even in South West England where workload and the ability to meet patient demand are better than in England overall, most practices are currently vulnerable to recruitment challenges, and will remain so going forward. Given this, national or broad regional policies and strategies may be more effective than targeted ones, especially if there is limited knowledge on how local populations are likely to evolve.

Contributions. GA, MGC and NM contributed to study design, analysis, and writing of the paper. All other authors contributed to study design and writing of the paper.

For peer review only

1. BMJ. Seven days in medicine: 1-7 February 2017. *BMJ (Clinical research ed)* 2017;356 doi: 10.1136/bmj.j668
2. Hobbs FR, Bankhead C, Mukhtar T, et al. Clinical workload in UK primary care: a retrospective analysis of 100 million consultations in England, 2007–14. *The Lancet* 2016;387(10035):2323-30.
3. Sansom A, Terry R, Fletcher E, et al. Why do GPs leave direct patient care and what might help to retain them? A qualitative study of GPs in South West England. *BMJ open* 2018;8(1) doi: 10.1136/bmjopen-2017-019849
4. Roland M, Everington S. Tackling the crisis in general practice. *BMJ (Clinical research ed)* 2016;352 doi: 10.1136/bmj.i942
5. Kash BA, Ogden P, Popp E, et al. Exploring future models of primary care for Texas. *International Journal of Innovation Science* 2017;9(4):435-54. doi: doi:10.1108/IJIS-01-2017-0005
6. Miani C, Pitchforth E, Ellen N. Choice of primary care provider: a review of experiences in three countries: Policy Research Unit in Policy Innovation Research (PIRU), 2013.
7. Spitzer WO, Sackett DL, Sibley JC, et al. The Burlington randomized trial of the nurse practitioner. *The New England journal of medicine* 1974;290(5):251-6. doi: 10.1056/nejm197401312900506 [published Online First: 1974/01/31]
8. Freund T, Everett C, Griffiths P, et al. Skill mix, roles and remuneration in the primary care workforce: who are the healthcare professionals in the primary care teams across the world? *International journal of nursing studies* 2015;52(3):727-43. doi: 10.1016/j.ijnurstu.2014.11.014 [published Online First: 2015/01/13]
9. Laurant M, Reeves D, Hermens R, et al. Substitution of doctors by nurses in primary care. *The Cochrane database of systematic reviews* 2005(2):Cd001271. doi: 10.1002/14651858.CD001271.pub2 [published Online First: 2005/04/23]
10. Paddison C, Abel G, Campbell J. GPs: working harder than ever. *The British journal of general practice : the journal of the Royal College of General Practitioners* 2018;68(670):218-19. doi: 10.3399/bjgp18X695873 [published Online First: 2018/04/28]
11. NHS England. GP Patient Survey: NHS England; [Available from: https://gp-patient.co.uk/surveys-and-reports accessed 05/03/2018.
12. The Centre for Workforce Intelligence. In-depth review of the general practitioner workforce, 2014.
13. Campbell JL, Salisbury C. Research into practice: accessing primary care. *British Journal of General Practice* 2015;65(641):e864-e68. doi: 10.3399/bjgp15X688057
14. Campbell J, Smith P, Nissen S, et al. The GP Patient Survey for use in primary care in the National Health Service in the UK – development and psychometric characteristics. *BMC Family Practice* 2009;10(57):Doi: 10.1186/471-2296-10-57.
15. NHS Digital. General and personal medical services, England 2002-2012 as at 30 September 2012 [Available from: http://content.digital.nhs.uk/searchcatalogue?productid=10382&topics=1%2fWorkforce%2fStaff+numbers&kwd=GP+staff&geo=England&sort=Relevance&size=10&page=2#top accessed 31/07/2017 2017.
16. NHS Digital. General and personal medical services, England 2003-2013 as at 30 September 2013 [
17. NHS Digital. General and personal medical services, England September 2015-March 2016, Provisional Experimental statistics 2016 [Available from: http://content.digital.nhs.uk/catalogue/PUB21772 accessed 31/07/2017 2017.

For peer review only

18. Fletcher E, Abel GA, Anderson R, et al. Quitting patient care and career break intentions among general practitioners in South West England: findings of a census survey of general practitioners. *BMJ open* 2017;7(4) doi: 10.1136/bmjopen-2017-015853
19. Public Health England. National General Practice Profile 2013 [Available from: http://fingertips.phe.org.uk/profile/general-practice accessed August 2104 2014.
20. Office for National Statistics. Subnational population projections for England: 2014-based projections 2014 [Available from: https://www.ons.gov.uk/peoplepopulationandcommunity/populationandmigration/populationprojections/bulletins/subnationalpopulationprojectionsforengland/2014basedprojections accessed 31/07/2014 2017.
21. Paddison C, Elliott M, Parker R, et al. Should measures of patient experience in primary care be adjusted for case mix? Evidence from the English General Practice Patient Survey. *BMJ Quality & Safety* 2012
22. Abel GA, Saunders CL, Lyratzopoulos G. Cancer patient experience, hospital performance and case mix: evidence from England. *Future oncology (London, England)* 2014;10(9):1589-98. doi: 10.2217/fon.13.266 [published Online First: 2013/12/18]
23. Healthy London Partnership. Primary Care (GP) Workforce Model 2016 [Available from: http://healthylondon.org/hlp-archive/sites/default/files/The%20Primary%20Care%20Model%20Step-by-Step%20Guide.pdf accessed 05/03/18.

For peer review only

Table 1 Comparison of practices in South West England defined as in undersupply with other practices in the region.

	Under-supply (n=19)			Other (n=352)			
	**median**	**25%**	**75%**	**median**	**25%**	**75%**	**p value***
List size	9264	5361	11576	7598	5270	11077	0.448
Adjusted weighted list size	8959	5212	12287	8099	5638	11570	0.550
GP FTE	3.1	2	5.1	4.7	3.2	6.6	0.012
Ratio nurse/GP FTE	0.8	0.7	1	0.5	0.4	0.7	<0.001
Index of Multiple Deprivation	25.7	20.2	30.9	18.7	13.5	24.4	0.003
GPPS access‡	0.2	0.1	0.2	0.7	0.5	0.9	<0.001
GPPS continuity‡	0.2	0.2	0.3	0.6	0.4	0.8	<0.001
GPPS satisfaction‡	0.2	0.1	0.4	0.7	0.5	0.9	<0.001
% over 65	16.8	13.3	21	22.6	17.6	26	0.004
**Setting**	**n**	**%**		**n**	**%**		**p value†**
Urban practices	17	6.8		232	93.2		0.042
Rural practices	2	1.6		120	98.4		

*** from Mann–Whitney test**
† from Fisher's exact test
‡GPPS scores used were case-mix adjusted log-odds ratios relative to the average practice nationally
** IMD scores are given (rather than ranks) with higher scores indicating higher levels of deprivation.

For peer review only

Table 2 Predictive risk model coefficients estimated using 2012 data where possible to estimate the independent association with 2016 undersupply status.

Data type	Variable	Note on units	Logistic regression coefficient (95% CI)	p-value
GP Patient Survey Scores*	Access	Random effect (log-odds ratio) from logistic case-mix adjustment model	-0.96 (-1.21, -0.70)	<0.001
	Continuity of care		-0.09 (-0.25, 0.07)	0.274
	Overall Satisfaction		-0.48 (-0.70, -0.27)	<0.001
Baseline Workforce†	Ratio of nurse FTE to GP FTE		1.02 (-0.05, 2.09)	0.062
	Adjusted Weighted List Size per GP FTE	Per 1000 patients per GP FTE	0.40 (0.18, 0.62)	<0.001
	Total GP FTE		-0.17 (-0.25, -0.10)	<0.001
	Ratio of "Other" GP FTE to total GP FTE		0.65 (0.32, 0.98)	<0.001
Rurality Setting‡	Urban practice		Reference	0.404
	Rural practice		-0.13 (-0.43, 0.17)	
Index of Multiple Deprivation – practice in quintile‡	1 – least deprived		Reference	<0.001
	2		0.02 (-0.29, 0.32)	
	3		0.13 (-0.16, 0.42)	
	4		0.57 (0.29, 0.85)	
	5 – most deprived		0.36 (0.06, 0.66)	
Projected quantities	Adjusted Weighted List Size**	Per 1000 patients	0.14 (0.10, 0.18)	<0.001
	Proportion of GP FTE still in patient care*	Varies from 0 to 1	0.38 (-0.78, 1.54)	0.520
	Proportion of GP FTE still in patient care x Ratio of nurse FTE to GP FTE*		-1.01 (-2.48, 0.46)	0.177
Constant			-4.15 (-5.10, -3.21)	<0.001

*Data from 2012
†Data from 2012 except nurse data which were from 2013
‡IMD data from 2016 for variable where this status is expected to remain relatively constant over time
**Actual list size from 2016 rather than projected list size based on 2012 data as pre-2012 data did not allow projections comparable to those which were made with more current data looking forwards.

For peer review only

Table 3 Differences between practices identified at high risk of future undersupply and other practices assuming a baseline scenario.

	High risk (n=92)			Other (n=276)			
	**median**	**25%**	**75%**	**median**	**25%**	**75%**	**p value***
List size	10625	7732	13195	6915	4941	10206	<0.001
Adjusted weighted list size	11133	7369	13252	7398	5251	10615	<0.001
GP FTE	5	3.1	6.6	4.5	3.1	6.6	0.445
Ratio of nurse FTE to GP FTE	0.7	0.5	1	0.4	0.4	0.6	<0.001
IMD	25.6	18.7	31.7	17.6	13.1	22.2	<0.001
GPPS access‡	0.4	0.2	0.6	0.8	0.6	0.9	<0.001
GPPS continuity‡	0.3	0.2	0.5	0.7	0.5	0.9	<0.001
GPPS satisfaction‡	0.4	0.2	0.6	0.7	0.5	0.9	<0.001
% over 65	18.3	14.1	23.4	23.2	18.5	26.5	<0.001
Setting	**n**	**%**		**n**	**%**		**p value†**
Urban practices	77	31.3		169	68.7		<0.001
Rural practices	15	12.3		107	87.7		

*** from Mann–Whitney test**

† from Fisher's exact test

‡GPPS scores used were case-mix adjusted log-odds ratios relative to the average practice nationally

For peer review only

Figure 1 ROC curve for the predictive risk model based on the national historical data used to build the model.

For peer review only

*Risk prediction as for baseline, but using age and gender of GPs alone rather than including responses to the career intentions survey. In each case the practices are ordered by the baseline scenario.

Figure 2 Rating of practices in South West England from different risk prediction scenarios a-d using cut-offs defined by the quartiles of each prediction (relative risk).

For peer review only

*Risk prediction as for baseline, but using age and gender of GPs alone rather than including responses to the career intentions survey. In each case the practices are ordered by the baseline scenario.

Figure 3 Rating of practices in South West England from different risk prediction scenarios a-d using cut-offs defined by the quartiles of the baseline prediction (absolute risk).

For peer review only

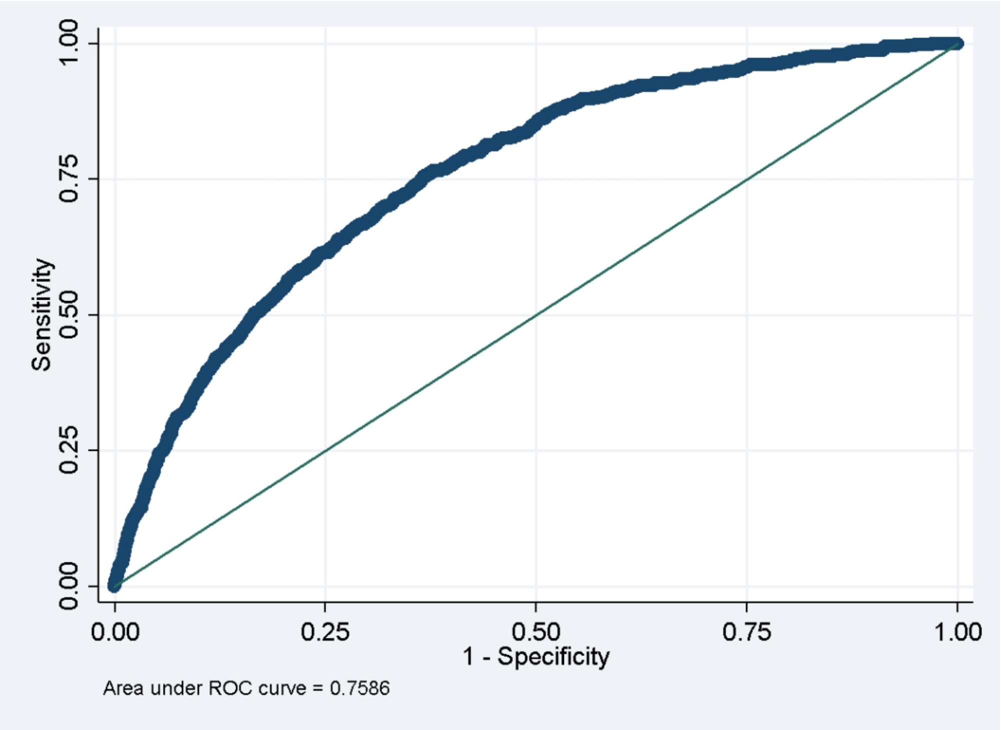

Figure 2 ROC curve for the predictive risk model based on the national historical data used to build the model.

101x73mm (300 x 300 DPI)

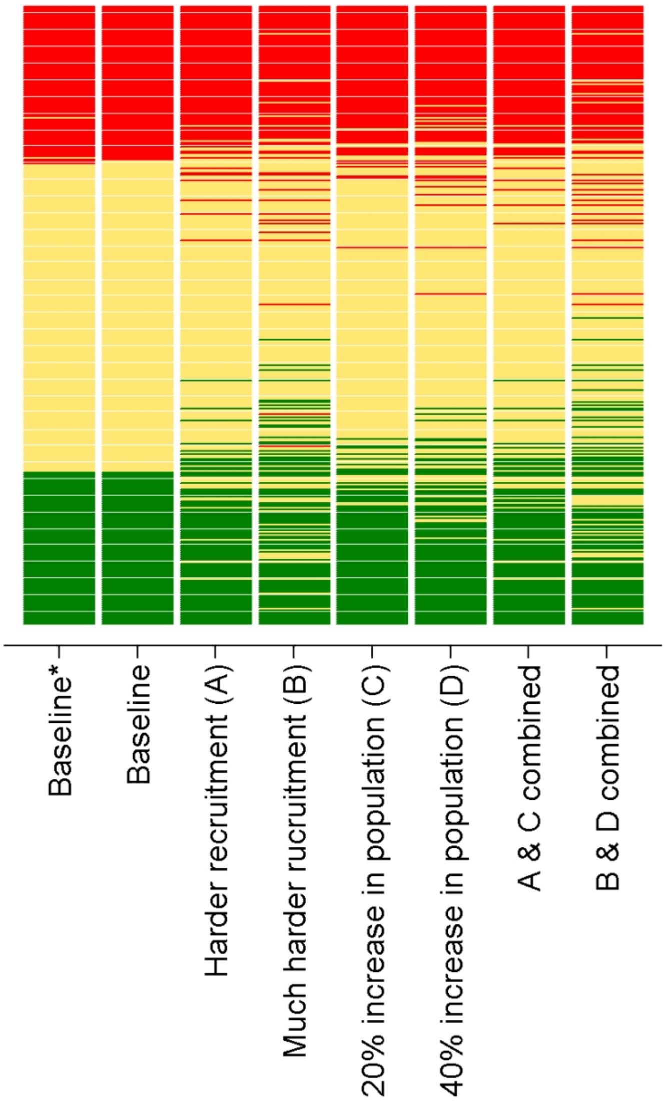

Figure 3. Rating of practices in South West England from different risk prediction scenarios a-d using cut-offs defined by the quartiles of each prediction (relative risk).

95x127mm (300 x 300 DPI)

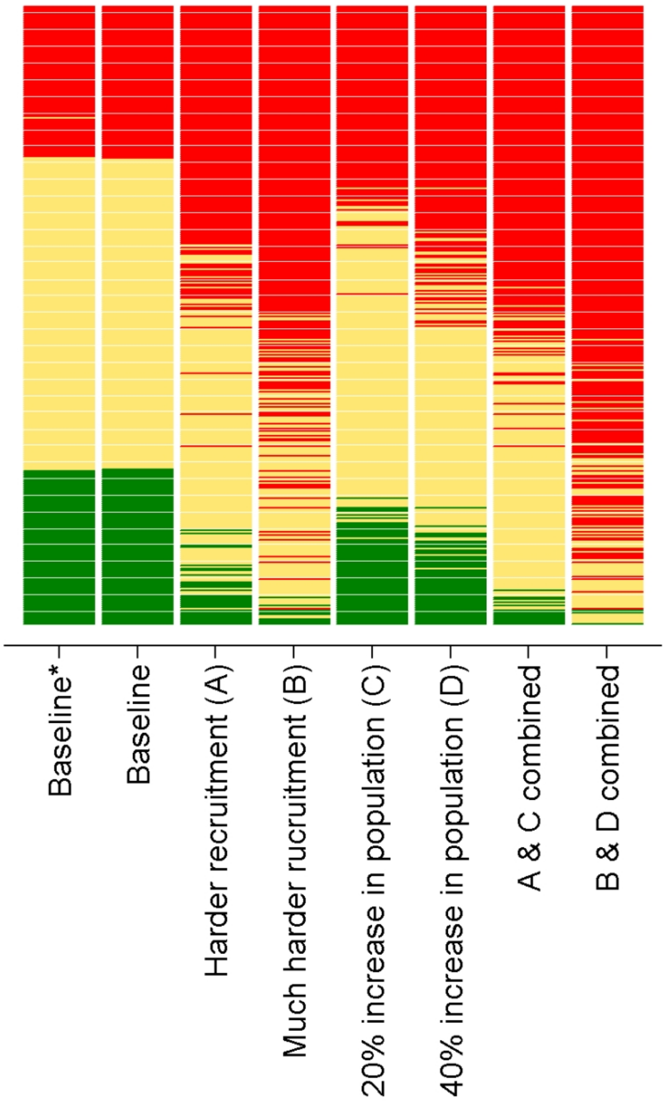

Figure 4 Rating of practices in South West England from different risk prediction scenarios a-d using cut-offs defined by the quartiles of the baseline prediction (absolute risk).

95x127mm (300 x 300 DPI)

Appendix 1 - Data sources and preparation

Except where specified, national data for England were obtained and processed. A summary of data sources and data flow used in the modelling process is presented in Appendix 2.

GP Patient Survey

The General Practice Patient Survey (GPPS) is a national postal survey of patients' experience of primary care in England. Patients from practices that are known from prior surveys to have low response rates are oversampled. Full details of the sampling strategy are published elsewhere.[1] We used data from the 2011/12 and 2015/16 surveys. The contents of the survey have remained largely consistent over this time period. Response rates were 38% in 2011/12 (1,037,946 responses) and 39% in 2015/16 (836,312 responses).

We made use of three items from the GPPS reflecting access, continuity of care and overall experience. For patient's experience of access the following question was used:

> "Last time you wanted to see or speak to a GP or nurse from your GP surgery:" "Were you able to get an appointment to see or speak to someone?"

Responses of "Yes" and "Yes, but I had to call back closer to or on the day I wanted the appointment" were coded as a positive response and responses of "No" were coded as a negative response. Responses of "Can't remember" were treated as uninformative and excluded from the analysis.

The item on ability to see a preferred doctor is taken as a proxy measure for continuity of care:

> "How often do you see or speak to the GP you prefer?"

Responses of "Always or almost always" and "A lot of the time" were coded as a positive response and responses of "Some of the time" and "Never or almost never" were coded as a negative response. Responses of "Not tried at this GP surgery" were treated as uninformative and excluded from the analysis.

Finally, an item capturing data on the patient's overall experience of care is included:

> "Overall, how would you describe your experience of your GP surgery?"

Responses of "Very good" and "Fairly good" were coded as a positive response and responses of "Neither good nor poor", "Fairly poor" and "Very poor" were coded as a negative response. There were no uninformative options for this question.

Due to certain patient groups tending to give more positive responses in patient surveys, case-mix adjusted practice scores were created. This was achieved using mixed effects logistic regression adjusting for patient age, gender, and ethnicity, presence of a long-term condition, and deprivation

For peer review only

(using the Index of Multiple Deprivation [IMD], an area based measure assigned according to the patient's residential postcode[2,3]) and a random intercept for practice. The case-mix adjustment provides scores for individual practices based on a standardised mix of patients. The case-mix adjusted scores were used in the form of log-odds ratios relative to the average practice nationally.

Workforce

Workforce data were obtained from NHS Digital and related to the GP Census data taken as at 30 September 2012, 2013 and 2016.[4-6] Each dataset gave the headcount of GPs in 5-year age-bands for each practice. The 2012 dataset contained total GP headcount by gender as did the 2016 dataset. In the 2016 dataset additional detail of GP FTE by gender was provided. Both datasets contained total GP FTE as well as GP FTE broken down by GP role. We also extracted the total nurse FTE from the 2016 dataset. As nurse FTE data were not available in 2012, the relevant data were extracted from the 2013 dataset in its place. From these data two further variables were derived: the ratio of nurse FTE to GP FTE; the ratio "other" category FTE to total GP FTE (where "other" is assumed to mostly be Locum GPs given that GP registrars, salaried GPs, and those on retainer schemes are captured in other categories). These data were also used in the derivation of workload and the predicted remaining future workforce.

Practices with less than 0.5 FTE GP (38 out of 7,484 practices in 2012 data and 41 out of 6,709 practices in 2016 data) were excluded from all analyses on the basis that such a low staff record indicated either that these were unusual practices or that the workforce data were in error. In the former case such unusual practices are not the focus of this work and in the latter case, erroneous inferences may have been made if they had been included.

GP quitting intentions

To predict remaining future workforce we utilised self-reported GP intentions to cease practice collected through a survey which formed part of the ReGROUP project and has been reported [7]. Briefly, a questionnaire was administered to all active GPs in South West England enquiring about their intentions to cease/interrupt practice in the next 2 and 5 years. We combined responses to two questions:

> "How likely is it that you will permanently leave direct patient care within the next 5 years?"
> "How likely is it that you will take a career break (or another career break) within the next 5 years?"

Each question had response options of "Very Likely", "Likely", "Unlikely" and "Very unlikely". Where GPs gave different response options for these two questions, the response with the highest likelihood

For peer review only

of cessation or interruption was taken. This reflects the most likely chance of impact to future GP workforce in the next 5 years. We also used respondents' answers to the question:

> "In your current/most recent direct patient role, how many sessions do/did you work in a typical week?"

Survey responses provided data from which an estimate of each responder's current FTE work commitment could be calculated. Working eight sessions per week was taken as 1 FTE, consistent with the approach used in the GP census.[6] When more than eight sessions was given as a response the FTE was capped at 1. If more than 24 sessions was given as a response it was assumed the question had been answered incorrectly and the data were treated as missing. Data for all GPs surveyed on age, gender and affiliated practice were obtained from the Performers List.

Practice rurality and deprivation

Practice rurality was contained within the GPPS 2016 dataset and was based on an Office for National Statistics (ONS) categorisation of the postcode of the practice. We used a rural/urban version of this categorisation. Practice deprivation score was obtained from Public Health England and was based on the 2015 IMD. Individual patient IMD is based on each patient's residential postcode, and the practice score is the mean of individual patient scores using all patients registered at the practice.[8]

Practice registered population

Data on the registered populations for each general practice were obtained from NHS Digital for each quarter from April 2014 to April 2016 (9 datasets); as well as April 2012. These datasets provided the count of patients in each gender by 5-year age-band (with the highest age-band being 95 and over). We aggregated the top three age-bands resulting in a top age-band category of 80+ years.

The April 2012 and April 2016 datasets were used to calculate list sizes weighted for the demographics of the populations and adjusted for deprivation. The reason for weighting for patient demographics is that certain types of patients (older, female and very young) place a higher demand on practices than others. The adjustment for deprivation acknowledges that deprived populations have higher health needs than less deprived populations with a similar demographic profile. To calculate weighted list sizes the practice populations were weighted according to the average time spent consulting with patients in 14 age by gender groups in 2013/14 according to a recent study based on routine patient records from 674 practices.[9] Weighted lists sizes ( ) were then normalised so the total population across the country remained unchanged. These weighted list sizes are taken as a measure of workload on the basis that they represent a measure of the expected time spent consulting. This assumes that, on average, patients in the same demographic group require the same amount of consultation time. Because age and gender do not capture the health status of the population the weighted list sizes

For peer review only

were then adjusted for deprivation (IMD decile, , taking a value between 1 and 10, based on all practices in England) assigning a 10% weighting to a deprived population. The adjusted weighted population will thus be given by

$$= . \quad + . \left(\frac{\quad}{\quad} \quad \right) \qquad 1$$

This approach is intended to mirror that used in the current resource allocation to CCGs. However, the CCG allocations do not use deprivation, but rather make use of a measure of premature mortality (the <75 standardised mortality ratio, which is the ratio of mortality in under 75 year olds to that expected given the age and sex composition of the CCG population). We chose to use deprivation here as standardised mortality ratios are not published for individual practices.

The adjusted weighted list sizes were divided by the total GP FTE to obtain a measure of workload per GP. Initial inspection of the workload figures showed that the distribution contained some infeasibly large and small values. These may have arisen from errors in either the workforce or practice population data. Unfortunately, there was no clear separation between typical values and those that were infeasible. A pragmatic approach was taken whereby practices in the top and bottom 2.5% of the distribution were excluded from all further analysis. This exclusion took place following the removal of practices with less than 0.5 GP FTE.

The data from April 2014 to April 2016 were used in the prediction of future practice populations along with the subnational population projections described below.

Subnational population projections

We made use of ONS subnational population projections at the level of CCGs (used to inform local planning of healthcare and other public services)[10] in the prediction of future practice populations (see below). The subnational ONS projections are demographic, trend-based projections that indicate the ‘likely levels of future population’ and are currently produced every 2 years; they present projections for every year for the next 25 years from the base year.[11] The underlying data sources that inform the calculations include: national population projections; registration of births and deaths (General Registrar Office); armed forces data (MOD); data extracts from the Patient Register Data System (NHS); student location data (Higher Education Statistics Agency [HESA]); and data on asylum seekers (Home Office). Adjustments were then made to the datasets for factors such as assumed fertility and mortality rates, internal and international migration. However, the projections do not account for local development aims and policies, economic factors, and indeed any international

For peer review only

factors that are likely to affect the UK population.[11] We extracted projected populations for 2021 for the eight CCGs within the scope of the ReGROUP project: NHS Bath and North East Somerset CCG; NHS Kernow CCG; NHS North, East, West Devon CCG; NHS South Devon and Torbay CCG; NHS Bristol CCG; NHS North Somerset CCG; NHS Somerset CCG; NHS South Gloucestershire CCG. Projections are made in 5-year age-bands for each gender. As with practice population data the upper age groups were combined to form an 80+ age-band.

Projecting future workload

Our projections of future practice workloads were based on the number of patients registered at each of the 423 GP practices in South West England, in 5-year age bands, split by gender combined with subnational population projections from the ONS as described above. The approach comprises the following five steps.

1. Assess congruency of ONS predictions with list size
   ONS subnational population projections were compared with GP list size data aggregated to CCG level for 2014, 2015 and 2016. This provided an assessment of the degree to which ONS predications reflect the actual GP list size data in those years. This difference between the two data sources is most likely due to “list inflation”, caused by patients that have not been removed from the list following death, dual registrations for patients when moving homes or by a registered patient’s failure to complete the national census.[12] Given that the average consultation times used to weight the populations (described above) are based on registered patients, we did not consider it appropriate to resize practice list sizes to reflect the identified difference.
2. Calculate the proportion of CCG population registered at each GP practice
   For each practice, and for each age-band by gender stratum, we identified the number of patients registered with the practice and the expected number of patients within a CCG for nine time-points between April 2014 to April 2016. This allowed us to derive the proportion of the total CCG population by gender/age-interval registered at each practice. If the number of practices in a CCG is declining over time we might expect the proportion of the CCG population to be rising at the remaining practices.
3. Quantify trends in the proportion of the CCG population registered at each general practice
   The data from step 2 were used as the outcome variable in a logistic regression model that included a linear term for time as well as a categorical variable for quarter to quantify trends. A separate regression model was used for each practice by age-band by gender strata.
4. Determine projected count of patients

We used the resultant regression equation to predict the proportion of CCG patients by practice/gender/age-interval for five years beyond the final data point. Multiplying this proportion by the ONS predicted population for the same time point gives a projected count of patients.

5. Project adjusted list size
The projected populations were used to create a projected adjusted weighted list size using the same algorithm used above for observed populations.

For peer review only

Predicting remaining future workforce

When predicting future workforce (supply) we concentrated on predicting what fraction of the existing workforce will remain available to the practice in 5 years' time. We did this in two principal ways: i) based on the age and gender of GPs at the practice; ii) based on responses to the ReGROUP survey of GP quitting intentions. Predictions are made based on 2012 data and 2016 data (with the survey only being available for the 2016 data).

Approach 1 – Using the age and gender profile of GPs at each practice.

Previous work has identified the probability that GPs of a given single year of age and gender will remain in the workforce 12 months later.[1] By multiplying these probabilities over five consecutive single year age bands we obtain the probability that GPs of a given single year of age and gender will remain in the workforce in 5 years' time. As the routinely available GP census data (p.129) is only available in five-year age-bands, we take the mean of these 5-year probabilities over the 5-year age-bands used in the GP census data. Unfortunately, the GP census data published at practice level gives data by either age or gender, but not both. Furthermore, data by age is only given in terms of headcount, as is data by gender in 2012 (data by gender is given in terms of headcount and FTE in 2016). Thus we adopted the following procedure to estimate remaining workforce.

1. Using the probabilities described above, identify the probability that each GP in the practice will remain in patient care in 5 years' time based on their age-band assuming they are male.

2. Calculate the mean of these probabilities over all GPs in the practice.

3. Repeat steps 1 and 2 assuming they are female.

4. Take a weighted average of the probabilities obtained in steps 2 and 3 weighted by the FTE of male and female GPs in the practice (in 2012 data headcount by gender is used instead).

The resulting probabilities can then be interpreted as the proportion of GP FTE which is expected, on average, to remain at the practice in 5 years' time.

Approach 2 – Using the ReGROUP survey responses.

An alternative approach used in the forecasting utilised the results of the ReGROUP survey where all GPs in South West England were asked about their future career intentions. For GPs who responded to the survey (67%) we used both stated career intentions, stated FTE (as described above), and information on age and gender. For non-responders we simply used age and gender information (provided within the Performers List). To incorporate the survey responses we made use of odds ratios estimated from a previous study which linked stated quit intentions to working status 5 years later and adjusted for age and gender.[59] Odds ratios for their 5-point scale are mapped to our 4-point scale by ignoring the middle (neutral) option.

1. It proved difficult to map the ReGROUP survey responses to the NHS GP census data (due to inconsistent age, gender and FTE information between the two data sources). Therefore, in this methodology, the GP census data are only used in the estimation of FTE of survey non-responders based on difference between the total GP FTE (GP census data) and the total FTE stated by responders linked to each practice within the Performers List. This was done using the following method. We calculated the difference between the total GP FTE given in the GP census data and the stated total GP FTE of responders to the survey linked through the Performers List to each practice in the study. The assumed FTE of non-responders was this difference divided by the number of non-responders linked to the practice. Where this difference was greater than the number of FTEs, the non-responders were assigned an FTE of 1. Where this difference was negative, non-responders were assigned an FTE of 0.

2. We then calculated probabilities of remaining in patient care for the forthcoming 5 years. For the survey non-responders, we assigned a probability of remaining in patient care using the same method as in approach 1 but based on the individual GP's gender and current year of age taken from the Performers List (rather than the GP census). For responders, we similarly assigned a probability of remaining in patient care based on the individual GP's age and gender and then adjusted that probability using the following odds ratios (Calculated from Hann et al. [13] but changing the baseline to the neutral category) "Very likely" 1.94, "Likely" 1.3, "Unlikely" 0.70, and "Very unlikely" 0.43.

For peer review only

3. For each practice, we then took the weighted average of the probabilities obtained in step 2 (over GPs associated with a practice, weighted according to their FTE.

The resulting probabilities can then be interpreted as the proportion of GP FTE that would be expected, on average, to remain at the practice in 5 years' time.

References

1. NHS England. GP Patient Survey. https://gp-patient.co.uk/surveys-and-reports

2. Paddison C, Elliott M, Parker R, Staetsky L, Lyratzopoulos G, Campbell JL, et al. Should measures of patient experience in primary care be adjusted for case mix? Evidence from the English General Practice Patient Survey. BMJ Quality and Safety 2012.

3. Abel GA, Saunders CL, Lyratzopoulos G. Cancer patient experience, hospital performance and case mix: evidence from England. Future Oncol 2014;10:1589-98. http://dx.doi.org/10.2217/fon.13.266

4. NHS Digital. General and personal medical services, England 2002-2012 as at 30 September. 2012. URL: http://content.digital.nhs.uk/searchcatalogue?productid=10382&topics=1%2fWorkforce%2fStaff+numbers&kwd=GP+staff&geo=England&sort=Relevance&size=10&page=2#top (Accessed 31/07/2017, 2017).

5. NHS Digital. General and personal medical services, England 2003-2013 as at 30 September. 2013).

6. NHS Digital. General and personal medical services, England September 2015-March 2016, Provisional Experimental statistics. 2016. URL: http://content.digital.nhs.uk/catalogue/PUB21772 (Accessed 31/07/2017, 2017).

7. Fletcher E, Abel GA, Anderson R, Richards SH, Salisbury C, Dean SG, Sansom A, Warren FC, Campbell JL. Quitting patient care and career break intentions among general practitioners in South West England: findings of a census survey of general practitioners. BMJ Open 2017;7:e015853

8. Public Health England. National General Practice Profile. 2013. URL: http://fingertips.phe.org.uk/profile/general-practice (Accessed August 2104, 2014).

For peer review only

9. Hobbs FDR, Bankhead C, Mukhtar T. Clinical workload in UK primary care: a retrospective analysis of 100 million consultations in England, 2007-14. Lancet 2016;387:2292-.

10. Office for National Statistics (ONS). Subnational population projections for England: 2014-based projections. 2014. URL: https://www.ons.gov.uk/peoplepopulationandcommunity/populationandmigration/populationprojections/bulletins/subnationalpopulationprojectionsforengland/2014basedprojections (Accessed 31/07/2014, 2017).

11. Office for National Statistics (ONS). Quality and Methodology Information - Subnational Population Projection; 2016.

12. HSCIC. General Practice trends in the UK to 2014. Version 1.; 2015. URL: http://content.digital.nhs.uk/media/18173/General-Practice-Trends-in-the-UK-to-2014/pdf/gen-prac-trends-2014.pdf (Accessed 19/02/2017, 2017).

13. Hann M, McDonald J, Checkland K, Coleman A, Gravelle H, Sibbald B, et al. Seventh national GP worklife survey. Manchester: University of Manchester; 2013.

For peer review only

Appendix 2 – Data Flow

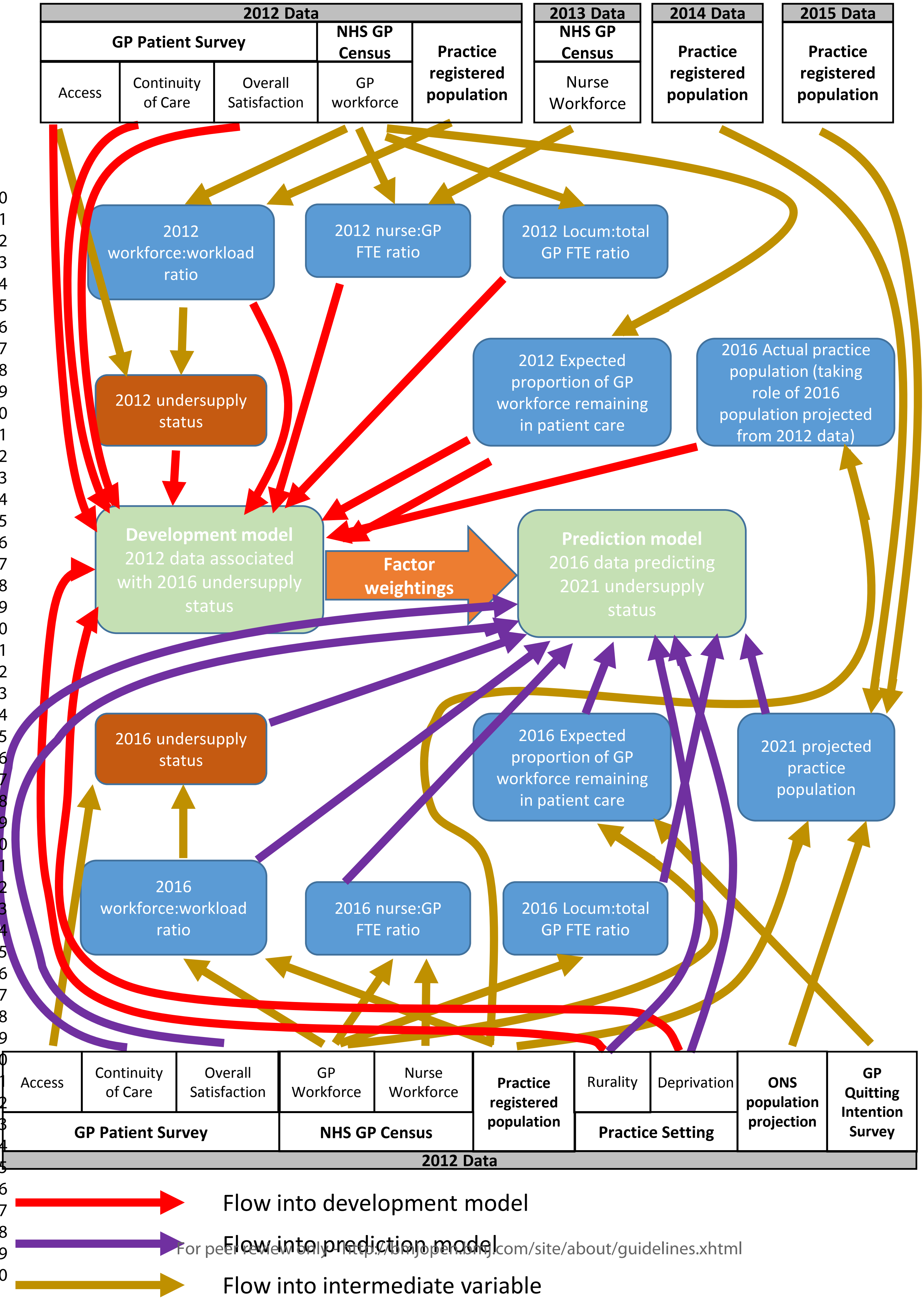

Appendix 3 - Rationale for use of interaction between ratio of total nurse FTE to total GP FTE and the expected proportion of GP FTE remaining in patient care in predictive risk model.

We recognised the need to account for the fact that GPs leaving patient care would be most likely to impact the supply–demand balance when recruitment of staff is difficult. We were unable to obtain any direct measure of the difficulty any one practice has in recruitment and so instead we explored the use of three proxy measures:

1. the use of locums (operationalised as the proportion of total GP FTE falling in the “Other” category using NHS workforce data) on the basis that practices are likely to make greater use of locums when they are struggling to recruit partners or salaried GPs;
2. patient access (using GPPS scores) on the basis that when there is a prolonged period where a practice is understaffed access may be compromised; and
3. the use of nurses (operationalised as the ratio of total nurse FTE to total GP FTE using NHS workforce data) on the basis that practices who struggle long term to recruit GPs may pass greater amounts of patient care onto nurses to maximise use of GP resource.

In exploratory analysis, an interaction between the expected proportion of the GP workforce remaining in patient care after 5 years and each of the identified proxy measures (use of locums, access, use of nurses) individually were included in the predictive model in turn. There was no evidence that either locum use or access modified the effect, in the model, of the expected proportion of the GP workforce remaining in patient care. However, there was weak evidence that the use of nurses did modify the effect of the expected proportion of the GP workforce remaining in patient care. This interaction was, therefore, retained in the final model.

For peer review only

TRIPOD Checklist: Prediction Model Development

Section/Topic	Item	Checklist Item	Page
**Title and abstract**			
Title	1	Identify the study as developing and/or validating a multivariable prediction model, the target population, and the outcome to be predicted.	1
Abstract	2	Provide a summary of objectives, study design, setting, participants, sample size, predictors, outcome, statistical analysis, results, and conclusions.	2
**Introduction**			
Background and objectives	3a	Explain the medical context (including whether diagnostic or prognostic) and rationale for developing or validating the multivariable prediction model, including references to existing models.	4
	3b	Specify the objectives, including whether the study describes the development or validation of the model or both.	4
**Methods**			
Source of data	4a	Describe the study design or source of data (e.g., randomized trial, cohort, or registry data), separately for the development and validation data sets, if applicable.	4-6
	4b	Specify the key study dates, including start of accrual; end of accrual; and, if applicable, end of follow-up.	4-9
Participants	5a	Specify key elements of the study setting (e.g., primary care, secondary care, general population) including number and location of centres.	4-6
	5b	Describe eligibility criteria for participants.	7
	5c	Give details of treatments received, if relevant.	N/A
Outcome	6a	Clearly define the outcome that is predicted by the prediction model, including how and when assessed.	7
	6b	Report any actions to blind assessment of the outcome to be predicted.	N/A
Predictors	7a	Clearly define all predictors used in developing or validating the multivariable prediction model, including how and when they were measured.	4-9
	7b	Report any actions to blind assessment of predictors for the outcome and other predictors.	N/A
Sample size	8	Explain how the study size was arrived at.	7
Missing data	9	Describe how missing data were handled (e.g., complete-case analysis, single imputation, multiple imputation) with details of any imputation method.	7
Statistical analysis methods	10a	Describe how predictors were handled in the analyses.	7-9
	10b	Specify type of model, all model-building procedures (including any predictor selection), and method for internal validation.	9
	10d	Specify all measures used to assess model performance and, if relevant, to compare multiple models.	8-9
Risk groups	11	Provide details on how risk groups were created, if done.	8
**Results**			
Participants	13a	Describe the flow of participants through the study, including the number of participants with and without the outcome and, if applicable, a summary of the follow-up time. A diagram may be helpful.	9
	13b	Describe the characteristics of the participants (basic demographics, clinical features, available predictors), including the number of participants with missing data for predictors and outcome.	9
Model development	14a	Specify the number of participants and outcome events in each analysis.	9
	14b	If done, report the unadjusted association between each candidate predictor and outcome.	N/A
Model specification	15a	Present the full prediction model to allow predictions for individuals (i.e., all regression coefficients, and model intercept or baseline survival at a given time point).	18
	15b	Explain how to the use the prediction model.	N/A
Model performance	16	Report performance measures (with CIs) for the prediction model.	11
**Discussion**			
Limitations	18	Discuss any limitations of the study (such as nonrepresentative sample, few events per predictor, missing data).	13
Interpretation	19b	Give an overall interpretation of the results, considering objectives, limitations, and results from similar studies, and other relevant evidence.	12-13
Implications	20	Discuss the potential clinical use of the model and implications for future research.	14
**Other information**			
Supplementary information	21	Provide information about the availability of supplementary resources, such as study protocol, Web calculator, and data sets.	N/A
Funding	22	Give the source of funding and the role of the funders for the present study.	3

We recommend using the TRIPOD Checklist in conjunction with the TRIPOD Explanation and Elaboration document.

For peer review only - http://bmjopen.bmj.com/site/about/guidelines.xhtml

BMJ Open

Workforce predictive risk modelling – development of a model to identify general practices at risk of a supply□demand imbalance.

Journal:	*BMJ Open*
Manuscript ID	bmjopen-2018-027934.R1
Article Type:	Original research
Date Submitted by the Author:	02-Aug-2019
Complete List of Authors:	Abel, Gary; University of Exeter, University of Exeter Medical School (Primary Care)<br>Gomez-Cano, Mayam; University of Exeter Medical School<br>Mustafee , Navonil ; University of Exeter Business School<br>Smart, Andi; University of Exeter Business School<br>Fletcher, Emily; University of Exeter Medical School, Primary Care Research Group<br>Salisbury, Chris; University of Bristol, Academic Unit of Primary Health Care<br>Chilvers, Rupa; Tangerine Bee<br>Dean, Sarah; PenCLAHRC University of Exeter Medical School,<br>Richards, Suzanne; University of Leeds, Academic Unit of Primary Care; University of Leeds, Academic Unit of Primary Care<br>Warren, F; University of Exeter,<br>Campbell, John; University of Exeter, Primary Care;
**Primary Subject Heading**:	General practice / Family practice
Secondary Subject Heading:	Health policy, Health services research
Keywords:	PRIMARY CARE, Workforce, Supply-Demand, Organisation of health services < HEALTH SERVICES ADMINISTRATION & MANAGEMENT

SCHOLARONE™
Manuscripts

Workforce predictive risk modelling – development of a model to identify general practices at risk of a supply–demand imbalance.

Gary Abel *Senior Lecturer*[1]*, Mayam Gomez-Cano *Research Associate*[1], Navonil *Mustafee Associate Professor*[2], Andi Smart *Professor of Operations & Process Management*[1], Emily Fletcher *Research Fellow*[1], Chris Salisbury *Professor of Primary Health Care*[3], Rupa Chilvers *Director of Research and Knowledge Transfer4*, Sarah *Dean Professor in Psychology Applied to Rehabilitation and Health*[1], Suzanne Richards *Professor of Primary Care Research*[,5], Fiona Warren *Senior Lecturer*[1], John Campbell *professor of general practice and primary care*[1]

[1] University of Exeter Medical School, Exeter, UK

[2] University of Exeter Business School, Exeter, UK

[3] Centre for Academic Primary Care, NIHR School for Primary Care Research, School of Social and Community Medicine, University of Bristol, Bristol, UK

[4] Tangerine Bee, Exeter, UK

[5] Academic Unit of Primary Care, Leeds Institute of Health Sciences, UK

*Corresponding author:

Dr Gary Abel
Primary Care Research Group
University of Exeter Medical School
Smeall Building, St Luke's Campus
Magdalen Road
Exeter
EX1 2LU
g.a.abel@exeter.ac.uk

Word count (excludes title page, abstract, references, figures, tables): 4745

Key Words. Primary Care, workforce, supply-demand, organisation of care

For peer review only

Objective. To develop a risk prediction model identifying general practices at risk of workforce supply–demand imbalance

Design. Secondary analysis of routine data on general practice workforce, patient experience and registered populations (2012 to 2016), combined with a census of general practitioners' career intentions (2016).

Setting/Participants. A hybrid approach was used to develop a model to predict workforce supply–demand imbalance based on practice factors using historical data (2012–2016) on all general practices in England (with over 1000 registered patients n=6,398). The model was applied to current data (2016) to explore future risk for practices in South-West England (n=368).

Primary outcome measure. The primary outcome was a practice being in a state of workforce supply–demand imbalance operationally defined as being in the lowest third nationally of access scores according to the General Practice Patient Survey and the highest third nationally according to list size per full time equivalent GP (weighted to the demographic distribution of registered patients and adjusted for deprivation)

Results. Based on historic data, the predictive model had fair to good discriminatory ability to predict which practices faced supply–demand imbalance (area under the ROC curve 0.759). Predictions using current data suggested that, on average, practices at highest risk of future supply–demand imbalance are currently characterised by having larger patient lists, employing more nurses, serving more deprived and younger populations, and haveing considerably worse patient experience ratings when compared with other practices. Incorporating findings from a survey of GPs career intentions made little difference to predictions of future supply–demand risk status when compared with expected future workforce projections based only on routinely-available data on GPs' gender and age.

Conclusions. It is possible to make reasonable predictions of an individual general practice's future risk of undersupply of general practitioner workforce with respect to its patient population. However, the predictions are inherently limited by the data available.

Strengths and limitations of this study

- This study made use of freely available data from a range of sources to develop a predictive model of workforce supply-demand imbalance for general practices in England
- Historical data for all of England is used to develop factor weightings which are then applied to current data.
- The additional value of a census survey of career intentions of GPs in south-west England is explored comparing findings to predictions made on the basis of general practice workforce age and Gender alone.
- The predictive model is inherently limited by the data available, and in particular we note that routine data of a measure of a practices difficulty in recruiting staff were not available.

Data sharing statement. Most data used in this study are publicly available from referenced sources. Data from the GP Census survey can be made available on request from the corresponding author of the original publication at john.campbell@exeter.ac.uk.

Funding. The project was funded by the National Institute for Health Research, Health Service and Delivery Research programme (project 14/196/02). The views and opinions expressed herein are those of the authors and do not necessarily reflect those of the Health Service and Delivery Research programme, the National Institute for Health Research, the National Health Service, or the Department of Health.

Competing interests. All authors have completed the ICMJE uniform disclosure form at www.icmje.org/coi_disclosure.pdf (available on request from the corresponding author) and declare: grants from the National Institute for Health Research (Health Service and Delivery Research programme) during the conduct of the study. SD's position is partly supported by the National Institute for Health Research (NIHR) Collaboration for Leadership in Applied Health Research and Care South West Peninsula at the Royal Devon and Exeter NHS Foundation Trust. The views expressed are those of the author(s) and not necessarily those of the NHS, the NIHR or the Department of Health.

Ethics approval. Ethics approval for the GP Census survey was provided by the University of Exeter Medical School Research Ethics Committee. All other data was publically available and so ethical approval was not required for its use.

For peer review only

Introduction

Against a backdrop of 34,495 full time equivalent (FTE) General Practitioners in 2016, the NHS in England saw a reduction of 3.5% of the English general practitioner (GP) workforce (1193 FTE) in a single year.[1] This reduction has been seen in combination with rising demands of the patient population.[2] Such figures represent a 'crisis' in respect of GP workforce capacity, with particular problems in retaining established GPs in direct patient care [3 4]. Similar problems in respect of family doctor recruitment and retention are evident in other western healthcare economies and jurisdictions [5 6], and many countries have explored what might constitute optimal skill mix amongst primary care health professionals over the last 40 years [7-9].

There is, however, a need for the rational deployment of the GP workforce resource.[10 11] Various models exist to inform that deployment, with GP workload representing a key issue amongst individual GPs electing to quit patient care [3]. Gaining an understanding of GP workload pressures is also the basis of identifying any potential mismatch between the demand for general practice services, and the supply of GPs to meet that demand. In many countries, the general practice represents a key element in the delivery of primary care and acts as the basis for general practice workforce planning. For example, practices are the basis of reporting of patients' experience of primary care in England, captured using the General Practice Patient Survey [12].

The aim of this research was to develop a method to identify NHS general practices in one region of England which may face supply–demand workforce imbalances within the next 5 years. Previous workforce modelling in the UK has focussed upon deriving insights from analyses at the regional or national (macro) level [13]. In contrast, the research we are reporting here focuses on undertaking predictive risk modelling at a practice (micro) level. Routine workforce modelling makes use of data on doctors' age and gender, and historical retirement patterns. Here we consider whether surveying GPs' career intentions adds value to such modelling.

Methods

Overview

The first step in developing a predictive model to identify general practices at risk of future supply–demand imbalance is to define what is meant by a supply–demand imbalance and to operationalise this with measurable quantities. Assessing the supply of GP workforce at any one general practice is reasonably straightforward, however assessing the demand of patients is complex as unmet demand is, by its nature, hard to quantify. Instead here we consider the expected workload given the demographics of the patient population served. The balance between supply and demand within this framework is then represented by the expected workload per practitioner. However, high workload alone may not be an issue.

For peer review only

Practices with high workload may meet patient demand through innovative and efficient systems of service delivery. High workload is considered to have a negative impact only when service delivery is impaired. For the purposes of this study we defined those practices with high workload per practitioner in combination with an inability to meet patient demand as being in a state of 'under-supply'. Here we use the term 'under-supply' to indicate a practice which has a high demand from patients for a given supply of doctors which appears to be having a detrimental impact on services [14]. In this study we used a measure of patient access as a proxy for the ability to meet patient demand, in the belief that access is an important measure, reflecting the ease with which patients might engage with the primary healthcare system [14 15].

Several data sources have been brought together in this work. Analyses were performed at general practice level, firstly, to identify practices which were currently in 'under-supply' and, secondly, to identify those which are likely to have such problems in future. A predictive risk model (to predict the risk of a practice being in a state of 'under-supply' within 5 years) was developed by assessing the associations between current (2016) 'under-supply' status and historical routinely collected data (where available) on GP workforce, practice characteristics (rurality, deprivation, population) patient experience scores from 2012. The model further incorporated projected future populations in each area and considered projected future GP workforce based on GPs stated career intentions (from a survey of GPs). The rationale for this approach was to obtain factor weightings informed by evidence developed on past data. This model was then used to identify practices and areas in South West England that are likely to experience a supply–demand imbalance ('under-supply') in the future.

Data sources

Except where specified, national data for England were obtained and processed. A summary of data sources is given below with full details given in Appendix 1 along with a schematic illustrating the data flow used in the modelling process (Appendix 2).

General Practice Patient Survey (GPPS)

The GPPS is a national postal survey of patients' experience of primary care in England distributed to around 2.8 million adult patients each year [12]. We used data from the 2011/12 and 2015/16 surveys, during which the contents of the survey remained largely consistent. Response rates were 38% in 2011/12 (1,037,946 responses) and 39% in 2015/16 (836,312 responses) with an average of around 125 respondents per practice. We used three GPPS items GPPS reflecting access ("Last time you wanted to see or speak to a GP or nurse from your GP surgery: Were you able to get an appointment to see or speak to someone?"), continuity of care ("How often do you see or speak to the GP you prefer?"), and overall experience ("Overall, how would you describe your experience of your GP surgery?").

For peer review only

Workforce

Workforce data at practice level were obtained from NHS Digital and related to GP Census data taken as at 30 September 2012, 2013 and 2016 [16-18]. Total GP FTE and GP FTE in the "other" category were extracted for 2012 and 2016 (where "other" is assumed to mostly be locum GPs given that registrars, salaried GPs, and those on retainer schemes, are captured in specific categories). Total nurse FTE was extracted for 2013 and 2016 (nurse data were not available in 2012, so 2013 data were used in its place).

General practitioner Quitting intentions

Self-reported GP intentions to cease practice were collected through a census survey which has been reported elsewhere [19]. Briefly, a questionnaire was administered to all active GPs in South West England in April-June 2016, enquiring about their intentions to cease/interrupt practice within 2 and 5 years (3370 questionnaires sent, 2248 returned, response rate 67%). We made use of responses to three questions:

- *"How likely is it that you will permanently leave direct patient care within the next 5 years?"*
- *"How likely is it that you will take a career break (or another career break) within the next 5 years?"*
- *"In your current/most recent direct patient role, how many sessions do/did you work in a typical week?"*

The first two questions had response options of "Very Likely", "Likely", "Unlikely" and "Very unlikely" whereas the latter had a free text response.

Practice rurality and deprivation

Practice rurality (rural/urban) based on an Office for National Statistics (ONS) categorisation of the postcode of the practice was obtained, as was a practice deprivation score based on the 2015 Index of Multiple Deprivation (IMD) [20].

Practice registered population

Data on the registered populations for each general practice were obtained for each quarter from April 2014 to April 2016 (9 datasets); as well as April 2012. These datasets provided the count of patients of each gender (male, female) by 5-year age-band strata.

Subnational population projections

We made use of the Office for National Statistics (ONS) subnational population projections developed to inform the local planning of healthcare and other public services for geographically defined populations served by Clinical Commissioning Groups (CCGs, organisations responsible for commissioning NHS services) [21]. These projections are demographic, trend-based projections that indicate the 'likely levels of future population' and are routinely produced every 2 years. We extracted projected populations for 2021 for the eight CCGs within South West England. Projections were made in 5-year age-bands for each gender.

For peer review only

Data preparation and variable creation

Brief details are given below with full details in Appendix 1

Patient experience

Case-mix adjusted practice scores for patient experience were created following previous methodology [22 23] adjusting for patient age, gender, ethnicity, presence of a long-term condition, and deprivation, using mixed effects logistic regression. The case-mix adjusted scores were based on dichotomous outcomes and used in the form of log-odds ratios relative to the average practice nationally.

Workforce

Practices with less than 0.5 full-time equivalent (FTE) GPs (38 out of 7,484 practices in 2012 data and 41 out of 6,709 practices in 2016 data) were excluded from all analyses on the basis that such a low staff record indicated either that these were unusual practices or that the workforce data were in error. In addition to total GP FTE, the ratio of nurse FTE to doctor FTE and the ratio of doctor FTE in the "other" category to total doctor FTE were calculated.

Workload

We used a definition of workload based on registered patients rather than on recorded patient visits. Patient visits are a measure of actual work undertaken which is limited by the workforce available, and so cannot capture unmet demand. By focussing on the registered population, we estimated the expected workload to serve that population based on national averages. Weights were applied to patient list sizes in order to standardise for the age and gender composition of the practice population, accounting for the fact that GPs spend longer, on average, consulting with patients who are very young, are older, or are female [2]. Further adjustment was made for the deprivation of the practice population to reflect higher health needs. These adjusted weighted list sizes were divided by the total GP FTE to obtain a measure of workload per GP FTE. Initial inspection of the workload figures showed that the distribution contained some infeasibly large and small values. Practices in the top and bottom 2.5% of the distribution were excluded from all further analysis. This exclusion took place following the removal of practices with less than 0.5 GP FTE.

Expected remaining future workforce

We estimated the proportion of GP FTE that would be expected, on average, to remain in patient care in 5 years' time. We did this in two principal ways: (i) using information on the age and gender of GPs at the practice along with previous work which identified the probability that GPs of different ages and genders leave patient care [24]and (ii) based on responses to survey of GP career intentions. The former was done for both 2012 and 2016 data and the latter only for 2016 data. The approaches are detailed in full in Appendix 1.

Outcome definition

Ability to meet patient demand was quantified using the GPPS access measure (ability to make an appointment), reflecting the ease with which patients might engage with the

For peer review only

primary healthcare system. Workload to workforce ratio was quantified using the workload per GP FTE quantity described above. Practices that were in the lowest third of GPPS access scores and also in the highest third of workload per GP FTE nationally were defined as being in 'under-supply' (i.e. demand exceeded supply). Having used relative measures and cut points which were defined pragmatically for the purposes of this study in our definition of undersupply, we do not propose absolute and objective measures about whether a practice is 'failing' to deliver care. Indeed if provision of care were good everywhere and the supply of workforce were not an issue, such an approach would be inappropriate. However, in the current climate in the UK, this represents a pragmatic approach in the absence of a direct measure.

Development of predictive risk model

Historical data were used to produce model coefficients which could then be applied to current data. Model development was based on all available national data in order to maximise statistical power .We did not split the data into development and validation samples as changes over time in healthcare delivery are more likely to be a threat to future use of the model than over-fitting. Predictor variables were based on 2012 data unless otherwise noted and included

- three GPPS scores
- adjusted weighted list size per GP FTE (workforce to workload ratio)
- total GP FTE
- the ratio of "other" GP FTE to total GP FTE
- the expected proportion of GP FTE still in patient care in 2017
- ratio of nurse FTE to doctor FTE (using nurse FTE data from 2013)
- 2016 adjusted weighted list size (using 2016 data)
- rurality setting (based on 2016 data, but not expected to change)
- practice deprivation (based on 2016 data, but not expected to change)

We did not attempt to predict the 2016 practice populations using only data available in 2012 and instead included the observed 2016 practice populations as an additional explanatory variable due to a lack of data available for 3 years prior to 2012.

A logistic regression model was used with a binary outcome of a practice being in a state of under-supply in 2016 based on 2016 data (see outcome definition above). Practices were the unit of analysis. All variables considered were included and retained regardless of statistical significance.

We recognised the need to account for the fact that GPs leaving patient care would be most likely to impact the supply–demand balance when recruitment of staff was difficult. We were unable to obtain any direct measure of the difficulty any one practice had in recruitment and so instead we explored the use of three proxy measures:

For peer review only

1. The use of locums (operationalised as the proportion of total GP FTE falling in the "Other" category using NHS workforce data), on the basis that practices are likely to make greater use of locums when they are struggling to recruit partners or salaried GPs;

2. Patient access (using GPPS scores), on the basis that when there is a prolonged period where a practice is understaffed access may be compromised; and

3. The use of nurses (operationalised as the ratio of total nurse FTE to total GP FTE using NHS workforce data), on the basis that practices which have difficulty in recruiting GPs may employ more nurses to take on aspects of patient care traditionally delivered by GPs freeing up GP time.

In exploratory analysis, an interaction between the expected proportion of the GP workforce remaining in patient care after 5 years and each of the identified proxy measures (use of locums, access, use of nurses) individually were included in the predictive model in turn. There was no evidence that either locum use or access modified the effect, in the model, of the expected proportion of the GP workforce remaining in patient care. However, there was weak evidence that the use of nurses did modify the effect of the expected proportion of the GP workforce remaining in patient care. This interaction was, therefore, retained in the final model.The predictive value of our model was assessed using a ROC (Receiver Operating Characteristic) curve analysis of predicted probabilities for all practices in England based on the data used to build the model (i.e. 2012 data and 2016 supply–demand imbalance classifications). These were compared with a simpler model developed using only two explanatory variables which were 2012 data for factors defining the under-supply (GPPS access scores and adjusted weighted list size per FTE, noting that the outcome of the model, under-supply was still based on 2016 data). Calibration was assessed by comparing the mean predicted probability from the main model and the percentage of practices in undersupply in 2016 for deciles of predicted probability. We also performed a sensitivity analysis to examine the impact of excluding the top and bottom 2.5% of practices in terms of workload per GP FTE. To do so we re-ran the logistic regression after excluding only the top and bottom 1% of practices in terms of workload per GP FTE.

Future risk prediction

The coefficients from the historical model were applied to 2016 data to form our baseline risk predictions with a 5-year forward view for practices in South West England only. The reason for the restriction to those practices was that they were the only ones for which we had survey responses on future career intentions). It should be noted that although the original outcome definition was a relative one, the model treated them as absolute. In other words predictions obtained from the model identify the risk of having a workload to workforce ratio in 2021 higher than two-thirds of practices did in 2016 and a GPPS access score in 2021 lower than two-thirds of practices did in 2016. In the context of a nationally worsening situation this would allow for considerably more practices to be in a state of undersupply. Practices in the highest 25% of the predicted risk profile were flagged as "high risk" of future under-supply of GP workforce, those in the lowest 25% were flagged as being "low risk", and those in between were flagged as being at "moderate risk".

For peer review only

The usefulness of the career intention survey was examined by comparing the above prediction with an alternative prediction using the expected proportion of the GP workforce remaining in patient care in five years' time based only on the routinely available age and gender profile of GPs in the practice.

In addition to baseline predictions, we explored a number of `stress testing' scenarios. These scenarios can be considered as stress tests of the model to identify practices that might be more (or less) vulnerable to particular challenges. First, we explored the effect of increased difficulty in recruiting GPs, which we modelled as an increase in the coefficient for the expected proportion of GPs remaining in patient care (where an increased coefficient implies a greater impact of GP workforce leaving patient care). Second, we explored which practices might be at particular risk of a marked increase in local population. This was done by inflating the predicted adjusted weighted list size. The following scenarios were explored:

A. The coefficient for expected proportion of GPs remaining in patient care increased by 2 (equivalent to a 22% increase in the odds of being in supply–demand imbalance when 10% of GPs are expected to leave representing a modest increase in the difficulty of recruiting GPs);
B. The coefficient for expected proportion of GPs remaining in patient care increased by 4 (equivalent to a 49% increase in the odds of being in supply–demand imbalance when 10% of GPs are expected to leave representing a substantial increase in the difficulty of recruiting GPs);
C. The predicted adjusted weighted list size increased by 20%;
D. The predicted adjusted weighted list size increased by 40%;
E. A modest increase in difficulty recruiting GPs combined with a 20% increase in list size (a and c combined); and
F. A substantial increase in difficulty recruiting GPs combined with a 40% increase in list size (b and d combined).

For each of these scenarios, practices were rated according to relative risk (i.e. top 25% were labelled "high relative risk" as above) and absolute risk. The relative risk cut-offs in the baseline scenario were used for absolute risk cut-offs in the other scenarios.

Patient and public involvement

This study was part of a wider programme of work considering GP workforce issues which was served by a Patient and Public Involvement (PPI) group which provided input to the overall design and conduct of the research. Developing methods and results were shared atproject management group meetings, which included PPI representatives who directly contributed to refining methods and interpreting and contextualising the results.

For peer review only

Results

Mapping the current situation

A total of 6,398 practices in England had available data on all data items and had list sizes>1000; 371 of these were in South West England. The distribution of practices in England as a whole and South West England is shown in Figure 1. Practices with GPPS access scores (ability to make an appointment - our proxy for ability to meet patient demand) in the highest scoring third nationally were over-represented in South West England, with 57% of practices in this region falling in that category. There was also an under-representation of South West practices nationally in respect of workload (only 22% of practices in the region were classified as in the third of practices nationally with the highest workload). As a result, the percentage of practices defined as currently being in under-supply was considerably lower in South West England (5.1%) than in England as a whole (13.5%).

There was no evidence that list size varied between those practices in under-supply and other practices in South West England (Table 1). However, there was evidence that practices in under-supply had fewer FTE GPs. Together, these findings indicate that observed differences in workload are driven more by the supply of GP workforce than the demand of the registered patient population. Practices in undersupply also had a higher ratio of nurse FTE to GP FTE, served more deprived populations, had lower patient experience scores, had fewer patients over the age of 65, and were more likely to be in urban areas.

Predictive risk model

The regression coefficients for the logistic model are shown in Table 2 Predictive risk model coefficients estimated using 2012 data where possible to estimate the independent association with 2016 undersupply status. A negative coefficient implies a reduced risk of future undersupply as the value of the variable increases when all other variables are kept constant. We note the interaction between the expected proportion of GP FTE still working in patient care in 5-years' time and the ratio of nurse FTE to doctor FTE had a relatively large p-value (0.177). In initial modelling (before excluding practices on the basis of data quality) this interaction variable had a smaller p-value (0.06) indicating some evidence that it was worth including. When exclusions were applied, the coefficient did not change meaningfully. This fact, combined with the *a-priori* expectation that the effect of expected future GP workforce would be dependent on recruitment, provided support to maintain the interaction term. The sensitivity analysis excluding only the top and bottom 1% of practices in terms of workload per GP FTE produced broadly similar regression coefficients with the exception of the coefficient for the expected proportion of GP workforce to remain in patient care which was reduced by 43% (results not shown).

Figure 1 Distribution of practices in England and in south-west England across categories according to workforce to workload ratio and GPPS access scores.

For peer review only

Figure 2 ROC curve for the predictive risk model based on the national historical data used to build the model.

shows the ROC curve derived from the development model (i.e. 2012 covariates and 2016 outcome). The area under the curve was 0.759. The ROC curve from the simpler model only including the defining factors (GPPS access scores and adjusted weighted list size per FTE) had an area under the curve of 0.718, suggesting that the additional variables included in our model provided a modest, but meaningful, improvement in predictive value. A visual inspection of a calibration plot for the full model suggests that there is good calibration of the model (Appendix 3).

Future risk predictions

Applying the risk prediction model to data from 2016, seeking to predict the risk of future supply–demand imbalance for individual practices in South West England, we obtained risk scores for 368 practices with available data remaining after applying exclusions. The median probability of future supply–demand imbalance across practices was 5.4% (IQR 2.8% to 10.0%). In total 40 (10.9%) practices had a risk greater than 20%, and 12 (3.3%) had a risk greater than 50%. Table 3 shows the characteristics of those practices in South West England classified as high risk (top 25% of practices, corresponding to an absolute risk of 10% or greater) of being in a state of under-supply compared with other practices. In contrast to the current situation shown in Table 2, there was no evidence (p=0.445) that the total GP FTE varies between high/other risk classification. There was evidence, however, that all other descriptive factors varied between the two groups. Practices at "high risk" of future supply–demand imbalance tended to currently have larger list sizes, to have a higher proportion of nurses in the workforce, to serve more deprived and younger populations, have considerably worse GPPS scores, and were more likely to be in urban areas.

Stress testing scenarios

Figures 2 and 3 illustrate the changes to the relative and absolute risk of future under-supply under different stress testing scenarios. In this figure, each practice is represented by a horizontal bar. The vertical ordering of each practice is the same in each scenario, and is based on the rank ordering of each practice according to the baseline risk prediction. For each scenario, the colouring of every practice's horizontal bar illustrates the relative or absolute risk classification (Figure 3 and 4 respectively) such that changes in colour indicate changes in risk classification. In Figure 3 practices coloured red (high risk) are in the top 25% of practices in terms of risk of undersupply for any given scenario, practices coloured green (low risk) are in the bottom 25% for any given scenario, with the middle 50% of practices coloured yellow. In Figure 4 practices coloured red (high risk) have an absolute risk of future undersupply greater than 10% (corresponding to the minimum absolute risk of future undersupply of the top 25% of practices in the baseline scenario), practices coloured green

For peer review only

(low risk) have an absolute risk less than 2.8% (corresponding to the maximum absolute risk of the bottom 25% of practices in the baseline scenario) and intermediate practices are coloured yellow.

Comparing the baseline prediction (where responses to the career intention survey were used to predict the future GP workforce remaining in patient care), with a prediction using only GP age and gender, very little difference was observed in practices categorised as being either at “high relative risk” or “high absolute risk” of undersupply (seen in Figure as limited reclassification of practices, correlation of ranks=0.999).

In general, practices classified as being at “high relative risk” remained so under scenario A (modest increase in the difficulty of GP recruitment to replace those leaving - correlation in ranks between scenario a and baseline=0.97). However, there was a dramatic increase in the number of practices with a predicted absolute risk of future undersupply greater than 10% (seen as an increase in the number of practices coloured red Figure , scenario A). There was an even greater disturbance in the classification of practices under scenario B (illustrating the recruitment of GPs was becoming much harder), though the reclassification in terms of relative risk was still relatively modest (Figure , scenario B, correlation in ranks between scenario B and baseline=0.90). Conversely, the reclassification in terms of absolute risk (Figure 4, scenario B) was significantly greater; the majority of practices had a predicted risk above 10%.

Increasing the projected practice population resulted in only modest changes in respect of which practices are classified as being at “high relative risk”. Only a small relative increase was seen when comparing scenarios C and D with the baseline predictions (Figure correlation in ranks between scenario C and baseline=0.99 and scenario D and baseline=0.98). However, substantial changes were seen in the number of practices with an absolute risk of undersupply greater than 10% (Figure 4, scenarios C and D). Combining the effect of scenarios A and C resulted in relative risk classifications closer to the baseline predictions than scenario A alone. However, in terms of absolute risk, more practices had a risk greater than 10% (Figure 4, scenario A and scenario C).

When scenario B and scenario d were combined (illustrating a situation where it was much harder to recruit GPs combined with an increased practice population of 40%) it was evident that nearly all practices (88%) exceeded 10% absolute risk of supply–demand imbalance within 5 years, with only 9 (2.4%) practices classified as being at “low absolute risk” using the cut-offs derived from the baseline predictions.

Discussion

Summary of main findings

We developed a predictive risk model informed by historical data which could be used to predict an individual practice’s future risk of being in a state of GP workforce undersupply.

For peer review only

The model produced a range of risk scores attributable to practices across South West England, and has a fair to good discriminatory ability in this context (based on the ROC curve analysis). Applying this to current data suggests that the practices at highest risk of future undersupply of GP workforce have, on average: larger patient lists; employ more nurses relative to doctors; serve more deprived and younger populations; and have considerably worse patient experience ratings.

We modelled scenarios where the recruitment of GPs becomes harder and/or practice populations increase dramatically beyond what would be expected from historical trends (for example, through a new housing development). These scenarios do increase absolute risk dramatically, but by and large, it is the same practices in all scenarios that are at highest risk of future undersupply of GP workforce. This almost certainly reflects the fact that those most likely to have problems in the future are those currently experiencing difficulties. This can be seen in the relatively good predictions from a simple model including only defining factors (i.e. workload per FTE GP and GPPS patient access scores) which had an area under the ROC curve which was not substantially less than the full model. In particular, we found that inclusion of findings from our own survey of GPs' career intentions had very little impact on the predictions, compared with using expected future workforce projections based only on routinely available data regarding GPs' gender and age.

Strengths and weaknesses

Strengths of this work include the comprehensive use of freely available data as well as the exploratory use of a census survey of career intentions of GPs in the region. The main strength is the novel development of factor weightings based on routinely available historical data. However, we recognise that this assumes that factors driving changes are constant from the historical time period of model development to the future time period of prediction. This is unlikely to be the case given recent problems in GP workforce recruitment and retention in the UK [4]. To this end we have modelled what might be expected if recruitment was harder than it has been historically, and if there were substantive increases in the practice population. These scenarios may be more reflective of what we might expect going forward.

The main weakness of this work concerns our ability to distinguish in what situations, and in which practices, future GP workforce leaving patient care will impact the level of continuing GP workforce and its ability to meet patient demand. For practices that do not encounter problems in recruiting GPs, retiring GPs pose much less of an issue than for practices where recruitment is difficult. Here we relied on the level of nurse staffing in a practice as a proxy for recruitment issues; importantly, this means the association of more nurses with at-risk practice status is likely to be attributable to practices being unable to fill GP vacancies, not that more nurses per se puts a practice at risk. A more direct measure of recruitment problems which was consistently and widely collected (such as duration of advertising for vacant posts, using a consistent methodology to track this) would be expected to provide a better model. Unfortunately, no robust freely available measure exists. The NHS GP census does collect data on time to fill vacancies [18] and existing unfilled vacancies. However, these

For peer review only

data are not freely available, and, furthermore, are not mandatory for completion by practices.

Another weakness was that historical workforce data were not available in the same detail as current data (including nurse data not being available for 2012 at all). This meant that future workforce predictions using historical data would not be as accurate as those using current data. These inaccuracies would lead to a loss of power, and potentially an attenuation of the associated regression coefficients. This may explain the low statistical significance of associated coefficients in the model.

Finally, we note that our assessment of the performance of our model was made on the same data the model was developed on, and thus is likely to overestimate the performance of the model. Validation of the future risk predictions would be welcome, but can only be undertaken in 5 years' time.

Implications

We have demonstrated that it is possible to make reasonable predictions of an individual general practice's future risk of undersupply of GP workforce with respect to its patient population. With ongoing GP workforce issues in the UK, local models are being developed to identify potentially "at-risk" practices [25]. However, unlike the model we present here, it is not clear to what extent these models are evidence-based or to what extent their limitations are recognised by the users of the models or even what is meant by "at risk".

Whilst the model we present here is set in the context of UK primary care, the general approach could be applied to other settings and in other locations. In all cases the predictions will be inherently limited by the quality of available data. Improvements in data quality going forward will help the situation in the UK, particularly if data are released on GP recruitment. However, it will be some time before robust historical data exist that can be used for the model development process outlined here. If models such as the one outlined here are to be produced and used, it is important that high-quality data continue to be collected. The predictions produced by this model and similar models may facilitate targeting of interventions to retain and attract GP workforce either in specific practices, or in specific regions currently at high risk of problems driven by workforce supply. Although our model provides reasonable discrimination, much could potentially be achieved by focussing efforts on those practices currently experiencing difficulties.

Whilst a policy of targeted interventions may have value, we find that most practices are likely to be at a high risk of workforce undersupply when faced with a substantial increase in demand from an increased patient population combined with major difficulties in recruiting GPs. As such, local knowledge of drivers of increased practice populations, such as housing developments, will be key to being able to suitably apply targeted interventions. Even in South West England where workload and the ability to meet patient demand are better than in England overall, most practices are currently vulnerable to recruitment challenges, and will remain so going forward. Given this, national or broad regional policies and strategies may be more effective than targeted ones, especially if there is limited knowledge on how local populations are likely to evolve.

For peer review only

Contributions. GA, JC, AS and NM conceived the study. GA, MGC and NM performed analysis. GA, MGC, NM, AS, EF, CS, RC, SD, SR, FW and JC contributed to the design and interpretation of the study. GA drafted the initial manuscript. GA, MGC, NM, AS, EF, CS, RC, SD, SR, FW and JC commented and critically reviewed the manuscript prior to submission..

For peer review only

1. BMJ. Seven days in medicine: 1-7 February 2017. *BMJ* 2017;356 doi: 10.1136/bmj.j668
2. Hobbs FR, Bankhead C, Mukhtar T, et al. Clinical workload in UK primary care: a retrospective analysis of 100 million consultations in England, 2007–14. *The Lancet* 2016;387(10035):2323-30.
3. Sansom A, Terry R, Fletcher E, et al. Why do GPs leave direct patient care and what might help to retain them? A qualitative study of GPs in South West England. *BMJ Open* 2018;8(1) doi: 10.1136/bmjopen-2017-019849
4. Roland M, Everington S. Tackling the crisis in general practice. *BMJ* 2016;352 doi: 10.1136/bmj.i942
5. Kash BA, Ogden P, Popp E, et al. Exploring future models of primary care for Texas. *International Journal of Innovation Science* 2017;9(4):435-54. doi: doi:10.1108/IJIS-01-2017-0005
6. Miani C, Pitchforth E, Ellen N. Choice of primary care provider: a review of experiences in three countries: Policy Research Unit in Policy Innovation Research (PIRU), 2013.
7. Spitzer WO, Sackett DL, Sibley JC, et al. The Burlington randomized trial of the nurse practitioner. *The New England journal of medicine* 1974;290(5):251-6. doi: 10.1056/nejm197401312900506 [published Online First: 1974/01/31]
8. Freund T, Everett C, Griffiths P, et al. Skill mix, roles and remuneration in the primary care workforce: who are the healthcare professionals in the primary care teams across the world? *International journal of nursing studies* 2015;52(3):727-43. doi: 10.1016/j.ijnurstu.2014.11.014 [published Online First: 2015/01/13]
9. Laurant M, Reeves D, Hermens R, et al. Substitution of doctors by nurses in primary care. *The Cochrane database of systematic reviews* 2005(2):Cd001271. doi: 10.1002/14651858.CD001271.pub2 [published Online First: 2005/04/23]
10. Paddison C, Abel G, Campbell J. GPs: working harder than ever. *The British journal of general practice : the journal of the Royal College of General Practitioners* 2018;68(670):218-19. doi: 10.3399/bjgp18X695873 [published Online First: 2018/04/28]
11. Royal College of General Practitioners. Fit for the Future: a Vision for general practice, 2019.
12. NHS England. GP Patient Survey: NHS England; [Available from: https://gp-patient.co.uk/surveys-and-reports accessed 05/03/2018.
13. The Centre for Workforce Intelligence. In-depth review of the general practitioner workforce, 2014.
14. Campbell JL, Salisbury C. Research into practice: accessing primary care. *Br J Gen Pract* 2015;65(641):e864-e68. doi: 10.3399/bjgp15X688057
15. Campbell J, Smith P, Nissen S, et al. The GP Patient Survey for use in primary care in the National Health Service in the UK – development and psychometric characteristics. *BMC Fam Pract* 2009;10(57):Doi: 10.1186/471-2296-10-57.
16. NHS Digital. General and personal medical services, England 2002-2012 as at 30 September 2012 [Available from: http://content.digital.nhs.uk/searchcatalogue?productid=10382&topics=1%2fWorkforce%2fStaff+numbers&kwd=GP+staff&geo=England&sort=Relevance&size=10&page=2#top accessed 31/07/2017 2017.
17. NHS Digital. General and personal medical services, England 2003-2013 as at 30 September 2013 [
18. NHS Digital. General and personal medical services, England September 2015-March 2016, Provisional Experimental statistics 2016 [Available from: http://content.digital.nhs.uk/catalogue/PUB21772 accessed 31/07/2017 2017.
19. Fletcher E, Abel GA, Anderson R, et al. Quitting patient care and career break intentions among general practitioners in South West England: findings of a census survey of general practitioners. *BMJ open* 2017;7(4) doi: 10.1136/bmjopen-2017-015853

For peer review only

20. Public Health England. National General Practice Profile 2013 [Available from: http://fingertips.phe.org.uk/profile/general-practice accessed August 2104 2014.
21. Office for National Statistics. Subnational population projections for England: 2014-based projections 2014 [Available from: https://www.ons.gov.uk/peoplepopulationandcommunity/populationandmigration/populationprojections/bulletins/subnationalpopulationprojectionsforengland/2014basedprojections accessed 31/07/2014 2017.
22. Paddison C, Elliott M, Parker R, et al. Should measures of patient experience in primary care be adjusted for case mix? Evidence from the English General Practice Patient Survey. *BMJ Qual Saf* 2012
23. Abel GA, Saunders CL, Lyratzopoulos G. Cancer patient experience, hospital performance and case mix: evidence from England. *Future oncology (London, England)* 2014;10(9):1589-98. doi: 10.2217/fon.13.266 [published Online First: 2013/12/18]
24. Centre for Workforce Intelligence. In-depth review of the general practitioner workforce - Final report 2014.
25. Healthy London Partnership. Primary Care (GP) Workforce Model 2016 [Available from: http://healthylondon.org/hlp-archive/sites/default/files/The%20Primary%20Care%20Model%20Step-by-Step%20Guide.pdf accessed 05/03/18.

For peer review only

Table 1 Comparison of practices in South West England defined as in undersupply with other practices in the region.

	Under-supply (n=19)			Other (n=352)			
	**median**	**25%**	**75%**	**median**	**25%**	**75%**	**p value***
List size	9264	5361	11576	7598	5270	11077	0.448
Adjusted weighted list size	8959	5212	12287	8099	5638	11570	0.550
GP FTE	3.1	2	5.1	4.7	3.2	6.6	0.012
Ratio nurse/GP FTE	0.8	0.7	1	0.5	0.4	0.7	<0.001
Index of Multiple Deprivation	25.7	20.2	30.9	18.7	13.5	24.4	0.003
GPPS access‡	0.2	0.1	0.2	0.7	0.5	0.9	<0.001
GPPS continuity‡	0.2	0.2	0.3	0.6	0.4	0.8	<0.001
GPPS satisfaction‡	0.2	0.1	0.4	0.7	0.5	0.9	<0.001
% over 65	16.8	13.3	21	22.6	17.6	26	0.004
**Setting**	**n**	**%**		**n**	**%**		**p value†**
Urban practices	17	6.8		232	93.2		0.042
Rural practices	2	1.6		120	98.4		

*** from Mann–Whitney test**
† from Fisher's exact test
‡GPPS scores used were case-mix adjusted log-odds ratios relative to the average practice nationally
** IMD scores are given (rather than ranks) with higher scores indicating higher levels of deprivation.

For peer review only

Table 2 Predictive risk model coefficients estimated using 2012 data where possible to estimate the independent association with 2016 undersupply status.

Data type	Variable	Note on units	Logistic regression coefficient (95% CI)	p-value
GP Patient Survey Scores*	Access	Random effect (log-odds ratio) from logistic case-mix adjustment model	-0.96 (-1.21, -0.70)	<0.001
	Continuity of care		-0.09 (-0.25, 0.07)	0.274
	Overall Satisfaction		-0.48 (-0.70, -0.27)	<0.001
Baseline Workforce†	Ratio of nurse FTE to GP FTE		1.02 (-0.05, 2.09)	0.062
	Adjusted Weighted List Size per GP FTE	Per 1000 patients per GP FTE	0.40 (0.18, 0.62)	<0.001
	Total GP FTE		-0.17 (-0.25, -0.10)	<0.001
	Ratio of “Other” GP FTE to total GP FTE		0.65 (0.32, 0.98)	<0.001
Rurality Setting‡	Urban practice		Reference	0.404
	Rural practice		-0.13 (-0.43, 0.17)	
Index of Multiple Deprivation – practice in quintile‡	1 – least deprived		Reference	<0.001
	2		0.02 (-0.29, 0.32)	
	3		0.13 (-0.16, 0.42)	
	4		0.57 (0.29, 0.85)	
	5 – most deprived		0.36 (0.06, 0.66)	
Projected quantities	Adjusted Weighted List Size**	Per 1000 patients	0.14 (0.10, 0.18)	<0.001
	Proportion of GP FTE still in patient care*	Varies from 0 to 1	0.38 (-0.78, 1.54)	0.520
	Proportion of GP FTE still in patient care x Ratio of nurse FTE to GP FTE*		-1.01 (-2.48, 0.46)	0.177
Constant			-4.15 (-5.10, -3.21)	<0.001

*Data from 2012
†Data from 2012 except nurse data which were from 2013
‡IMD data from 2016 for variable where this status is expected to remain relatively constant over time
**Actual list size from 2016 rather than projected list size based on 2012 data as pre-2012 data did not allow projections comparable to those which were made with more current data looking forwards.

For peer review only

For peer review only - http://bmjopen.bmj.com/site/about/guidelines.xhtml

Table 3 Differences between practices identified at high risk of future undersupply and other practices assuming a baseline scenario.

	High risk (n=92)			Other (n=276)			
	**median**	**25%**	**75%**	**median**	**25%**	**75%**	**p value***
List size	10625	7732	13195	6915	4941	10206	<0.001
Adjusted weighted list size	11133	7369	13252	7398	5251	10615	<0.001
GP FTE	5	3.1	6.6	4.5	3.1	6.6	0.445
Ratio of nurse FTE to GP FTE	0.7	0.5	1	0.4	0.4	0.6	<0.001
IMD	25.6	18.7	31.7	17.6	13.1	22.2	<0.001
GPPS access‡	0.4	0.2	0.6	0.8	0.6	0.9	<0.001
GPPS continuity‡	0.3	0.2	0.5	0.7	0.5	0.9	<0.001
GPPS satisfaction‡	0.4	0.2	0.6	0.7	0.5	0.9	<0.001
% over 65	18.3	14.1	23.4	23.2	18.5	26.5	<0.001
Setting	**n**	**%**		**n**	**%**		**p value†**
Urban practices	77	31.3		169	68.7		<0.001
Rural practices	15	12.3		107	87.7		

*** from Mann–Whitney test**
† from Fisher's exact test
‡GPPS scores used were case-mix adjusted log-odds ratios relative to the average practice nationally

For peer review only

Figure 1 Distribution of practices in England and in south-west England across categories according to workforce to workload ratio and GPPS access scores.

For peer review only

Figure 2 ROC curve for the predictive risk model based on the national historical data used to build the model.

For peer review only

*Risk prediction as for baseline, but using age and gender of GPs alone rather than including responses to the career intentions survey. In each case the practices are ordered by the baseline scenario.

Figure 3 Rating of practices in South West England from different risk prediction scenarios a-d using cut-offs defined by the quartiles of each prediction (relative risk).

For peer review only

*Risk prediction as for baseline, but using age and gender of GPs alone rather than including responses to the career intentions survey. In each case the practices are ordered by the baseline scenario.

Figure 4 Rating of practices in South West England from different risk prediction scenarios a-d using cut-offs defined by the quartiles of the baseline prediction (absolute risk).

For peer review only

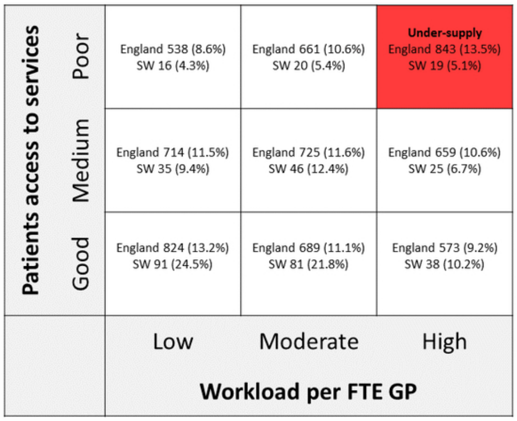

Figure 1 Distribution of practices in England and in south-west England across categories according to workforce to workload ratio and GPPS access scores.

90x50mm (300 x 300 DPI)

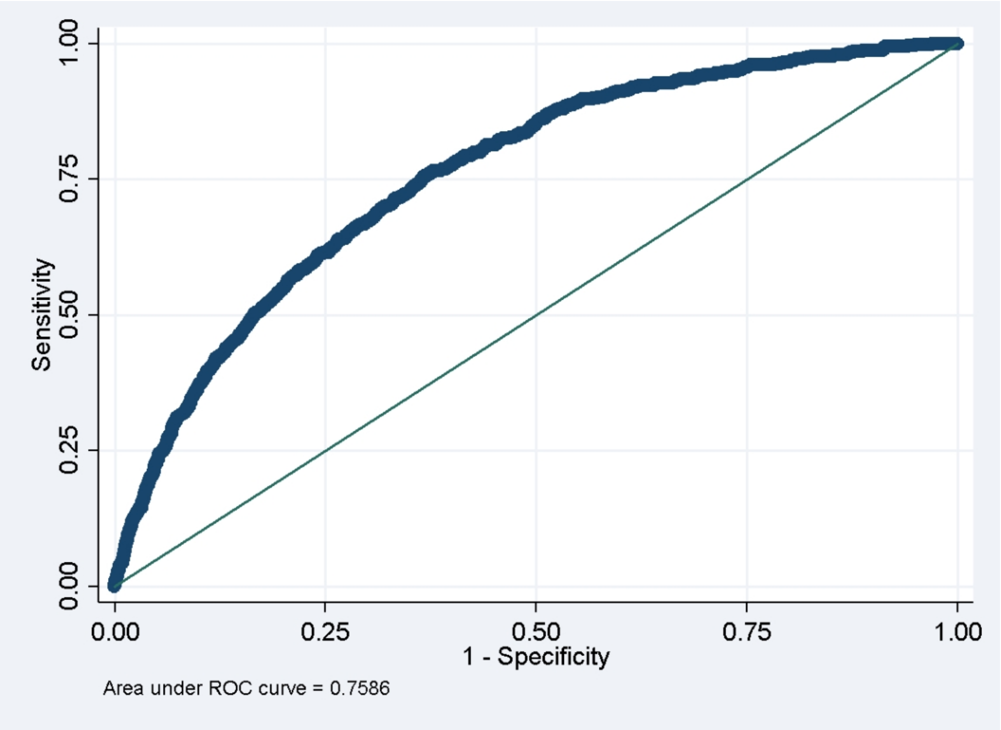

Figure 2 ROC curve for the predictive risk model based on the national historical data used to build the model.

101x73mm (300 x 300 DPI)

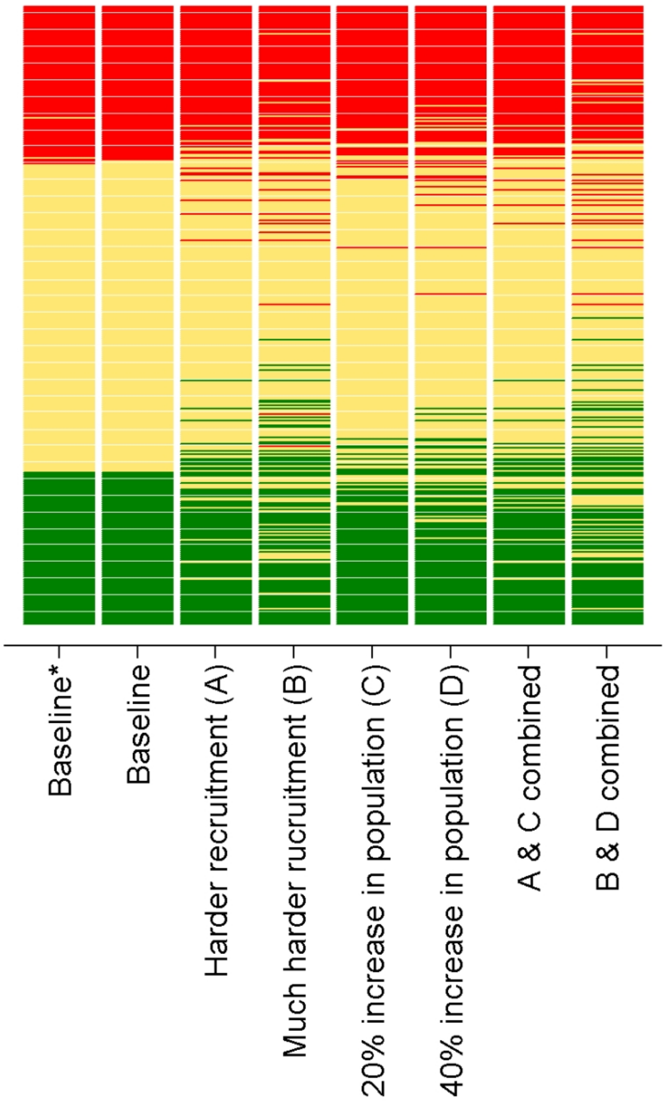

Figure 3. Rating of practices in South West England from different risk prediction scenarios a-d using cut-offs defined by the quartiles of each prediction (relative risk).

95x127mm (300 x 300 DPI)

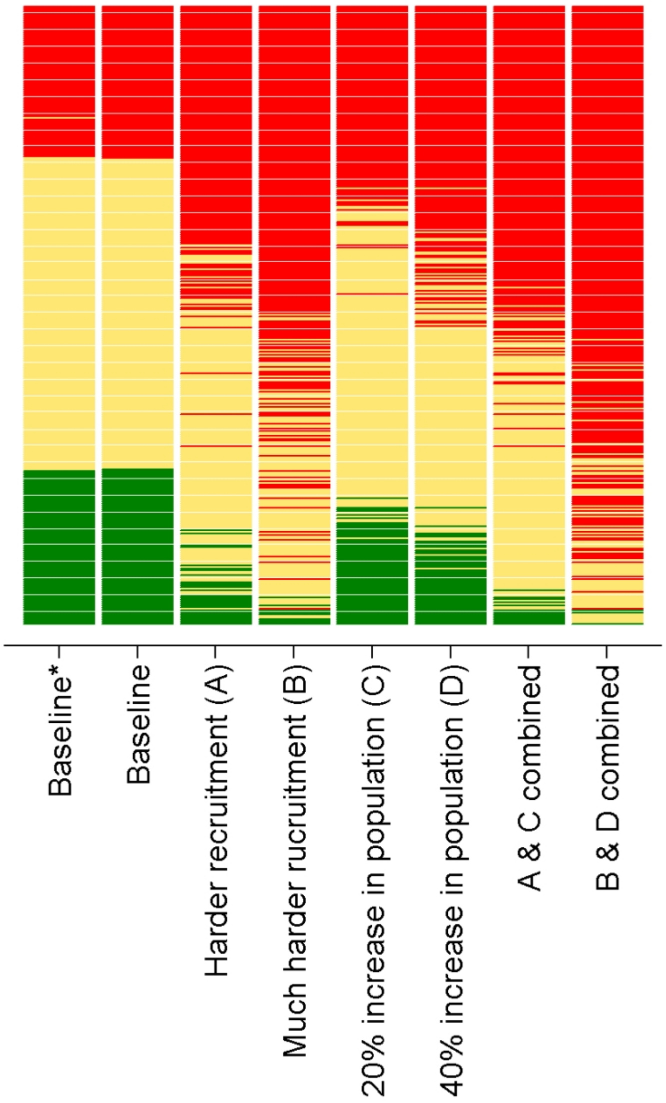

Figure 4 Rating of practices in South West England from different risk prediction scenarios a-d using cut-offs defined by the quartiles of the baseline prediction (absolute risk).

95x127mm (300 x 300 DPI)

Appendix 1 - Data sources and preparation

Except where specified, national data for England were obtained and processed. A summary of data sources and data flow used in the modelling process is presented in Appendix 2.

GP Patient Survey

The General Practice Patient Survey (GPPS) is a national postal survey of patients' experience of primary care in England. Patients from practices that are known from prior surveys to have low response rates are oversampled. Full details of the sampling strategy are published elsewhere.[1] We used data from the 2011/12 and 2015/16 surveys. The contents of the survey have remained largely consistent over this time period. Response rates were 38% in 2011/12 (1,037,946 responses) and 39% in 2015/16 (836,312 responses).

We made use of three items from the GPPS reflecting access, continuity of care and overall experience. For patient's experience of access the following question was used:

- *"Last time you wanted to see or speak to a GP or nurse from your GP surgery:" "Were you able to get an appointment to see or speak to someone?"*

Responses of "Yes" and "Yes, but I had to call back closer to or on the day I wanted the appointment" were coded as a positive response and responses of "No" were coded as a negative response. Responses of "Can't remember" were treated as uninformative and excluded from the analysis.

The item on ability to see a preferred doctor is taken as a proxy measure for continuity of care:

- *"How often do you see or speak to the GP you prefer?"*

Responses of "Always or almost always" and "A lot of the time" were coded as a positive response and responses of "Some of the time" and "Never or almost never" were coded as a negative response. Responses of "Not tried at this GP surgery" were treated as uninformative and excluded from the analysis.

Finally, an item capturing data on the patient's overall experience of care is included:

- *"Overall, how would you describe your experience of your GP surgery?"*

Responses of "Very good" and "Fairly good" were coded as a positive response and responses of "Neither good nor poor", "Fairly poor" and "Very poor" were coded as a negative response. There were no uninformative options for this question.

Due to certain patient groups tending to give more positive responses in patient surveys, case-mix adjusted practice scores were created. This was achieved using mixed effects logistic regression adjusting for patient age, gender, and ethnicity, presence of a long-term condition, and deprivation

For peer review only

(using the Index of Multiple Deprivation [IMD], an area based measure assigned according to the patient's residential postcode[2 3]) and a random intercept for practice. The case-mix adjustment provides scores for individual practices based on a standardised mix of patients. The case-mix adjusted scores were used in the form of log-odds ratios relative to the average practice nationally.

Workforce

Workforce data were obtained from NHS Digital and related to the GP Census data taken as at 30 September 2012, 2013 and 2016.[4-6] Each dataset gave the headcount of GPs in 5-year age-bands for each practice. The 2012 dataset contained total GP headcount by gender as did the 2016 dataset. In the 2016 dataset additional detail of GP FTE by gender was provided. Both datasets contained total GP FTE as well as GP FTE broken down by GP role. We also extracted the total nurse FTE from the 2016 dataset. As nurse FTE data were not available in 2012, the relevant data were extracted from the 2013 dataset in its place. From these data two further variables were derived: the ratio of nurse FTE to GP FTE; the ratio "other" category FTE to total GP FTE (where "other" is assumed to mostly be Locum GPs given that GP registrars, salaried GPs, and those on retainer schemes are captured in other categories). These data were also used in the derivation of workload and the predicted remaining future workforce.

Practices with less than 0.5 FTE GP (38 out of 7,484 practices in 2012 data and 41 out of 6,709 practices in 2016 data) were excluded from all analyses on the basis that such a low staff record indicated either that these were unusual practices or that the workforce data were in error. In the former case such unusual practices are not the focus of this work and in the latter case, erroneous inferences may have been made if they had been included.

GP quitting intentions

To predict remaining future workforce we utilised self-reported GP intentions to cease practice collected through a survey which formed part of the ReGROUP project and has been reported.[7] Briefly, a questionnaire was administered to all active GPs in South West England in April-June 2016, enquiring about their intentions to cease/interrupt practice in the next 2 and 5 years (3370 questionnaires sent, 2248 returned, response rate 67%). We combined responses to two questions:

- *"How likely is it that you will permanently leave direct patient care within the next 5 years?"*
- *"How likely is it that you will take a career break (or another career break) within the next 5 years?"*

Each question had response options of "Very Likely", "Likely", "Unlikely" and "Very unlikely". Where GPs gave different response options for these two questions, the response with the highest likelihood

For peer review only - http://bmjopen.bmj.com/site/about/guidelines.xhtml

of cessation or interruption was taken. This reflects the most likely chance of impact to future GP workforce in the next 5 years. We also used respondents' answers to the question:

- *"In your current/most recent direct patient role, how many sessions do/did you work in a typical week?"*

Free text responses to this question provided data from which an estimate of each responder's current FTE work commitment could be calculated. Working eight sessions per week was taken as 1 FTE, consistent with the approach used in the GP census.[6] When more than eight sessions was given as a response the FTE was capped at 1. If more than 24 sessions was given as a response it was assumed the question had been answered incorrectly and the data were treated as missing. Data for all GPs surveyed on age, gender and affiliated practice were obtained from the Performers List.

Practice rurality and deprivation

Practice rurality was contained within the GPPS 2016 dataset and was based on an Office for National Statistics (ONS) categorisation of the postcode of the practice. We used a rural/urban version of this categorisation. Practice deprivation score was obtained from Public Health England and was based on the 2015 IMD. Individual patient IMD is based on each patient's residential postcode, and the practice score is the mean of individual patient scores using all patients registered at the practice.[8]

Practice registered population

Data on the registered populations for each general practice were obtained from NHS Digital for each quarter from April 2014 to April 2016 (9 datasets); as well as April 2012. These datasets provided the count of patients in each gender by 5-year age-band (with the highest age-band being 95 and over). We aggregated the top three age-bands resulting in a top age-band category of 80+ years.

The April 2012 and April 2016 datasets were used to calculate list sizes weighted for the demographics of the populations and adjusted for deprivation. The reason for weighting for patient demographics is that certain types of patients (older, female and very young) place a higher demand on practices than others. The adjustment for deprivation acknowledges that deprived populations have higher health needs than less deprived populations with a similar demographic profile. To calculate weighted list sizes the practice populations were weighted according to the average time spent consulting with patients in 14 age by gender groups in 2013/14 according to a recent study based on routine patient records from 674 practices.[9] Weighted lists sizes ($\boldsymbol{P_W}$) were then normalised so the total population across the country remained unchanged. These weighted list sizes are taken as a measure of workload on the basis that they represent a measure of the expected time spent consulting. This assumes that, on average, patients in the same demographic group require the same amount of consultation time. Because age and gender do not capture the health status of the population the weighted list sizes

For peer review only

were then adjusted for deprivation (IMD decile, IMD_i, taking a value between 1 and 10, based on all practices in England) assigning a 10% weighting to a deprived population. The adjusted weighted population will thus be given by

$$P_{AW} = 0.9P_W + 0.1\left(\frac{P_W IMD_i}{\sum P_W IMD_i}\sum P_W\right) \quad 1$$

This approach is intended to mirror that used in the current resource allocation to CCGs. However, the CCG allocations do not use deprivation, but rather make use of a measure of premature mortality (the <75 standardised mortality ratio, which is the ratio of mortality in under 75 year olds to that expected given the age and sex composition of the CCG population). We chose to use deprivation here as standardised mortality ratios are not published for individual practices.

The adjusted weighted list sizes were divided by the total GP FTE to obtain a measure of workload per GP. Initial inspection of the workload figures showed that the distribution contained some infeasibly large and small values. These may have arisen from errors in either the workforce or practice population data. Unfortunately, there was no clear separation between typical values and those that were infeasible. A pragmatic approach was taken whereby practices in the top and bottom 2.5% of the distribution were excluded from all further analysis. This exclusion took place following the removal of practices with less than 0.5 GP FTE.

The data from April 2014 to April 2016 were used in the prediction of future practice populations along with the subnational population projections described below.

Subnational population projections

We made use of ONS subnational population projections at the level of CCGs (used to inform local planning of healthcare and other public services[10 1110 11]) in the prediction of future practice populations (see below). The subnational ONS projections are demographic, trend-based projections that indicate the 'likely levels of future population' and are currently produced every 2 years; they present projections for every year for the next 25 years from the base year.[12] The underlying data sources that inform the calculations include: national population projections; registration of births and deaths (General Registrar Office); armed forces data (MOD); data extracts from the Patient Register Data System (NHS); student location data (Higher Education Statistics Agency [HESA]); and data on asylum seekers (Home Office). Adjustments were then made to the datasets for factors such as assumed fertility and mortality rates, internal and international migration. However, the projections do not account for local development aims and policies, economic factors, and indeed any international factors that are likely to affect the UK population.[10] We extracted projected populations

For peer review only

for 2021 for the eight CCGs within the scope of the ReGROUP project: NHS Bath and North East Somerset CCG; NHS Kernow CCG; NHS North, East, West Devon CCG; NHS South Devon and Torbay CCG; NHS Bristol CCG; NHS North Somerset CCG; NHS Somerset CCG; NHS South Gloucestershire CCG. Projections are made in 5-year age-bands for each gender. As with practice population data the upper age groups were combined to form an 80+ age-band.

Projecting future workload

Our projections of future practice workloads were based on the number of patients registered at each of the 423 GP practices in South West England, in 5-year age bands, split by gender combined with subnational population projections from the ONS as described above. The approach comprises the following five steps.

1. Assess congruency of ONS predictions with list size
   ONS subnational population projections were compared with GP list size data aggregated to CCG level for 2014, 2015 and 2016. This provided an assessment of the degree to which ONS predications reflect the actual GP list size data in those years. This difference between the two data sources is most likely due to "list inflation", caused by patients that have not been removed from the list following death, dual registrations for patients when moving homes or by a registered patient's failure to complete the national census.[9] Given that the average consultation times used to weight the populations (described above) are based on registered patients, we did not consider it appropriate to resize practice list sizes to reflect the identified difference.
2. Calculate the proportion of CCG population registered at each GP practice
   For each practice, and for each age-band by gender stratum, we identified the number of patients registered with the practice and the expected number of patients within a CCG for nine time-points between April 2014 to April 2016. This allowed us to derive the proportion of the total CCG population by gender/age-interval registered at each practice. If the number of practices in a CCG is declining over time we might expect the proportion of the CCG population to be rising at the remaining practices.
3. Quantify trends in the proportion of the CCG population registered at each general practice
   The data from step 2 were used as the outcome variable in a logistic regression model that included a linear term for time as well as a categorical variable for quarter to quantify trends. A separate regression model was used for each practice by age-band by gender strata.
4. Determine projected count of patients
   We used the resultant regression equation to predict the proportion of CCG patients by practice/gender/age-interval for five years beyond the final data point. Multiplying this

proportion by the ONS predicted population for the same time point gives a projected count of patients.

5. Project adjusted list size

   The projected populations were used to create a projected adjusted weighted list size using the same algorithm used above for observed populations.

Predicting remaining future workforce

When predicting future workforce (supply) we concentrated on predicting what fraction of the existing workforce will remain available to the practice in 5 years' time. We did this in two principal ways: i) based on the age and gender of GPs at the practice; ii) based on responses to the ReGROUP survey of GP quitting intentions. Predictions are made based on 2012 data and 2016 data (with the survey only being available for the 2016 data).

Approach 1 – Using the age and gender profile of GPs at each practice.

Previous work has identified the probability that GPs of a given single year of age and gender will remain in the workforce 12 months later.[13] By multiplying these probabilities over five consecutive single year age bands we obtain the probability that GPs of a given single year of age and gender will remain in the workforce in 5 years' time. As the routinely available GP census data (p.**Error! Bookmark not defined.**) is only available in five-year age-bands, we take the mean of these 5-year probabilities over the 5-year age-bands used in the GP census data. Unfortunately, the GP census data published at practice level gives data by either age or gender, but not both. Furthermore, data by age is only given in terms of headcount, as is data by gender in 2012 (data by gender is given in terms of headcount and FTE in 2016). Thus we adopted the following procedure to estimate remaining workforce.

1. Using the probabilities described above, identify the probability that each GP in the practice will remain in patient care in 5 years' time based on their age-band assuming they are male.
2. Calculate the mean of these probabilities over all GPs in the practice.
3. Repeat steps 1 and 2 assuming they are female.
4. Take a weighted average of the probabilities obtained in steps 2 and 3 weighted by the FTE of male and female GPs in the practice (in 2012 data headcount by gender is used instead).

For peer review only

The resulting probabilities can then be interpreted as the proportion of GP FTE which is expected, on average, to remain at the practice in 5 years' time.

Approach 2 – Using the ReGROUP survey responses.

An alternative approach used in the forecasting utilised the results of the ReGROUP survey where all GPs in South West England were asked about their future career intentions. For GPs who responded to the survey (67%) we used both stated career intentions, stated FTE (as described above), and information on age and gender. For non-responders we simply used age and gender information (provided within the Performers List). To incorporate the survey responses we made use of odds ratios estimated from a previous study which linked stated quit intentions to working status 5 years later and adjusted for age and gender.[14] Odds ratios for their 5-point scale are mapped to our 4-point scale by ignoring the middle (neutral) option.

1. It proved difficult to map the ReGROUP survey responses to the NHS GP census data (due to inconsistent age, gender and FTE information between the two data sources). Therefore, in this methodology, the GP census data are only used in the estimation of FTE of survey non-responders based on difference between the total GP FTE (GP census data) and the total FTE stated by responders linked to each practice within the Performers List. This was done using the following method. We calculated the difference between the total GP FTE given in the GP census data and the stated total GP FTE of responders to the survey linked through the Performers List to each practice in the study. The assumed FTE of non-responders was this difference divided by the number of non-responders linked to the practice. Where this difference was greater than the number of FTEs, the non-responders were assigned an FTE of 1. Where this difference was negative, non-responders were assigned an FTE of 0.

2. We then calculated probabilities of remaining in patient care for the forthcoming 5 years. For the survey non-responders, we assigned a probability of remaining in patient care using the same method as in approach 1 but based on the individual GP's gender and current year of age taken from the Performers List (rather than the GP census). For responders, we similarly assigned a probability of remaining in patient care based on the individual GP's age and gender and then adjusted that probability using the following odds ratios (Calculated from Hann et al. [14] but changing the baseline to the neutral category) "Very likely" 1.94, "Likely" 1.3, "Unlikely" 0.70, and "Very unlikely" 0.43.

3. For each practice, we then took the weighted average of the probabilities obtained in step 2 (over GPs associated with a practice, weighted according to their FTE.

The resulting probabilities can then be interpreted as the proportion of GP FTE that would be expected, on average, to remain at the practice in 5 years' time.

References

1. NHS England. GP Patient Survey: NHS England; [Available from: https://gp-patient.co.uk/surveys-and-reports accessed 05/03/2018.
2. Paddison C, Elliott M, Parker R, et al. Should measures of patient experience in primary care be adjusted for case mix? Evidence from the English General Practice Patient Survey. *BMJ Qual Saf* 2012
3. Abel GA, Saunders CL, Lyratzopoulos G. Cancer patient experience, hospital performance and case mix: evidence from England. *Future Oncol* 2014;10(9):1589-98. doi: 10.2217/fon.13.266
4. NHS Digital. General and personal medical services, England 2002-2012 as at 30 September 2012 [Available from: http://content.digital.nhs.uk/searchcatalogue?productid=10382&topics=1%2fWorkforce%2fStaff+numbers&kwd=GP+staff&geo=England&sort=Relevance&size=10&page=2#top accessed 31/07/2017 2017.
5. NHS Digital. General and personal medical services, England 2003-2013 as at 30 September 2013 [
6. NHS Digital. General and personal medical services, England September 2015-March 2016, Provisional Experimental statistics 2016 [Available from: http://content.digital.nhs.uk/catalogue/PUB21772 accessed 31/07/2017 2017.
7. Fletcher E, Abel GA, Anderson R, et al. Quitting patient care and career break intentions among general practitioners in South West England: findings of a census survey of general practitioners. *BMJ Open* 2017;7(4):11. doi: 10.1136/bmjopen-2017-015853
8. Public Health England. National General Practice Profile 2013 [Available from: http://fingertips.phe.org.uk/profile/general-practice accessed August 2104 2014.
9. Hobbs FR, Bankhead C, Mukhtar T, et al. Clinical workload in UK primary care: a retrospective analysis of 100 million consultations in England, 2007–14. *The Lancet* 2016;387(10035):2323-30.
10. Office for National Statistics (ONS). Subnational population projections for England: 2014-based projections 2014 [Available from: https://www.ons.gov.uk/peoplepopulationandcommunity/populationandmigration/populationprojections/bulletins/subnationalpopulationprojectionsforengland/2014basedprojections accessed 31/07/2014 2017.
11. Office for National Statistics. Subnational population projections for England: 2014-based projections 2014 [Available from: https://www.ons.gov.uk/peoplepopulationandcommunity/populationandmigration/populationprojections/bulletins/subnationalpopulationprojectionsforengland/2014basedprojections accessed 31/07/2014 2017.
12. Office for National Statistics (ONS). Quality and Methodology Information - Subnational Population Projection. 2016

For peer review only

13. Centre for Workforce Intelligence. In-depth review of the general practitioner workforce: final report. London, 2014.

14. Hann M, Reeves D, Sibbald B. Relationships between job satisfaction, intentions to leave family practice and actually leaving among family physicians in England. *European journal of public health* 2011;21(4):499-503. doi: 10.1093/eurpub/ckq005 [published Online First: 2010/02/10]

For peer review only

Appendix 2a – Data Flow Main development model

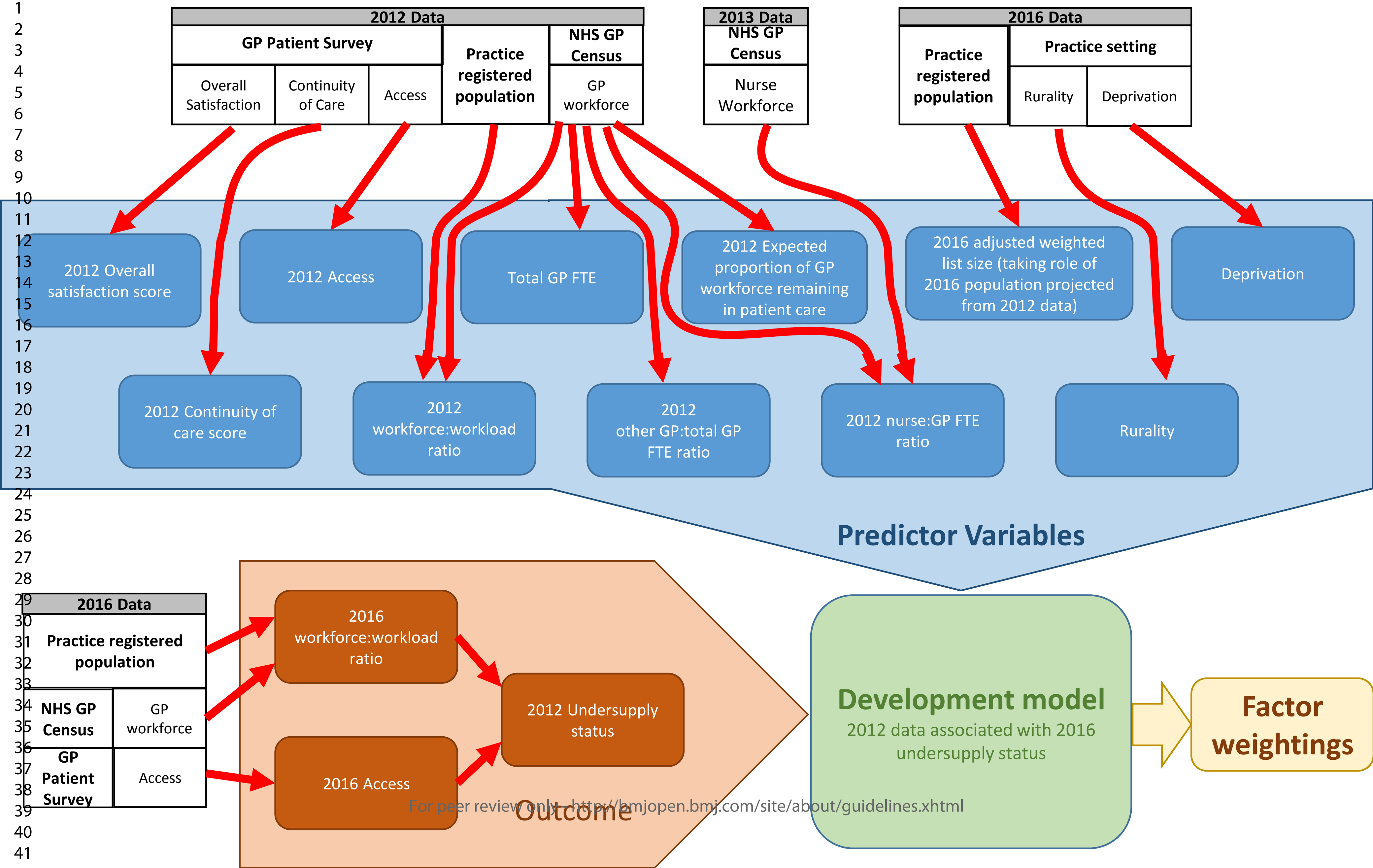

For peer review only - http://bmjopen.bmj.com/site/about/guidelines.xhtml

Appendix 2b – Data Flow Main prediction model

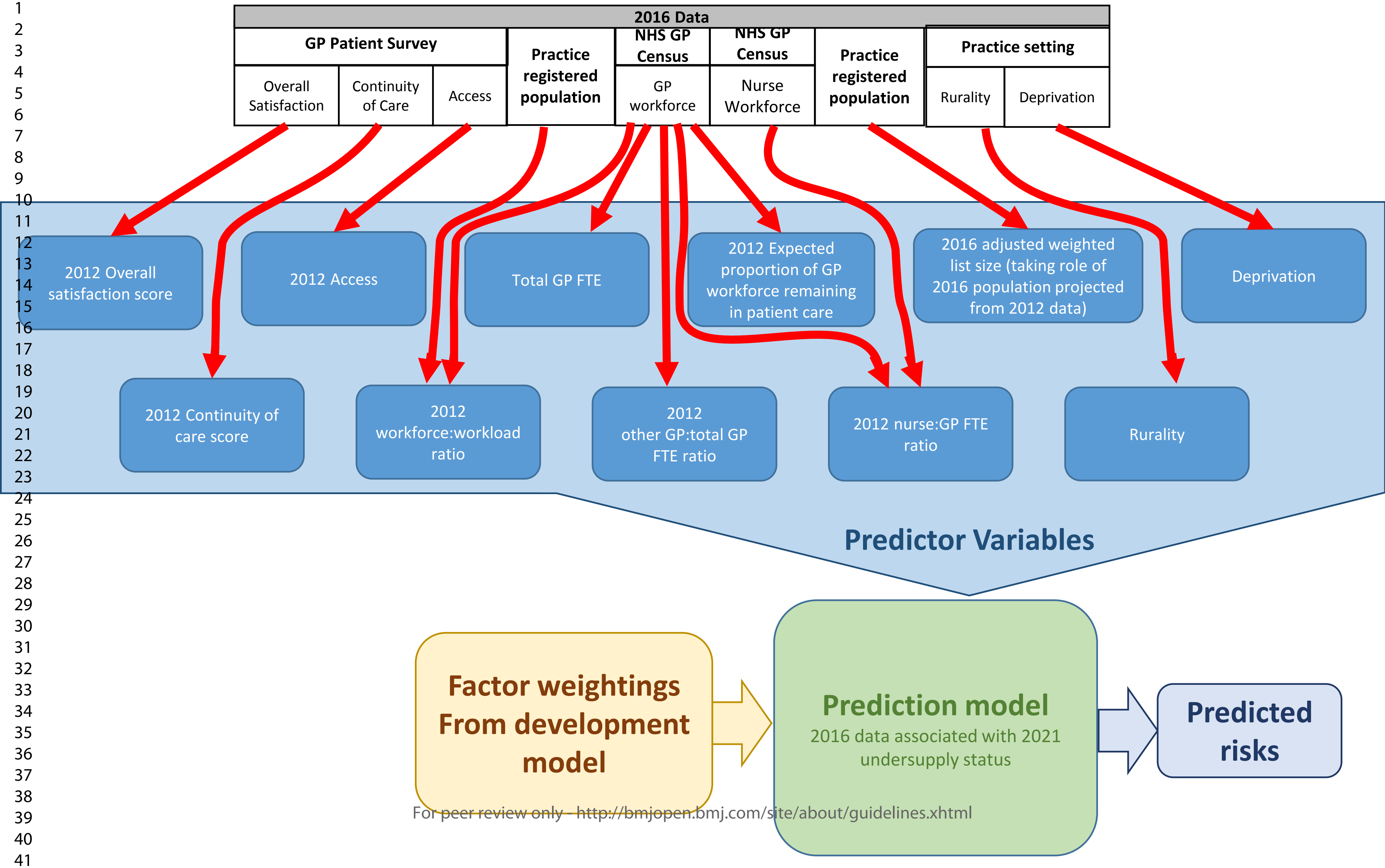

Appendix 2c – Data Flow Simpler development model

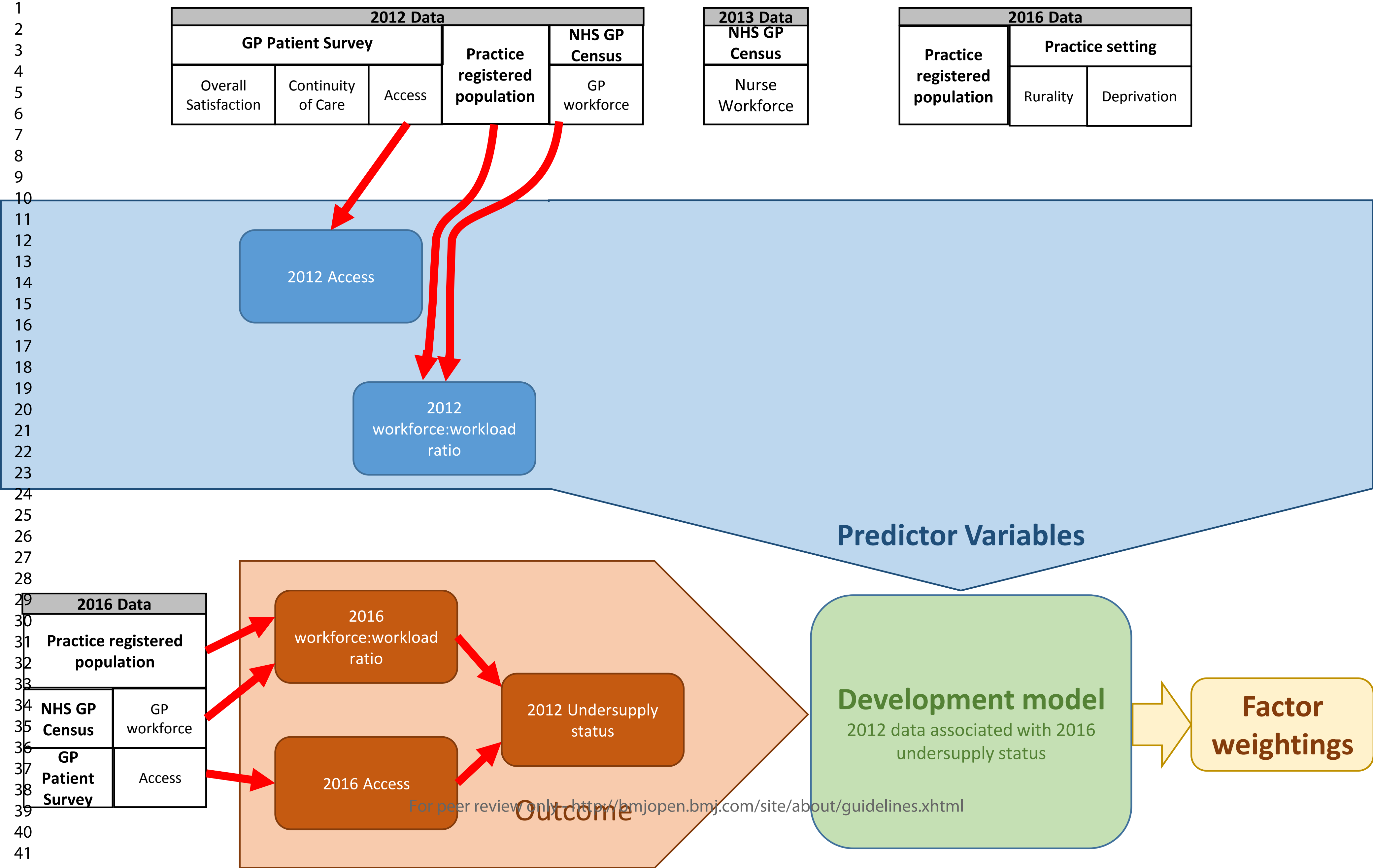

For peer review only - http://bmjopen.bmj.com/site/about/guidelines.xhtml

Appendix 2d – Data Flow Simpler prediction model

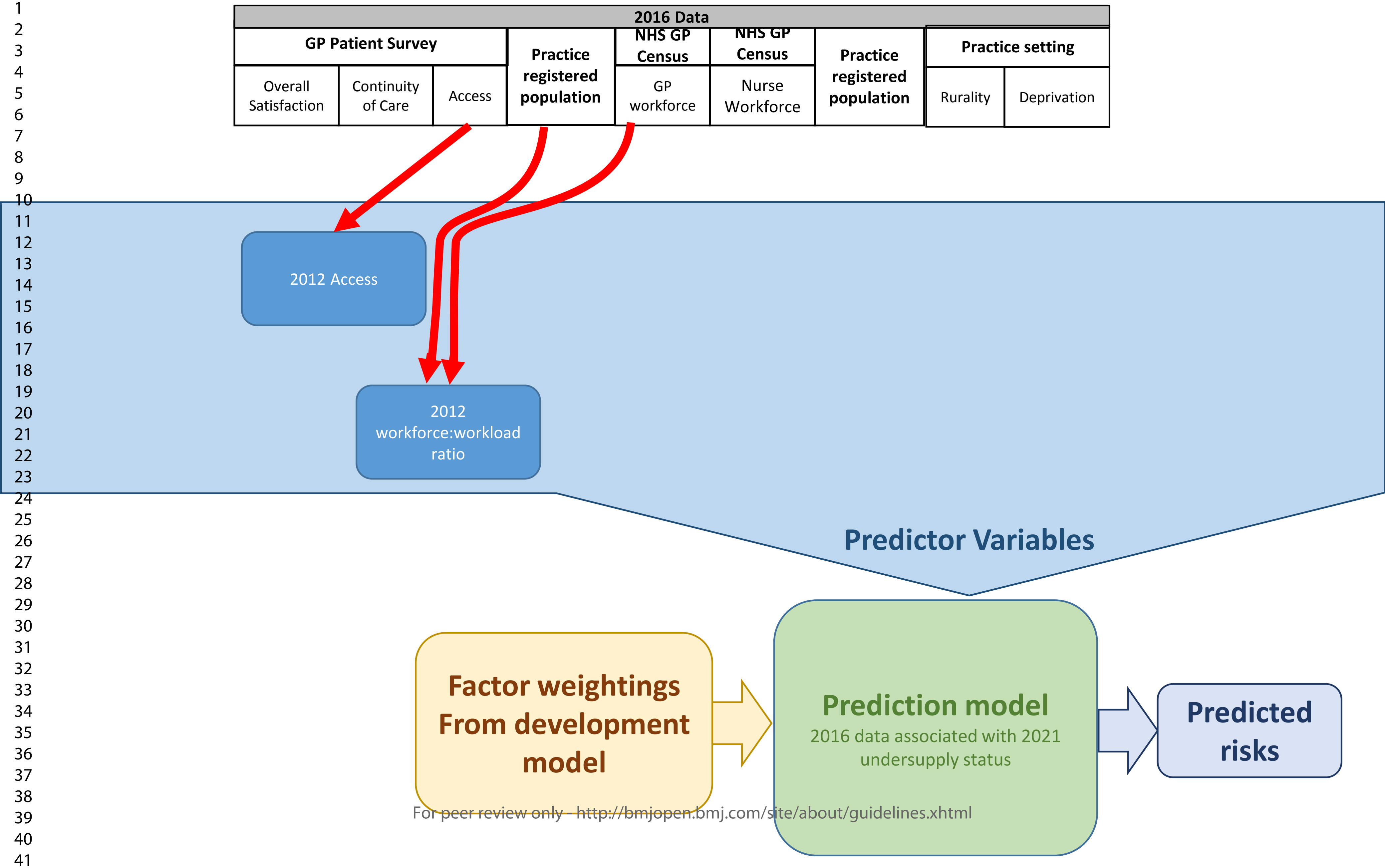

For peer review only - http://bmjopen.bmj.com/site/about/guidelines.xhtml

Appendix 3 – Calibration curve

In order to assess the calibration of the model we used predicted probabilities of being in undersupply from the development model (i.e. 2012 covariates and 2016 outcome) and split the practices into 10 groups according to deciles of this predicted probability. We then calculated the mean predicted probability in each group as well as the percentage of practices in undersupply in 2016. The relationship between these two quantities is shown in the figure below.

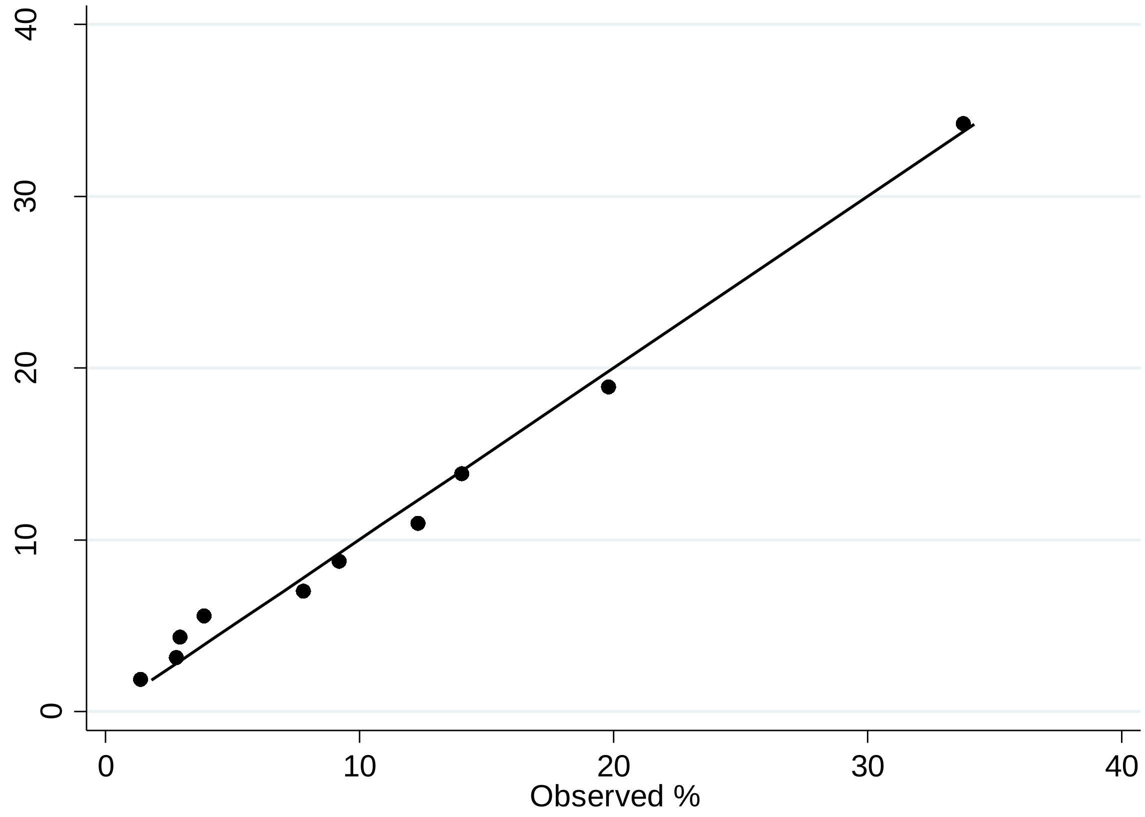

TRIPOD Checklist: Prediction Model Development

Section/Topic	Item	Checklist Item	Page
**Title and abstract**			
Title	1	Identify the study as developing and/or validating a multivariable prediction model, the target population, and the outcome to be predicted.	1
Abstract	2	Provide a summary of objectives, study design, setting, participants, sample size, predictors, outcome, statistical analysis, results, and conclusions.	2
**Introduction**			
Background and objectives	3a	Explain the medical context (including whether diagnostic or prognostic) and rationale for developing or validating the multivariable prediction model, including references to existing models.	4
	3b	Specify the objectives, including whether the study describes the development or validation of the model or both.	4
**Methods**			
Source of data	4a	Describe the study design or source of data (e.g., randomized trial, cohort, or registry data), separately for the development and validation data sets, if applicable.	4-6
	4b	Specify the key study dates, including start of accrual; end of accrual; and, if applicable, end of follow-up.	4-9
Participants	5a	Specify key elements of the study setting (e.g., primary care, secondary care, general population) including number and location of centres.	4-6
	5b	Describe eligibility criteria for participants.	7
	5c	Give details of treatments received, if relevant.	N/A
Outcome	6a	Clearly define the outcome that is predicted by the prediction model, including how and when assessed.	7
	6b	Report any actions to blind assessment of the outcome to be predicted.	N/A
Predictors	7a	Clearly define all predictors used in developing or validating the multivariable prediction model, including how and when they were measured.	4-9
	7b	Report any actions to blind assessment of predictors for the outcome and other predictors.	N/A
Sample size	8	Explain how the study size was arrived at.	7
Missing data	9	Describe how missing data were handled (e.g., complete-case analysis, single imputation, multiple imputation) with details of any imputation method.	7
Statistical analysis methods	10a	Describe how predictors were handled in the analyses.	7-9
	10b	Specify type of model, all model-building procedures (including any predictor selection), and method for internal validation.	9
	10d	Specify all measures used to assess model performance and, if relevant, to compare multiple models.	8-9
Risk groups	11	Provide details on how risk groups were created, if done.	8
**Results**			
Participants	13a	Describe the flow of participants through the study, including the number of participants with and without the outcome and, if applicable, a summary of the follow-up time. A diagram may be helpful.	9
	13b	Describe the characteristics of the participants (basic demographics, clinical features, available predictors), including the number of participants with missing data for predictors and outcome.	9
Model development	14a	Specify the number of participants and outcome events in each analysis.	9
	14b	If done, report the unadjusted association between each candidate predictor and outcome.	N/A
Model specification	15a	Present the full prediction model to allow predictions for individuals (i.e., all regression coefficients, and model intercept or baseline survival at a given time point).	18
	15b	Explain how to the use the prediction model.	N/A
Model performance	16	Report performance measures (with CIs) for the prediction model.	11
**Discussion**			
Limitations	18	Discuss any limitations of the study (such as nonrepresentative sample, few events per predictor, missing data).	13
Interpretation	19b	Give an overall interpretation of the results, considering objectives, limitations, and results from similar studies, and other relevant evidence.	12-13
Implications	20	Discuss the potential clinical use of the model and implications for future research.	14
**Other information**			
Supplementary information	21	Provide information about the availability of supplementary resources, such as study protocol, Web calculator, and data sets.	N/A
Funding	22	Give the source of funding and the role of the funders for the present study.	3

We recommend using the TRIPOD Checklist in conjunction with the TRIPOD Explanation and Elaboration document.

For peer review only - http://bmjopen.bmj.com/site/about/guidelines.xhtml

BMJ Open

Workforce predictive risk modelling – development of a model to identify general practices at risk of a supply□demand imbalance.

Journal:	*BMJ Open*
Manuscript ID	bmjopen-2018-027934.R2
Article Type:	Original research
Date Submitted by the Author:	24-Oct-2019
Complete List of Authors:	Abel, Gary; University of Exeter, University of Exeter Medical School (Primary Care)<br>Gomez-Cano, Mayam; University of Exeter Medical School<br>Mustafee , Navonil ; University of Exeter Business School<br>Smart, Andi; University of Exeter Business School<br>Fletcher, Emily; University of Exeter Medical School, Primary Care Research Group<br>Salisbury, Chris; University of Bristol, Academic Unit of Primary Health Care<br>Chilvers, Rupa; Tangerine Bee<br>Dean, Sarah; PenCLAHRC University of Exeter Medical School,<br>Richards, Suzanne; University of Leeds, Academic Unit of Primary Care; University of Leeds, Academic Unit of Primary Care<br>Warren, F; University of Exeter,<br>Campbell, John; University of Exeter, Primary Care;
**Primary Subject Heading**:	General practice / Family practice
Secondary Subject Heading:	Health policy, Health services research
Keywords:	PRIMARY CARE, Workforce, Supply-Demand, Organisation of health services < HEALTH SERVICES ADMINISTRATION & MANAGEMENT

SCHOLARONE™
Manuscripts

Workforce predictive risk modelling – development of a model to identify general practices at risk of a supply–demand imbalance.

Gary Abel *Senior Lecturer*[1]*, Mayam Gomez-Cano *Research Associate*[1], Navonil *Mustafee Associate Professor*[2], Andi Smart *Professor of Operations & Process Management*[1], Emily Fletcher *Research Fellow*[1], Chris Salisbury *Professor of Primary Health Care*[3], Rupa Chilvers *Director of Research and Knowledge Transfer4*, Sarah *Dean Professor in Psychology Applied to Rehabilitation and Health*[1], Suzanne Richards *Professor of Primary Care Research*[,5], Fiona Warren *Senior Lecturer*[1], John Campbell *Professor of General Practice and Primary Care*[1]

[1] University of Exeter Medical School, Exeter, UK

[2] University of Exeter Business School, Exeter, UK

[3]Centre for Academic Primary Care, NIHR School for Primary Care Research, School of Social and Community Medicine, University of Bristol, Bristol, UK

[4]Tangerine Bee, Exeter, UK

[5] Academic Unit of Primary Care, Leeds Institute of Health Sciences, UK

*Corresponding author:

Dr Gary Abel
Primary Care Research Group
University of Exeter Medical School
Smeall Building, St Luke's Campus
Magdalen Road
Exeter
EX1 2LU
g.a.abel@exeter.ac.uk

Word count (excludes title page, abstract, references, figures, tables): 4745

Key Words. Primary Care, workforce, supply-demand, organisation of care

For peer review only

Objective. To develop a risk prediction model identifying general practices at risk of workforce supply–demand imbalance

Design. Secondary analysis of routine data on general practice workforce, patient experience and registered populations (2012 to 2016), combined with a census of GPs' career intentions (2016).

Setting/Participants. A hybrid approach was used to develop a model to predict workforce supply–demand imbalance based on practice factors using historical data (2012–2016) on all general practices in England (with over 1000 registered patients n=6,398). The model was applied to current data (2016) to explore future risk for practices in South-West England (n=368).

Primary outcome measure. The primary outcome was a practice being in a state of workforce supply–demand imbalance operationally defined as being in the lowest third nationally of access scores according to the General Practice Patient Survey and the highest third nationally according to list size per full time equivalent GP (weighted to the demographic distribution of registered patients and adjusted for deprivation)

Results. Based on historic data, the predictive model had fair to good discriminatory ability to predict which practices faced supply–demand imbalance (area under ROC curve=0.755). Predictions using current data suggested that, on average, practices at highest risk of future supply–demand imbalance are currently characterised by having larger patient lists, employing more nurses, serving more deprived and younger populations, and having considerably worse patient experience ratings when compared with other practices. Incorporating findings from a survey of GP's career intentions made little difference to predictions of future supply–demand risk status when compared with expected future workforce projections based only on routinely-available data on GPs' gender and age.

Conclusions. It is possible to make reasonable predictions of an individual general practice's future risk of undersupply of GP workforce with respect to its patient population. However, the predictions are inherently limited by the data available.

For peer review only

Strengths and limitations of this study

- This study made use of freely available data from a range of sources to develop a predictive model of workforce supply–demand imbalance for general practices in England
- Historical data for all of England is used to develop factor weightings which are then applied to current data.
- The additional value of a census survey of career intentions of GPs in South West England is explored, comparing findings to predictions made on the basis of general practice workforce age and gender alone.
- The predictive model is inherently limited by the data available, and in particular we note that routine data of a measure of a practice's difficulty in recruiting staff were not available.

Data sharing statement. Most data used in this study are publicly available from referenced sources. Data from the GP Census survey can be made available on request from the corresponding author of the original publication at john.campbell@exeter.ac.uk.

Funding. The project was funded by the National Institute for Health Research, Health Service and Delivery Research programme (project 14/196/02). The views and opinions expressed herein are those of the authors and do not necessarily reflect those of the Health Service and Delivery Research programme, the National Institute for Health Research, the National Health Service, or the Department of Health.

Competing interests. All authors have completed the ICMJE uniform disclosure form at www.icmje.org/coi_disclosure.pdf (available on request from the corresponding author) and declare: grants from the National Institute for Health Research (Health Service and Delivery Research programme) during the conduct of the study. SD's position is partly supported by the National Institute for Health Research (NIHR) Collaboration for Leadership in Applied Health Research and Care South West Peninsula at the Royal Devon and Exeter NHS Foundation Trust. The views expressed are those of the author(s) and not necessarily those of the NHS, the NIHR or the Department of Health.

Ethics approval. Ethics approval for the GP Census survey was provided by the University of Exeter Medical School Research Ethics Committee. All other data was publicly available and so ethical approval was not required for its use.

For peer review only

Introduction

Against a backdrop of 34,495 full time equivalent (FTE) general practitioners (GPs) in 2016, the NHS in England saw a reduction of 3.5% of the English GP workforce (1193 FTE) in a single year.[1] This reduction has been seen in combination with rising demands of the patient population.[2] Such figures represent a 'crisis' in respect of GP workforce capacity, with particular problems in retaining established GPs in direct patient care [3 4]. Similar problems in respect of family doctor recruitment and retention are evident in other western healthcare economies and jurisdictions [5 6], and many countries have explored what might constitute optimal skill mix amongst primary care health professionals over the last 40 years [7-9].

There is, however, a need for the rational deployment of the GP workforce resource.[10 11] Various models exist to inform that deployment, with GP workload representing a key issue amongst individual GPs electing to quit patient care [3]. Gaining an understanding of GP workload pressures is also the basis of identifying any potential mismatch between the demand for general practice services, and the supply of GPs to meet that demand. In many countries, the general practice represents a key element in the delivery of primary care and acts as the basis for general practice workforce planning. For example, practices are the basis of reporting of patients' experience of primary care in England, captured using the General Practice Patient Survey (GPPS) [12].

The aim of this research was to develop a method to identify NHS general practices in one region of England which may face supply–demand workforce imbalances within the next 5 years. Previous workforce modelling in the UK has focussed upon deriving insights from analyses at the regional or national (macro) level [13]. In contrast, the research we are reporting here focuses on undertaking predictive risk modelling at a practice (micro) level. Routine workforce modelling makes use of data on doctors' age and gender, and historical retirement patterns. Here we consider whether surveying GPs' career intentions adds value to such modelling.

The first step in developing a predictive model to identify general practices at risk of future supply–demand imbalance is to define what is meant by a supply–demand imbalance and to operationalise this with measurable quantities. Assessing the supply of GP workforce at any one general practice is reasonably straightforward, however, assessing the demand of patients is complex as unmet demand is, by its nature, hard to quantify. Instead, here we consider the expected workload given the demographics of the patient population served. The balance between supply and demand within this framework is then represented by the expected workload per practitioner. However, high workload alone may not be an issue. Practices with high workload may meet patient demand through innovative and efficient systems of service delivery. High workload is considered to have a negative impact only when service delivery is impaired. For the purposes of this study we defined those practices with high workload per practitioner in combination with an inability to meet patient

For peer review only

demand as being in a state of 'under-supply'. Here we use the term 'under-supply' to indicate a practice which has a high demand from patients for a given supply of doctorswhich appears to be having a detrimental impact on services [14]. In this study we used a measure of patient access as a proxy for the ability to meet patient demand, in the belief that access is an important measure, reflecting the ease with which patients might engage with the primary healthcare system [14 15].

Methods

Overview

Several data sources have been brought together in this work. Analyses were performed at general practice level, firstly, to identify practices which were currently in 'under-supply' and, secondly, to identify those which are likely to have such problems in future. A predictive risk model (to predict the risk of a practice being in a state of 'under-supply' within 5 years) was developed by assessing the associations between current (2016) 'under-supply' status and historical routinely collected data (where available) on GP workforce, practice characteristics (rurality, deprivation, population) patient experience scores from 2012. The model further incorporated projected future populations in each area and considered projected future GP workforce based on GPs stated career intentions (from a survey of GPs). The rationale for this approach was to obtain factor weightings informed by evidence developed on past data. This model was then used to identify practices and areas in South West England that are likely to experience a supply–demand imbalance ('under-supply') in the future.

Data sources

Except where specified, national data for England were obtained and processed. A summary of data sources is given below with full details given in Appendix 1 along with a schematic illustrating the data flow used in the modelling process (Appendix 2).

General Practice Patient Survey (GPPS)

The GPPS is a national postal survey of patients' experience of primary care in England distributed to around 2.8 million adult patients each year [12]. We used data from the 2011/12 and 2015/16 surveys, during which the contents of the survey remained largely consistent. Response rates were 38% in 2011/12 (1,037,946 responses) and 39% in 2015/16 (836,312 responses) with an average of around 125 respondents per practice.

Workforce

Workforce data at practice level were obtained from NHS Digital and related to GP Census data taken as at 30 September 2012, 2013 and 2016 [16-18].

For peer review only

General practitioner quitting intentions

Self-reported GP intentions to cease practice were collected through a census survey which has been reported elsewhere [19]. Briefly, a questionnaire was administered to all active GPs in South West England in April-June 2016, enquiring about their intentions to cease/interrupt practice within 2 and 5 years (3370 questionnaires sent, 2248 returned, response rate 67%).

Practice rurality and deprivation

Practice rurality (rural/urban) based on an Office for National Statistics (ONS) categorisation of the postcode of the practice was obtained, as was a practice deprivation score based on the 2015 Index of Multiple Deprivation (IMD) [20].

Practice registered population

Data on the registered populations for each general practice were obtained for each quarter from April 2014 to April 2016 (9 datasets); as well as April 2012. These datasets provided the count of patients of each gender (male, female) by 5-year age-band strata.

Subnational population projections

We made use of the Office for National Statistics (ONS) subnational population projections developed to inform the local planning of healthcare and other public services for geographically defined populations served by Clinical Commissioning Groups (CCGs, organisations responsible for commissioning NHS services) [21]. These projections are demographic, trend-based projections that indicate the 'likely levels of future population' and are routinely produced every 2 years. We extracted projected populations for 2021 for the eight CCGs within South West England. Projections were made in 5-year age-bands for each gender.

Variables

Brief details are given below with full details in Appendix 1

Patient experience

We used three GPPS items GPPS reflecting access ("Last time you wanted to see or speak to a GP or nurse from your GP surgery: Were you able to get an appointment to see or speak to someone?"), continuity of care ("How often do you see or speak to the GP you prefer?"), and overall experience ("Overall, how would you describe your experience of your GP surgery?"). Case-mix adjusted practice scores for patient experience were created following previous methodology [22,23] adjusting for patient age, gender, ethnicity, presence of a long-term condition, and deprivation, using mixed effects logistic regression. The case-mix adjusted scores were based on dichotomous outcomes and used in the form of log-odds ratios relative to the average practice nationally.

Workforce

Practices with less than 0.5 GP FTE (38 out of 7,484 practices in 2012 data and 41 out of 6,709 practices in 2016 data) were excluded from all analyses on the basis that such a low

For peer review only

staff record indicated either that these were unusual practices or that the workforce data were in error. In addition to total GP FTE, the ratio of nurse FTE to doctor FTE and the ratio of doctor FTE in the "other" category to total doctor FTE were calculated (where "other" is assumed to mostly be locum GPs given that registrars, salaried GPs, and those on retainer schemes, are captured in specific categories). Total nurse FTE data were not available in 2012, so 2013 data were used in its place).

Workload

We used a definition of workload based on registered patients rather than on recorded patient visits. Patient visits are a measure of actual work undertaken which is limited by the workforce available, and so cannot capture unmet demand. By focussing on the registered population, we estimated the expected workload to serve that population based on national averages. Weights were applied to patient list sizes in order to standardise for the age and gender composition of the practice population, accounting for the fact that GPs spend longer, on average, consulting with patients who are very young, are older, or are female [2]. Further adjustment was made for the deprivation of the practice population to reflect higher health needs. These adjusted weighted list sizes were divided by the total GP FTE to obtain a measure of workload per GP FTE. Initial inspection of the workload figures showed that the distribution contained some infeasibly large and small values. Practices in the top and bottom 2.5% of the distribution were excluded from all further analysis. This exclusion took place following the removal of practices with less than 0.5 GP FTE.

Expected remaining future workforce

We estimated the proportion of GP FTE that would be expected, on average, to remain in patient care in 5 years' time. We did this in two principal ways: (i) using information on the age and gender of GPs at the practice along with previous work which identified the probability that GPs of different ages and genders leave patient care [24] and (ii) based on responses to survey of GP career intentions. The former was done for both 2012 and 2016 data and the latter only for 2016 data. The approaches are detailed in full in Appendix 1.

Outcome definition

Ability to meet patient demand was quantified using the GPPS access measure (ability to make an appointment), reflecting the ease with which patients might engage with the primary healthcare system. Workload to workforce ratio was quantified using the workload per GP FTE quantity described above. Practices that were in the lowest third of GPPS access scores and also in the highest third of workload per GP FTE nationally were defined as being in 'under-supply' (i.e. demand exceeded supply). Having used relative measures and cut points which were defined pragmatically for the purposes of this study in our definition of undersupply, we do not propose absolute and objective measures about whether a practice is 'failing' to deliver care. Indeed, if provision of care were good everywhere and the supply of workforce were not an issue, such an approach would be inappropriate. However, in the current climate in the UK, this represents a pragmatic approach in the absence of a direct measure.

For peer review only

Development of predictive risk model

Historical data were used to produce model coefficients which could then be applied to current data. Model development was based on all available national data in order to maximise statistical power .We did not split the data into development and validation samples as changes over time in healthcare delivery are more likely to be a threat to future use of the model than over-fitting. Predictor variables (as shown in Appendix 2a) were based on 2012 data unless otherwise noted and included

- three GPPS scores
- adjusted weighted list size per GP FTE (workforce to workload ratio)
- total GP FTE
- the ratio of "other" GP FTE to total GP FTE
- the expected proportion of GP FTE still in patient care in 2017
- ratio of nurse FTE to doctor FTE (using nurse FTE data from 2013)
- 2016 adjusted weighted list size (using 2016 data)
- rurality setting (based on 2016 data, but not expected to change)
- practice deprivation (based on 2016 data, but not expected to change)

We did not attempt to predict the 2016 practice populations using only data available in 2012 and instead included the observed 2016 practice populations as an additional explanatory variable due to a lack of data available for 3 years prior to 2012.

A logistic regression model was used with a binary outcome of a practice being in a state of under-supply in 2016 based on 2016 data (see outcome definition above). Practices were the unit of analysis. All variables considered were included and retained regardless of statistical significance.

We recognised the need to account for the fact that GPs leaving patient care would be most likely to impact the supply–demand balance when recruitment of staff was difficult. We were unable to obtain any direct measure of the difficulty any one practice had in recruitment and so instead we explored the use of three proxy measures:

1. The use of locums (operationalised as the proportion of total GP FTE falling in the "Other" category using NHS workforce data), on the basis that practices are likely to make greater use of locums when they are struggling to recruit partners or salaried GPs;

2. Patient access (using GPPS scores), on the basis that when there is a prolonged period where a practice is understaffed access may be compromised; and

3. The use of nurses (operationalised as the ratio of total nurse FTE to total GP FTE using NHS workforce data), on the basis that practices that have difficulty in recruiting GPs may employ more nurses to take on aspects of patient care traditionally delivered by GPs, thus freeing up GP time.

For peer review only

In exploratory analysis, an interaction between the expected proportion of the GP workforce remaining in patient care after 5 years and each of the identified proxy measures (use of locums, access, use of nurses) individually were included in the predictive model in turn. There was no evidence that either locum use or access modified the effect, in the model, of the expected proportion of the GP workforce remaining in patient care. However, there was weak evidence that the use of nurses did modify the effect of the expected proportion of the GP workforce remaining in patient care. This interaction was, therefore, retained in the final model. The predictive value of our model was assessed using a ROC (Receiver Operating Characteristic) curve analysis of predicted probabilities for all practices in England based on the data used to build the model (i.e. 2012 data and 2016 supply–demand imbalance classifications). So as to improve the generalisability of our findings and account for the fact that there will be a degree of over fitting in our model we employ 10-fold cross validation to estimate the area under the ROC curve[25] These were compared with a simpler model developed using only two explanatory variables which were 2012 data for factors defining the under-supply (GPPS access scores and adjusted weighted list size per FTE, noting that the outcome of the model, under-supply was still based on 2016 data, Appendix 2c). Calibration was assessed by comparing the mean predicted probability from the main model and the percentage of practices in undersupply in 2016 for deciles of predicted probability. We also performed a sensitivity analysis to examine the impact of excluding the top and bottom 2.5% of practices in terms of workload per GP FTE. To do so we re-ran the logistic regression after excluding only the top and bottom 1% of practices in terms of workload per GP FTE.

Future risk prediction

The coefficients from the historical model were applied to 2016 data to form our baseline risk predictions with a 5-year forward view for practices in South West England only (as shown in Appendix 2b). The reason for the restriction to those practices was that they were the only ones for which we had survey responses on future career intentions). It should be noted that although the original outcome definition was a relative one, the model treated them as absolute. In other words, predictions obtained from the model identify the risk of having a workload to workforce ratio in 2021 higher than two-thirds of practices did in 2016 and a GPPS access score in 2021 lower than two-thirds of practices did in 2016. In the context of a nationally worsening situation this would allow for considerably more practices to be in a state of undersupply. Practices in the highest 25% of the predicted risk profile were flagged as “high risk” of future under-supply of GP workforce, those in the lowest 25% were flagged as being “low risk”, and those in between were flagged as being at “moderate risk”.

The usefulness of the career intention survey was examined by comparing the above prediction with an alternative prediction using the expected proportion of the GP workforce remaining in patient care in five years’ time based only on the routinely available age and gender profile of GPs in the practice.

In addition to baseline predictions, we explored a number of `stress testing’ scenarios. These scenarios can be considered as stress tests of the model to identify practices that

might be more (or less) vulnerable to particular challenges. First, we explored the effect of increased difficulty in recruiting GPs, which we modelled as an increase in the coefficient for the expected proportion of GPs remaining in patient care (where an increased coefficient implies a greater impact of GP workforce leaving patient care). Second, we explored which practices might be at particular risk of a marked increase in local population. This was done by inflating the predicted adjusted weighted list size. The following scenarios were explored:

A. The coefficient for expected proportion of GPs remaining in patient care increased by 2 (equivalent to a 22% increase in the odds of being in supply–demand imbalance when 10% of GPs are expected to leave representing a modest increase in the difficulty of recruiting GPs);
B. The coefficient for expected proportion of GPs remaining in patient care increased by 4 (equivalent to a 49% increase in the odds of being in supply–demand imbalance when 10% of GPs are expected to leave representing a substantial increase in the difficulty of recruiting GPs);
C. The predicted adjusted weighted list size increased by 20%;
D. The predicted adjusted weighted list size increased by 40%;
E. A modest increase in difficulty recruiting GPs combined with a 20% increase in list size (a and c combined); and
F. A substantial increase in difficulty recruiting GPs combined with a 40% increase in list size (b and d combined).

For each of these scenarios, practices were rated according to relative risk (i.e. top 25% were labelled “high relative risk” as above) and absolute risk. The relative risk cut-offs in the baseline scenario were used for absolute risk cut-offs in the other scenarios.

Patient and public involvement

This study was part of a wider programme of work considering GP workforce issues which was served by a Patient and Public Involvement (PPI) group which provided input to the overall design and conduct of the research. Developing methods and results were shared at project management group meetings, which included PPI representatives who directly contributed to refining methods, and interpreting and contextualising the results.

Analyses were performed using Stata V14 and V16 and the 10-fold cross-validation was performed using the CVAUROC command.

For peer review only

Results

Mapping the current situation

A total of 6,398 practices in England had available data on all data items and had list sizes>1000; 371 of these were in South West England. The distribution of practices in England as a whole and South West England is shown in Figure 1. Practices with GPPS access scores (ability to make an appointment - our proxy for ability to meet patient demand) in the highest scoring third nationally were over-represented in South West England, with 57% of practices in this region falling in that category. There was also an under-representation of South West practices nationally in respect of workload (only 22% of practices in the region were classified as in the third of practices nationally with the highest workload). As a result, the percentage of practices defined as currently being in under-supply was considerably lower in South West England (5.1%) than in England as a whole (13.5%).

There was no evidence that list size varied between those practices in under-supply and other practices in South West England (Table 1). However, there was evidence that practices in under-supply had fewer FTE GPs. Together, these findings indicate that observed differences in workload are driven more by the supply of GP workforce than the demand of the registered patient population. Practices in undersupply also had a higher ratio of nurse FTE to GP FTE, served more deprived populations, had lower patient experience scores, had fewer patients over the age of 65, and were more likely to be in urban areas.

Predictive risk model

The regression coefficients for the logistic model are shown in Table 2 Predictive risk model coefficients estimated using 2012 data where possible to estimate the independent association with 2016 undersupply status. A negative coefficient implies a reduced risk of future undersupply as the value of the variable increases when all other variables are kept constant. We note the interaction between the expected proportion of GP FTE still working in patient care in 5-years' time and the ratio of nurse FTE to doctor FTE had a relatively large p-value (0.177). In initial modelling (before excluding practices on the basis of data quality) this interaction variable had a smaller p-value (0.06) indicating some evidence that it was worth including. When exclusions were applied, the coefficient did not change meaningfully. This fact, combined with the *a-priori* expectation that the effect of expected future GP workforce would be dependent on recruitment, provided support to retain the interaction term. The sensitivity analysis excluding only the top and bottom 1% of practices in terms of workload per GP FTE produced broadly similar regression coefficients with the exception of the coefficient for the expected proportion of GP workforce to remain in patient care which was reduced by 43% (results not shown).

Figure 1 Distribution of practices in England and in south-west England across categories according to workforce to workload ratio and GPPS access scores.

For peer review only

Figure 2 10-fold cross validation ROC curve for the predictive risk model based on the national historical data used to build the model.

shows the 10-fold cross validation ROC curve derived from the development model (i.e. 2012 covariates and 2016 outcome). The mean area under the curve was 0.755. The ROC curve from the simpler model only including the defining factors (GPPS access scores and adjusted weighted list size per FTE) had a meanarea under the curve of 0.695, suggesting that the additional variables included in our model provided a modest, but meaningful, improvement in predictive value. A visual inspection of a calibration plot for the full model suggests that there is good calibration of the model (Appendix 3).

Future risk predictions

Applying the risk prediction model to data from 2016, seeking to predict the risk of future supply–demand imbalance for individual practices in South West England, we obtained risk scores for 368 practices with available data remaining after applying exclusions. The median probability of future supply–demand imbalance across practices was 5.4% (IQR 2.8% to 10.0%). In total 40 (10.9%) practices had a risk greater than 20%, and 12 (3.3%) had a risk greater than 50%. Table 3 shows the characteristics of those practices in South West England classified as high risk (top 25% of practices, corresponding to an absolute risk of 10% or greater) of being in a state of under-supply compared with other practices. In contrast to the current situation shown in Table 2, there was no evidence (p=0.445) that the total GP FTE varies between high/other risk classification. There was evidence, however, that all other descriptive factors varied between the two groups. Practices at “high risk” of future supply–demand imbalance tended to currently have larger list sizes, to have a higher proportion of nurses in the workforce, to serve more deprived and younger populations, have considerably worse GPPS scores, and were more likely to be in urban areas.

Stress testing scenarios

Figures 2 and 3 illustrate the changes to the relative and absolute risk of future under-supply under different stress testing scenarios. In this figure, each practice is represented by a horizontal bar. The vertical ordering of each practice is the same in each scenario, and is based on the rank ordering of each practice according to the baseline risk prediction. For each scenario, the colouring of every practice’s horizontal bar illustrates the relative or absolute risk classification (Figure 3 and 4 respectively) such that changes in colour indicate changes in risk classification. In Figure 3 practices coloured red (high risk) are in the top 25% of practices in terms of risk of undersupply for any given scenario, practices coloured green (low risk) are in the bottom 25% for any given scenario, with the middle 50% of practices coloured yellow. In Figure 4 practices coloured red (high risk) have an absolute risk of future undersupply greater than 10% (corresponding to the minimum absolute risk of future undersupply of the top 25% of practices in the baseline scenario), practices coloured green

For peer review only

(low risk) have an absolute risk less than 2.8% (corresponding to the maximum absolute risk of the bottom 25% of practices in the baseline scenario) and intermediate practices are coloured yellow.

Firstly, we examined the changes in predictions when using the two different methods of quantifying the likely future GP workforce remaining in patient care (one method using the results of the career intention survey and one method using only on GP age and gender). The two methods produced similar values for the likely proportion of GP workforce remaining in patient care with a Spearman correlation of 0.77 between the estimates made using the two methods in the 387 practices with at least one survey response. When using the different methods in the risk prediction model, there was very little difference in practices categorised as being either at “high relative risk” or “high absolute risk” of undersupply (seen in Figure as limited reclassification of practices, correlation of ranks=0.999).

In general, practices classified as being at “high relative risk” remained so under scenario A (modest increase in the difficulty of GP recruitment to replace those leaving - correlation in ranks between scenario a and baseline=0.97). However, there was a dramatic increase in the number of practices with a predicted absolute risk of future undersupply greater than 10% (seen as an increase in the number of practices coloured red Figure , scenario A). There was an even greater disturbance in the classification of practices under scenario B (illustrating the recruitment of GPs was becoming much harder), though the reclassification in terms of relative risk was still relatively modest (Figure , scenario B, correlation in ranks between scenario B and baseline=0.90). Conversely, the reclassification in terms of absolute risk (Figure 4, scenario B) was significantly greater; the majority of practices had a predicted risk above 10%.

Increasing the projected practice population resulted in only modest changes in respect of which practices are classified as being at “high relative risk”. Only a small relative increase was seen when comparing scenarios C and D with the baseline predictions (Figure correlation in ranks between scenario C and baseline=0.99 and scenario D and baseline=0.98). However, substantial changes were seen in the number of practices with an absolute risk of undersupply greater than 10% (Figure 4, scenarios C and D). Combining the effect of scenarios A and C resulted in relative risk classifications closer to the baseline predictions than scenario A alone. However, in terms of absolute risk, more practices had a risk greater than 10% (Figure 4, scenario A and scenario C).

When scenario B and scenario D were combined (illustrating a situation where it was much harder to recruit GPs combined with an increased practice population of 40%) it was evident that nearly all practices (88%) exceeded 10% absolute risk of supply–demand imbalance within 5 years, with only 9 (2.4%) practices classified as being at “low absolute risk” using the cut-offs derived from the baseline predictions.

For peer review only

Discussion

Summary of main findings

We developed an approach to modelling an individual general practice's future risk of being in a state of GP workforce undersupply. Within that work, we developed a 'main' model and a 'simpler' model. The 'main' model produced a range of risk scores attributable to practices across South West England, and, based on the ROC curve analysis, had a fair to good discriminatory ability. Applying our modelling approach suggests that the practices at highest risk of future undersupply of GP workforce are those which currently have, on average: larger patient lists, employ more nurses relative to doctors, serve more deprived and younger populations, or have considerably worse patient experience ratings when compared with national averages.

In an extension of our research, we also modelled scenarios where the recruitment of GPs was more difficult than at present and/or where practice populations increase dramatically beyond what would be expected from historical local trends (for example, through a new housing development). These scenarios did identify practices where risk profiles changed, sometimes substantially, but in general, it was the same practices in all scenarios that were at highest risk of future undersupply of GP workforce. This almost certainly reflects the fact that those most likely to have problems in the future are those which are currently experiencing difficulties. This was evident from the relatively good predictions from a simple model including only contributing variables (i.e. workload per FTE GP and GPPS patient access scores);this model had an area under the ROC curve that was not substantially less than that of the 'main' model, which drew on a wider range of variables, some of which were not routinely available in published data. In particular, we found that inclusion of findings from our own survey of GPs' career intentions had very little impact on the predictions when compared with using expected future workforce projections based only on routinely available data regarding GPs' gender and age.

Strengths and weaknesses

Strengths of this work include the comprehensive use of freely available data as well as the exploratory use of a census survey of career intentions of GPs in the region. The main strength is the novel development of factor weightings based on routinely available historical data. However, we recognise that this assumes that factors driving changes are constant from the historical time period of model development to the future time period of prediction. This is unlikely to be the case given recent problems in GP workforce recruitment and retention in the UK [4]. To this end we have modelled what might be expected if recruitment was harder than it has been historically, and if there were substantive increases in the practice population. These scenarios may be more reflective of what we might expect going forward.

For peer review only

The main weakness of this work concerns our ability to distinguish in what situations, and in which practices, future GP workforce leaving patient care will impact the level of continuing GP workforce and its ability to meet patient demand. For practices that do not encounter problems in recruiting GPs, retiring GPs pose much less of an issue than for practices where recruitment is difficult. Here we relied on the level of nurse staffing in a practice as a proxy for recruitment issues; importantly, this means the association of more nurses with at-risk practice status is likely to be attributable to practices being unable to fill GP vacancies, not that more nurses per se puts a practice at risk. A more direct measure of recruitment problems which was consistently and widely collected (such as duration of advertising for vacant posts, using a consistent methodology to track this) would be expected to provide a better model. Unfortunately, no robust freely available measure exists. The NHS GP census does collect data on time to fill vacancies [18] and existing unfilled vacancies. However, these data are not freely available, and, furthermore, are not mandatory for completion by practices.

Another weakness was that historical workforce data were not available in the same detail as current data (including nurse data not being available for 2012 at all). This meant that future workforce predictions using historical data would not be as accurate as those using current data. These inaccuracies would lead to a loss of power, and potentially an attenuation of the associated regression coefficients. This may explain the low statistical significance of associated coefficients in the model.

Finally, we note that our assessment of the performance of our model was made on the same data the model was developed on, and thus may not be a reflection of the accuracy of future risk predictions. Validation of the future risk predictions would be welcome, but can only be undertaken in 5 years' time.

Implications

We have demonstrated that it is possible to make reasonable predictions of an individual general practice's future risk of undersupply of GP workforce with respect to its patient population. With ongoing GP workforce issues in the UK, local models are being developed to identify potentially "at-risk" practices [26]. However, unlike the model we present here, it is not clear to what extent these models are evidence-based or to what extent their limitations are recognised by the users of the models or even what is meant by "at risk".

Whilst the model we present here is set in the context of UK primary care, the general approach could be applied to other settings and in other locations. In all cases the predictions will be inherently limited by the quality and quantity of available data. Improvements in data quality going forward will help the situation in the UK, particularly if data are released on GP recruitment. However, it will be some time before robust historical data exist that can be used for the model development process outlined here. If models such as the one outlined here are to be produced and used, it is important that high-quality data continue to be collected. However, it is worth recognising that the full range of data employed in the 'main' model produced only modest improvement in model fit over our 'simpler' model, suggesting that reasonable predictions may be made using a smaller

For peer review only

number of variables. We have not attempted to establish a minimum useful set of data to make predictions of risk of undersupply of GP workforce. Rather, we have focused on an approach by which such predictions can be made. Given that, the lack of availability of variables such as those used here should not present a barrier to developing a model along similar lines suitable for other settings.

The predictions produced by this model and similar models may facilitate targeting of interventions to retain and attract GP workforce either in specific practices, or in specific regions currently at high risk of problems driven by workforce supply. Although our model provides reasonable discrimination, much could potentially be achieved by focussing efforts on those practices currently experiencing difficulties.

Whilst a policy of targeted interventions may have value, we find that most practices are likely to be at a high risk of workforce undersupply when faced with a substantial increase in demand from an increased patient population combined with major difficulties in recruiting GPs. As such, local knowledge of drivers of increased practice populations, such as housing developments, will be key to being able to suitably apply targeted interventions. Even in South West England where workload and the ability to meet patient demand are better than in England overall, most practices are currently vulnerable to recruitment challenges, and will remain so going forward. Given this, national or broad regional policies and strategies may be more effective than targeted ones, especially if there is limited knowledge on how local populations are likely to evolve.

Contributions. GA, JC, AS and NM conceived the study. GA, MGC and NM performed analysis. GA, MGC, NM, AS, EF, CS, RC, SD, SR, FW and JC contributed to the design and interpretation of the study. GA drafted the initial manuscript. GA, MGC, NM, AS, EF, CS, RC, SD, SR, FW and JC commented and critically reviewed the manuscript prior to submission..

For peer review only

1. BMJ. Seven days in medicine: 1-7 February 2017. *BMJ* 2017;356 doi: 10.1136/bmj.j668
2. Hobbs FR, Bankhead C, Mukhtar T, et al. Clinical workload in UK primary care: a retrospective analysis of 100 million consultations in England, 2007–14. *The Lancet* 2016;387(10035):2323-30.
3. Sansom A, Terry R, Fletcher E, et al. Why do GPs leave direct patient care and what might help to retain them? A qualitative study of GPs in South West England. *BMJ Open* 2018;8(1) doi: 10.1136/bmjopen-2017-019849
4. Roland M, Everington S. Tackling the crisis in general practice. *BMJ* 2016;352 doi: 10.1136/bmj.i942
5. Kash BA, Ogden P, Popp E, et al. Exploring future models of primary care for Texas. *International Journal of Innovation Science* 2017;9(4):435-54. doi: doi:10.1108/IJIS-01-2017-0005
6. Miani C, Pitchforth E, Ellen N. Choice of primary care provider: a review of experiences in three countries: Policy Research Unit in Policy Innovation Research (PIRU), 2013.
7. Spitzer WO, Sackett DL, Sibley JC, et al. The Burlington randomized trial of the nurse practitioner. *The New England journal of medicine* 1974;290(5):251-6. doi: 10.1056/nejm197401312900506 [published Online First: 1974/01/31]
8. Freund T, Everett C, Griffiths P, et al. Skill mix, roles and remuneration in the primary care workforce: who are the healthcare professionals in the primary care teams across the world? *International journal of nursing studies* 2015;52(3):727-43. doi: 10.1016/j.ijnurstu.2014.11.014 [published Online First: 2015/01/13]
9. Laurant M, Reeves D, Hermens R, et al. Substitution of doctors by nurses in primary care. *The Cochrane database of systematic reviews* 2005(2):Cd001271. doi: 10.1002/14651858.CD001271.pub2 [published Online First: 2005/04/23]
10. Paddison C, Abel G, Campbell J. GPs: working harder than ever. *The British journal of general practice : the journal of the Royal College of General Practitioners* 2018;68(670):218-19. doi: 10.3399/bjgp18X695873 [published Online First: 2018/04/28]
11. Royal College of General Practitioners. Fit for the Future: a Vision for general practice, 2019.
12. NHS England. GP Patient Survey: NHS England; [Available from: https://gp-patient.co.uk/surveys-and-reports accessed 05/03/2018.
13. The Centre for Workforce Intelligence. In-depth review of the general practitioner workforce, 2014.
14. Campbell JL, Salisbury C. Research into practice: accessing primary care. *Br J Gen Pract* 2015;65(641):e864-e68. doi: 10.3399/bjgp15X688057
15. Campbell J, Smith P, Nissen S, et al. The GP Patient Survey for use in primary care in the National Health Service in the UK – development and psychometric characteristics. *BMC Fam Pract* 2009;10(57):Doi: 10.1186/471-2296-10-57.
16. NHS Digital. General and personal medical services, England 2002-2012 as at 30 September 2012 [Available from: http://content.digital.nhs.uk/searchcatalogue?productid=10382&topics=1%2fWorkforce%2fStaff+numbers&kwd=GP+staff&geo=England&sort=Relevance&size=10&page=2#top accessed 31/07/2017 2017.
17. NHS Digital. General and personal medical services, England 2003-2013 as at 30 September 2013 [
18. NHS Digital. General and personal medical services, England September 2015-March 2016, Provisional Experimental statistics 2016 [Available from: http://content.digital.nhs.uk/catalogue/PUB21772 accessed 31/07/2017 2017.
19. Fletcher E, Abel GA, Anderson R, et al. Quitting patient care and career break intentions among general practitioners in South West England: findings of a census survey of general practitioners. *BMJ open* 2017;7(4) doi: 10.1136/bmjopen-2017-015853

For peer review only

20. Public Health England. National General Practice Profile 2013 [Available from: http://fingertips.phe.org.uk/profile/general-practice accessed August 2104 2014.
21. Office for National Statistics. Subnational population projections for England: 2014-based projections 2014 [Available from: https://www.ons.gov.uk/peoplepopulationandcommunity/populationandmigration/populationprojections/bulletins/subnationalpopulationprojectionsforengland/2014basedprojections accessed 31/07/2014 2017.
22. Paddison C, Elliott M, Parker R, et al. Should measures of patient experience in primary care be adjusted for case mix? Evidence from the English General Practice Patient Survey. *BMJ Qual Saf* 2012
23. Abel GA, Saunders CL, Lyratzopoulos G. Cancer patient experience, hospital performance and case mix: evidence from England. *Future oncology (London, England)* 2014;10(9):1589-98. doi: 10.2217/fon.13.266 [published Online First: 2013/12/18]
24. Centre for Workforce Intelligence. In-depth review of the general practitioner workforce - Final report 2014.
25. Hastie T, Tibshirani R, Friedman J. The elements of Statistical Learning, Data Mining, Inference and Prediction.: Springer 2013.
26. Healthy London Partnership. Primary Care (GP) Workforce Model 2016 [Available from: http://healthylondon.org/hlp-archive/sites/default/files/The%20Primary%20Care%20Model%20Step-by-Step%20Guide.pdf accessed 05/03/18.

For peer review only

Table 1 Comparison of practices in South West England defined as in undersupply with other practices in the region.

	Under-supply (n=19)			Other (n=352)			
	**median**	**25%**	**75%**	**median**	**25%**	**75%**	**p value***
List size	9264	5361	11576	7598	5270	11077	0.448
Adjusted weighted list size	8959	5212	12287	8099	5638	11570	0.550
GP FTE	3.1	2	5.1	4.7	3.2	6.6	0.012
Ratio nurse/GP FTE	0.8	0.7	1	0.5	0.4	0.7	<0.001
Index of Multiple Deprivation	25.7	20.2	30.9	18.7	13.5	24.4	0.003
GPPS access‡	0.2	0.1	0.2	0.7	0.5	0.9	<0.001
GPPS continuity‡	0.2	0.2	0.3	0.6	0.4	0.8	<0.001
GPPS satisfaction‡	0.2	0.1	0.4	0.7	0.5	0.9	<0.001
% over 65	16.8	13.3	21	22.6	17.6	26	0.004
**Setting**	**n**	**%**		**n**	**%**		**p value†**
Urban practices	17	6.8		232	93.2		0.042
Rural practices	2	1.6		120	98.4		

*** from Mann–Whitney test**
† from Fisher's exact test
‡GPPS scores used were case-mix adjusted log-odds ratios relative to the average practice nationally
** IMD scores are given (rather than ranks) with higher scores indicating higher levels of deprivation.

For peer review only

Table 2 Predictive risk model coefficients estimated using 2012 data where possible to estimate the independent association with 2016 undersupply status.

Data type	Variable	Note on units	Logistic regression coefficient (95% CI)	p-value
GP Patient Survey Scores*	Access	Random effect (log-odds ratio) from logistic case-mix adjustment model	-0.96 (-1.21, -0.70)	<0.001
	Continuity of care		-0.09 (-0.25, 0.07)	0.274
	Overall Satisfaction		-0.48 (-0.70, -0.27)	<0.001
Baseline Workforce†	Ratio of nurse FTE to GP FTE		1.02 (-0.05, 2.09)	0.062
	Adjusted Weighted List Size per GP FTE	Per 1000 patients per GP FTE	0.40 (0.18, 0.62)	<0.001
	Total GP FTE		-0.17 (-0.25, -0.10)	<0.001
	Ratio of “Other” GP FTE to total GP FTE		0.65 (0.32, 0.98)	<0.001
Rurality Setting‡	Urban practice		Reference	0.404
	Rural practice		-0.13 (-0.43, 0.17)	
Index of Multiple Deprivation – practice in quintile‡	1 – least deprived		Reference	<0.001
	2		0.02 (-0.29, 0.32)	
	3		0.13 (-0.16, 0.42)	
	4		0.57 (0.29, 0.85)	
	5 – most deprived		0.36 (0.06, 0.66)	
Projected quantities	Adjusted Weighted List Size**	Per 1000 patients	0.14 (0.10, 0.18)	<0.001
	Proportion of GP FTE still in patient care*	Varies from 0 to 1	0.38 (-0.78, 1.54)	0.520
	Proportion of GP FTE still in patient care x Ratio of nurse FTE to GP FTE*		-1.01 (-2.48, 0.46)	0.177
Constant			-4.15 (-5.10, -3.21)	<0.001

*Data from 2012
†Data from 2012 except nurse data which were from 2013
‡IMD data from 2016 for variable where this status is expected to remain relatively constant over time
**Actual list size from 2016 rather than projected list size based on 2012 data as pre-2012 data did not allow projections comparable to those which were made with more current data looking forwards.

For peer review only

Table 3 Differences between practices identified at high risk of future undersupply and other practices assuming a baseline scenario.

	High risk (n=92)			Other (n=276)			
	**median**	**25%**	**75%**	**median**	**25%**	**75%**	**p value***
List size	10625	7732	13195	6915	4941	10206	<0.001
Adjusted weighted list size	11133	7369	13252	7398	5251	10615	<0.001
GP FTE	5	3.1	6.6	4.5	3.1	6.6	0.445
Ratio of nurse FTE to GP FTE	0.7	0.5	1	0.4	0.4	0.6	<0.001
IMD	25.6	18.7	31.7	17.6	13.1	22.2	<0.001
GPPS access‡	0.4	0.2	0.6	0.8	0.6	0.9	<0.001
GPPS continuity‡	0.3	0.2	0.5	0.7	0.5	0.9	<0.001
GPPS satisfaction‡	0.4	0.2	0.6	0.7	0.5	0.9	<0.001
% over 65	18.3	14.1	23.4	23.2	18.5	26.5	<0.001
Setting	**n**	**%**		**n**	**%**		**p value†**
Urban practices	77	31.3		169	68.7		<0.001
Rural practices	15	12.3		107	87.7		

*** from Mann–Whitney test**
† from Fisher's exact test
‡GPPS scores used were case-mix adjusted log-odds ratios relative to the average practice nationally

For peer review only

Figure 1 Distribution of practices in England and in south-west England across categories according to workforce to workload ratio and GPPS access scores.

For peer review only

Figure 2 10-fold cross validation ROC curve for the predictive risk model based on the national historical data used to build the model.

For peer review only

*Risk prediction as for baseline, but using age and gender of GPs alone rather than including responses to the career intentions survey. In each case the practices are ordered by the baseline scenario.

Figure 3 Rating of practices in South West England from different risk prediction scenarios a-d using cut-offs defined by the quartiles of each prediction (relative risk).

For peer review only

*Risk prediction as for baseline, but using age and gender of GPs alone rather than including responses to the career intentions survey. In each case the practices are ordered by the baseline scenario.

Figure 4 Rating of practices in South West England from different risk prediction scenarios a-d using cut-offs defined by the quartiles of the baseline prediction (absolute risk).

For peer review only

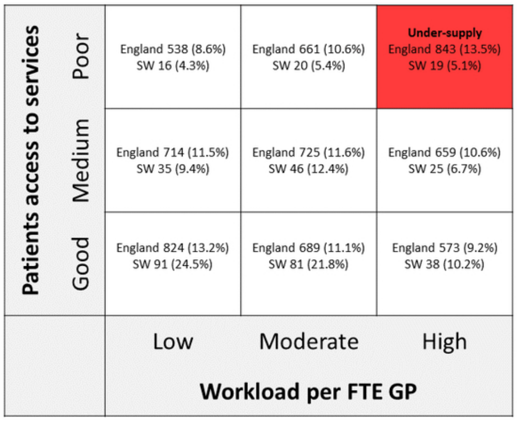

Figure 1 Distribution of practices in England and in south-west England across categories according to workforce to workload ratio and GPPS access scores.

90x50mm (300 x 300 DPI)

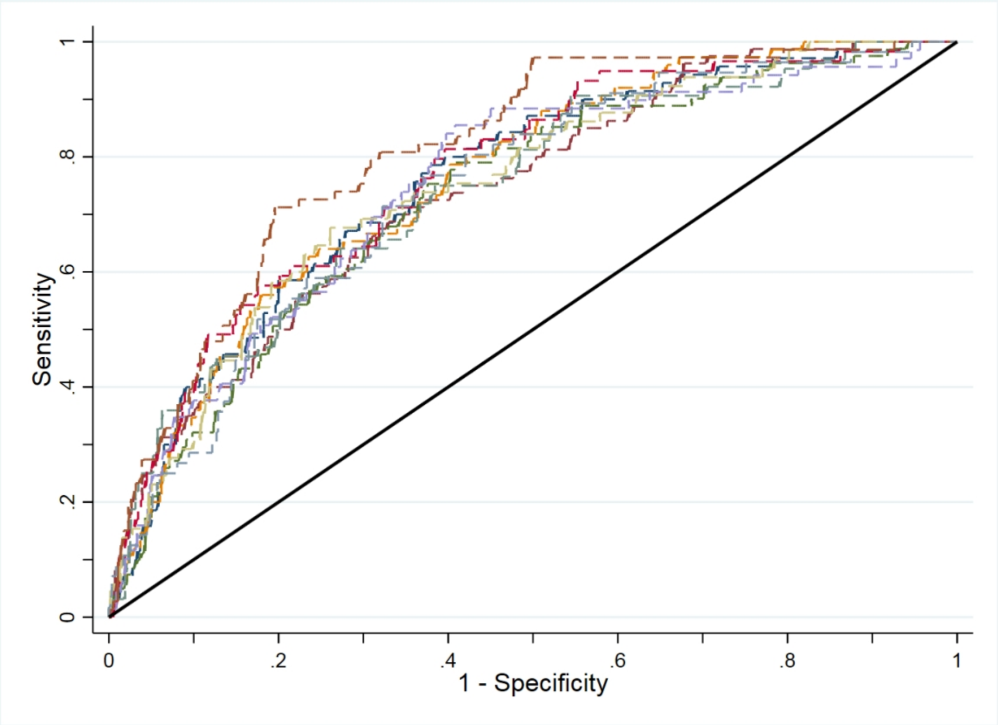

Figure 2 10-fold cross validation ROC curve for the predictive risk model based on the national historical data used to build the model.

116x84mm (300 x 300 DPI)

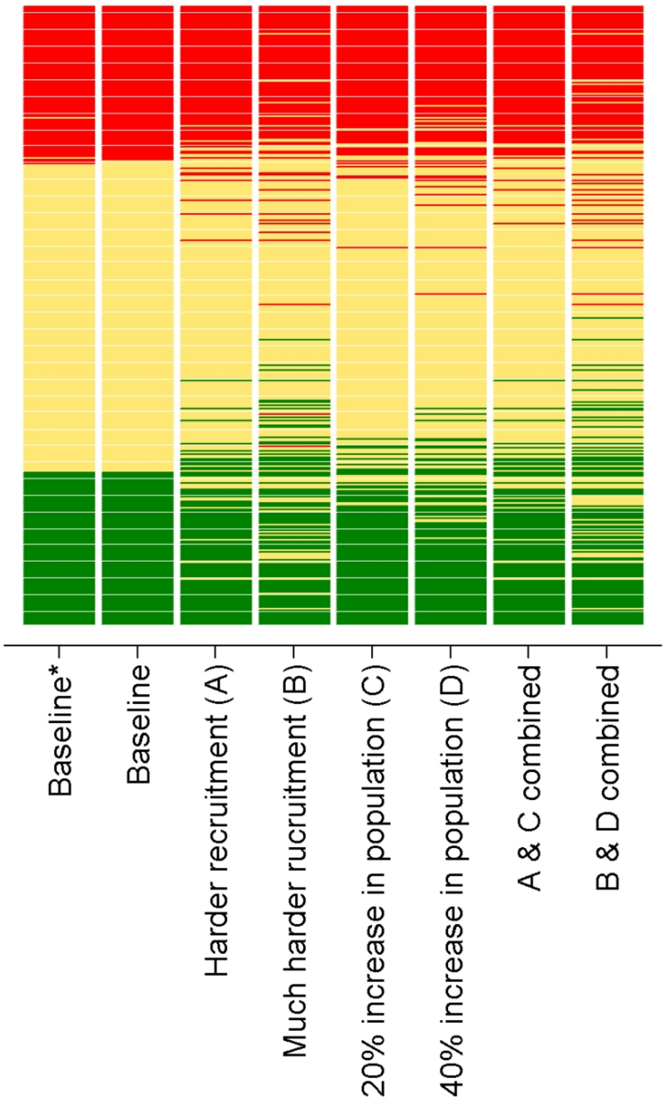

Figure 3 Rating of practices in South West England from different risk prediction scenarios a-d using cut-offs defined by the quartiles of each prediction (relative risk).

95x127mm (300 x 300 DPI)

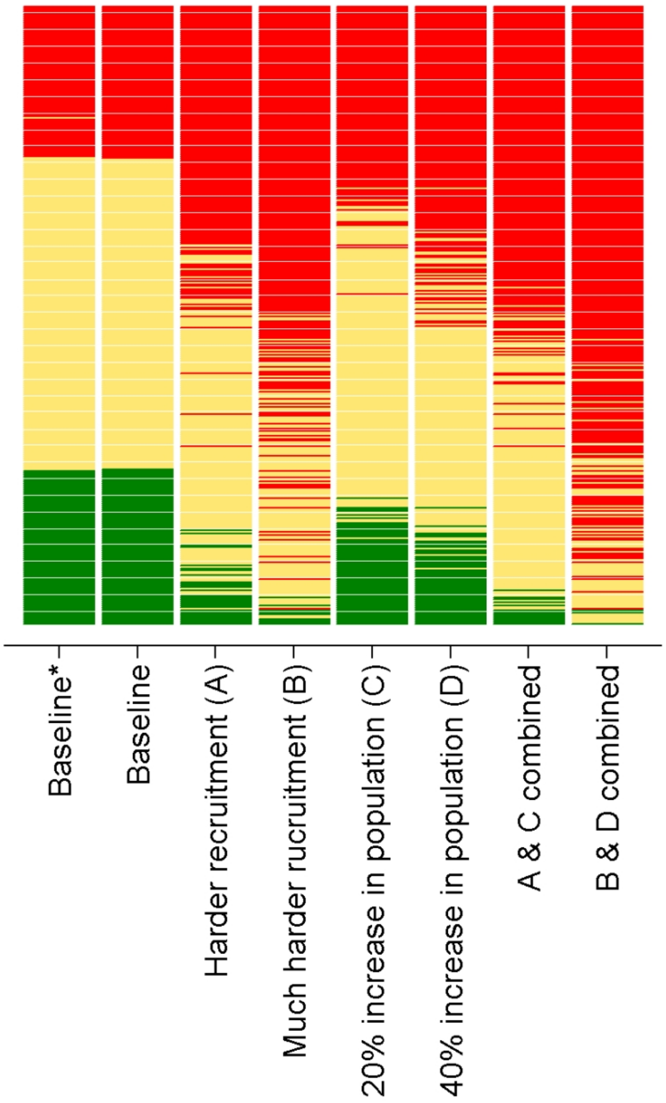

Figure 4 Rating of practices in South West England from different risk prediction scenarios a-d using cut-offs defined by the quartiles of the baseline prediction (absolute risk).

95x127mm (300 x 300 DPI)

Appendix 1 - Data sources and preparation

Except where specified, national data for England were obtained and processed. A summary of data sources and data flow used in the modelling process is presented in Appendix 2.

GP Patient Survey

The General Practice Patient Survey (GPPS) is a national postal survey of patients' experience of primary care in England. Patients from practices that are known from prior surveys to have low response rates are oversampled. Full details of the sampling strategy are published elsewhere.[1] We used data from the 2011/12 and 2015/16 surveys. The contents of the survey have remained largely consistent over this time period. Response rates were 38% in 2011/12 (1,037,946 responses) and 39% in 2015/16 (836,312 responses).

We made use of three items from the GPPS reflecting access, continuity of care and overall experience. For patient's experience of access the following question was used:

- *"Last time you wanted to see or speak to a GP or nurse from your GP surgery:" "Were you able to get an appointment to see or speak to someone?"*

Responses of "Yes" and "Yes, but I had to call back closer to or on the day I wanted the appointment" were coded as a positive response and responses of "No" were coded as a negative response. Responses of "Can't remember" were treated as uninformative and excluded from the analysis.

The item on ability to see a preferred doctor is taken as a proxy measure for continuity of care:

- *"How often do you see or speak to the GP you prefer?"*

Responses of "Always or almost always" and "A lot of the time" were coded as a positive response and responses of "Some of the time" and "Never or almost never" were coded as a negative response. Responses of "Not tried at this GP surgery" were treated as uninformative and excluded from the analysis.

Finally, an item capturing data on the patient's overall experience of care is included:

- *"Overall, how would you describe your experience of your GP surgery?"*

Responses of "Very good" and "Fairly good" were coded as a positive response and responses of "Neither good nor poor", "Fairly poor" and "Very poor" were coded as a negative response. There were no uninformative options for this question.

Due to certain patient groups tending to give more positive responses in patient surveys, case-mix adjusted practice scores were created. This was achieved using mixed effects logistic regression adjusting for patient age, gender, and ethnicity, presence of a long-term condition, and deprivation

For peer review only

(using the Index of Multiple Deprivation [IMD], an area based measure assigned according to the patient's residential postcode[2 3]) and a random intercept for practice. The case-mix adjustment provides scores for individual practices based on a standardised mix of patients. The case-mix adjusted scores were used in the form of log-odds ratios relative to the average practice nationally.

Workforce

Workforce data were obtained from NHS Digital and related to the GP Census data taken as at 30 September 2012, 2013 and 2016.[4-6] Each dataset gave the headcount of GPs in 5-year age-bands for each practice. The 2012 dataset contained total GP headcount by gender as did the 2016 dataset. In the 2016 dataset additional detail of GP FTE by gender was provided. Both datasets contained total GP FTE as well as GP FTE broken down by GP role. We also extracted the total nurse FTE from the 2016 dataset. As nurse FTE data were not available in 2012, the relevant data were extracted from the 2013 dataset in its place. From these data two further variables were derived: the ratio of nurse FTE to GP FTE; the ratio "other" category FTE to total GP FTE (where "other" is assumed to mostly be Locum GPs given that GP registrars, salaried GPs, and those on retainer schemes are captured in other categories). These data were also used in the derivation of workload and the predicted remaining future workforce.

Practices with less than 0.5 FTE GP (38 out of 7,484 practices in 2012 data and 41 out of 6,709 practices in 2016 data) were excluded from all analyses on the basis that such a low staff record indicated either that these were unusual practices or that the workforce data were in error. In the former case such unusual practices are not the focus of this work and in the latter case, erroneous inferences may have been made if they had been included.

GP quitting intentions

To predict remaining future workforce we utilised self-reported GP intentions to cease practice collected through a survey which formed part of the ReGROUP project and has been reported.[7] Briefly, a questionnaire was administered to all active GPs in South West England in April-June 2016, enquiring about their intentions to cease/interrupt practice in the next 2 and 5 years (3370 questionnaires sent, 2248 returned, response rate 67%). We combined responses to two questions:

- *"How likely is it that you will permanently leave direct patient care within the next 5 years?"*
- *"How likely is it that you will take a career break (or another career break) within the next 5 years?"*

Each question had response options of "Very Likely", "Likely", "Unlikely" and "Very unlikely". Where GPs gave different response options for these two questions, the response with the highest likelihood

of cessation or interruption was taken. This reflects the most likely chance of impact to future GP workforce in the next 5 years. We also used respondents' answers to the question:

- *"In your current/most recent direct patient role, how many sessions do/did you work in a typical week?"*

Free text responses to this question provided data from which an estimate of each responder's current FTE work commitment could be calculated. Working eight sessions per week was taken as 1 FTE, consistent with the approach used in the GP census.[6] When more than eight sessions was given as a response the FTE was capped at 1. If more than 24 sessions was given as a response it was assumed the question had been answered incorrectly and the data were treated as missing. Data for all GPs surveyed on age, gender and affiliated practice were obtained from the Performers List.

Practice rurality and deprivation

Practice rurality was contained within the GPPS 2016 dataset and was based on an Office for National Statistics (ONS) categorisation of the postcode of the practice. We used a rural/urban version of this categorisation. Practice deprivation score was obtained from Public Health England and was based on the 2015 IMD. Individual patient IMD is based on each patient's residential postcode, and the practice score is the mean of individual patient scores using all patients registered at the practice.[8]

Practice registered population

Data on the registered populations for each general practice were obtained from NHS Digital for each quarter from April 2014 to April 2016 (9 datasets); as well as April 2012. These datasets provided the count of patients in each gender by 5-year age-band (with the highest age-band being 95 and over). We aggregated the top three age-bands resulting in a top age-band category of 80+ years.

The April 2012 and April 2016 datasets were used to calculate list sizes weighted for the demographics of the populations and adjusted for deprivation. The reason for weighting for patient demographics is that certain types of patients (older, female and very young) place a higher demand on practices than others. The adjustment for deprivation acknowledges that deprived populations have higher health needs than less deprived populations with a similar demographic profile. To calculate weighted list sizes the practice populations were weighted according to the average time spent consulting with patients in 14 age by gender groups in 2013/14 according to a recent study based on routine patient records from 674 practices.[9] Weighted lists sizes ($\boldsymbol{P_W}$) were then normalised so the total population across the country remained unchanged. These weighted list sizes are taken as a measure of workload on the basis that they represent a measure of the expected time spent consulting. This assumes that, on average, patients in the same demographic group require the same amount of consultation time. Because age and gender do not capture the health status of the population the weighted list sizes

For peer review only - http://bmjopen.bmj.com/site/about/guidelines.xhtml

were then adjusted for deprivation (IMD decile, IMD_i, taking a value between 1 and 10, based on all practices in England) assigning a 10% weighting to a deprived population. The adjusted weighted population will thus be given by

$$P_{AW} = 0.9P_W + 0.1\left(\frac{P_W IMD_i}{\sum P_W IMD_i}\sum P_W\right) \qquad 1$$

This approach is intended to mirror that used in the current resource allocation to CCGs. However, the CCG allocations do not use deprivation, but rather make use of a measure of premature mortality (the <75 standardised mortality ratio, which is the ratio of mortality in under 75 year olds to that expected given the age and sex composition of the CCG population). We chose to use deprivation here as standardised mortality ratios are not published for individual practices.

The adjusted weighted list sizes were divided by the total GP FTE to obtain a measure of workload per GP. Initial inspection of the workload figures showed that the distribution contained some infeasibly large and small values. These may have arisen from errors in either the workforce or practice population data. Unfortunately, there was no clear separation between typical values and those that were infeasible. A pragmatic approach was taken whereby practices in the top and bottom 2.5% of the distribution were excluded from all further analysis. This exclusion took place following the removal of practices with less than 0.5 GP FTE.

The data from April 2014 to April 2016 were used in the prediction of future practice populations along with the subnational population projections described below.

Subnational population projections

We made use of ONS subnational population projections at the level of CCGs (used to inform local planning of healthcare and other public services[10 1110 11]) in the prediction of future practice populations (see below). The subnational ONS projections are demographic, trend-based projections that indicate the 'likely levels of future population' and are currently produced every 2 years; they present projections for every year for the next 25 years from the base year.[12] The underlying data sources that inform the calculations include: national population projections; registration of births and deaths (General Registrar Office); armed forces data (MOD); data extracts from the Patient Register Data System (NHS); student location data (Higher Education Statistics Agency [HESA]); and data on asylum seekers (Home Office). Adjustments were then made to the datasets for factors such as assumed fertility and mortality rates, internal and international migration. However, the projections do not account for local development aims and policies, economic factors, and indeed any international factors that are likely to affect the UK population.[10] We extracted projected populations

For peer review only

for 2021 for the eight CCGs within the scope of the ReGROUP project: NHS Bath and North East Somerset CCG; NHS Kernow CCG; NHS North, East, West Devon CCG; NHS South Devon and Torbay CCG; NHS Bristol CCG; NHS North Somerset CCG; NHS Somerset CCG; NHS South Gloucestershire CCG. Projections are made in 5-year age-bands for each gender. As with practice population data the upper age groups were combined to form an 80+ age-band.

Projecting future workload

Our projections of future practice workloads were based on the number of patients registered at each of the 423 GP practices in South West England, in 5-year age bands, split by gender combined with subnational population projections from the ONS as described above. The approach comprises the following five steps.

1. Assess congruency of ONS predictions with list size
   ONS subnational population projections were compared with GP list size data aggregated to CCG level for 2014, 2015 and 2016. This provided an assessment of the degree to which ONS predications reflect the actual GP list size data in those years. This difference between the two data sources is most likely due to "list inflation", caused by patients that have not been removed from the list following death, dual registrations for patients when moving homes or by a registered patient's failure to complete the national census.[9] Given that the average consultation times used to weight the populations (described above) are based on registered patients, we did not consider it appropriate to resize practice list sizes to reflect the identified difference.
2. Calculate the proportion of CCG population registered at each GP practice
   For each practice, and for each age-band by gender stratum, we identified the number of patients registered with the practice and the expected number of patients within a CCG for nine time-points between April 2014 to April 2016. This allowed us to derive the proportion of the total CCG population by gender/age-interval registered at each practice. If the number of practices in a CCG is declining over time we might expect the proportion of the CCG population to be rising at the remaining practices.
3. Quantify trends in the proportion of the CCG population registered at each general practice
   The data from step 2 were used as the outcome variable in a logistic regression model that included a linear term for time as well as a categorical variable for quarter to quantify trends. A separate regression model was used for each practice by age-band by gender strata.
4. Determine projected count of patients
   We used the resultant regression equation to predict the proportion of CCG patients by practice/gender/age-interval for five years beyond the final data point. Multiplying this

For peer review only - http://bmjopen.bmj.com/site/about/guidelines.xhtml

proportion by the ONS predicted population for the same time point gives a projected count of patients.

5. Project adjusted list size

   The projected populations were used to create a projected adjusted weighted list size using the same algorithm used above for observed populations.

Predicting remaining future workforce

When predicting future workforce (supply) we concentrated on predicting what fraction of the existing workforce will remain available to the practice in 5 years' time. We did this in two principal ways: i) based on the age and gender of GPs at the practice; ii) based on responses to the ReGROUP survey of GP quitting intentions. Predictions are made based on 2012 data and 2016 data (with the survey only being available for the 2016 data).

Approach 1 – Using the age and gender profile of GPs at each practice.

Previous work has identified the probability that GPs of a given single year of age and gender will remain in the workforce 12 months later.[13] By multiplying these probabilities over five consecutive single year age bands we obtain the probability that GPs of a given single year of age and gender will remain in the workforce in 5 years' time. As the routinely available GP census data (p.**Error! Bookmark not defined.**) is only available in five-year age-bands, we take the mean of these 5-year probabilities over the 5-year age-bands used in the GP census data. Unfortunately, the GP census data published at practice level gives data by either age or gender, but not both. Furthermore, data by age is only given in terms of headcount, as is data by gender in 2012 (data by gender is given in terms of headcount and FTE in 2016). Thus we adopted the following procedure to estimate remaining workforce.

1. Using the probabilities described above, identify the probability that each GP in the practice will remain in patient care in 5 years' time based on their age-band assuming they are male.
2. Calculate the mean of these probabilities over all GPs in the practice.
3. Repeat steps 1 and 2 assuming they are female.
4. Take a weighted average of the probabilities obtained in steps 2 and 3 weighted by the FTE of male and female GPs in the practice (in 2012 data headcount by gender is used instead).

For peer review only

The resulting probabilities can then be interpreted as the proportion of GP FTE which is expected, on average, to remain at the practice in 5 years' time.

Approach 2 – Using the ReGROUP survey responses.

An alternative approach used in the forecasting utilised the results of the ReGROUP survey where all GPs in South West England were asked about their future career intentions. For GPs who responded to the survey (67%) we used both stated career intentions, stated FTE (as described above), and information on age and gender. For non-responders we simply used age and gender information (provided within the Performers List). To incorporate the survey responses we made use of odds ratios estimated from a previous study which linked stated quit intentions to working status 5 years later and adjusted for age and gender.[14] Odds ratios for their 5-point scale are mapped to our 4-point scale by ignoring the middle (neutral) option.

1. It proved difficult to map the ReGROUP survey responses to the NHS GP census data (due to inconsistent age, gender and FTE information between the two data sources). Therefore, in this methodology, the GP census data are only used in the estimation of FTE of survey non-responders based on difference between the total GP FTE (GP census data) and the total FTE stated by responders linked to each practice within the Performers List. This was done using the following method. We calculated the difference between the total GP FTE given in the GP census data and the stated total GP FTE of responders to the survey linked through the Performers List to each practice in the study. The assumed FTE of non-responders was this difference divided by the number of non-responders linked to the practice. Where this difference was greater than the number of FTEs, the non-responders were assigned an FTE of 1. Where this difference was negative, non-responders were assigned an FTE of 0.

2. We then calculated probabilities of remaining in patient care for the forthcoming 5 years. For the survey non-responders, we assigned a probability of remaining in patient care using the same method as in approach 1 but based on the individual GP's gender and current year of age taken from the Performers List (rather than the GP census). For responders, we similarly assigned a probability of remaining in patient care based on the individual GP's age and gender and then adjusted that probability using the following odds ratios (Calculated from Hann et al. [14] but changing the baseline to the neutral category) "Very likely" 1.94, "Likely" 1.3, "Unlikely" 0.70, and "Very unlikely" 0.43.

3. For each practice, we then took the weighted average of the probabilities obtained in step 2 (over GPs associated with a practice, weighted according to their FTE.

The resulting probabilities can then be interpreted as the proportion of GP FTE that would be expected, on average, to remain at the practice in 5 years' time.

References

1. NHS England. GP Patient Survey: NHS England; [Available from: https://gp-patient.co.uk/surveys-and-reports accessed 05/03/2018.
2. Paddison C, Elliott M, Parker R, et al. Should measures of patient experience in primary care be adjusted for case mix? Evidence from the English General Practice Patient Survey. *BMJ Qual Saf* 2012
3. Abel GA, Saunders CL, Lyratzopoulos G. Cancer patient experience, hospital performance and case mix: evidence from England. *Future Oncol* 2014;10(9):1589-98. doi: 10.2217/fon.13.266
4. NHS Digital. General and personal medical services, England 2002-2012 as at 30 September 2012 [Available from: http://content.digital.nhs.uk/searchcatalogue?productid=10382&topics=1%2fWorkforce%2fStaff+numbers&kwd=GP+staff&geo=England&sort=Relevance&size=10&page=2#top accessed 31/07/2017 2017.
5. NHS Digital. General and personal medical services, England 2003-2013 as at 30 September 2013 [
6. NHS Digital. General and personal medical services, England September 2015-March 2016, Provisional Experimental statistics 2016 [Available from: http://content.digital.nhs.uk/catalogue/PUB21772 accessed 31/07/2017 2017.
7. Fletcher E, Abel GA, Anderson R, et al. Quitting patient care and career break intentions among general practitioners in South West England: findings of a census survey of general practitioners. *BMJ Open* 2017;7(4):11. doi: 10.1136/bmjopen-2017-015853
8. Public Health England. National General Practice Profile 2013 [Available from: http://fingertips.phe.org.uk/profile/general-practice accessed August 2104 2014.
9. Hobbs FR, Bankhead C, Mukhtar T, et al. Clinical workload in UK primary care: a retrospective analysis of 100 million consultations in England, 2007–14. *The Lancet* 2016;387(10035):2323-30.
10. Office for National Statistics (ONS). Subnational population projections for England: 2014-based projections 2014 [Available from: https://www.ons.gov.uk/peoplepopulationandcommunity/populationandmigration/populationprojections/bulletins/subnationalpopulationprojectionsforengland/2014basedprojections accessed 31/07/2014 2017.
11. Office for National Statistics. Subnational population projections for England: 2014-based projections 2014 [Available from: https://www.ons.gov.uk/peoplepopulationandcommunity/populationandmigration/populationprojections/bulletins/subnationalpopulationprojectionsforengland/2014basedprojections accessed 31/07/2014 2017.
12. Office for National Statistics (ONS). Quality and Methodology Information - Subnational Population Projection. 2016

For peer review only

13. Centre for Workforce Intelligence. In-depth review of the general practitioner workforce: final report. London, 2014.
14. Hann M, Reeves D, Sibbald B. Relationships between job satisfaction, intentions to leave family practice and actually leaving among family physicians in England. *European journal of public health* 2011;21(4):499-503. doi: 10.1093/eurpub/ckq005 [published Online First: 2010/02/10]

For peer review only

Appendix 2a – Data Flow Main development model

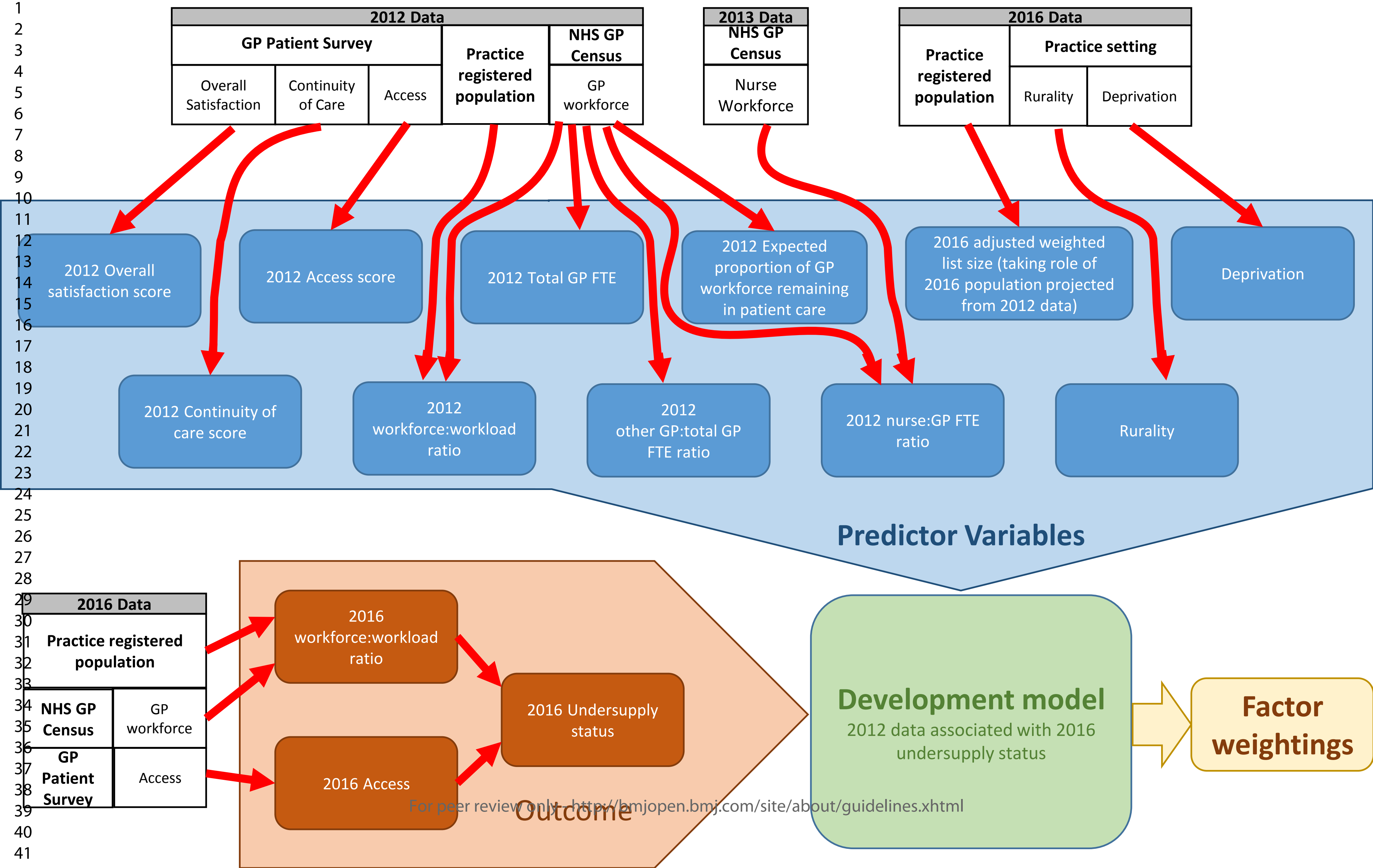

For peer review only - http://bmjopen.bmj.com/site/about/guidelines.xhtml

Appendix 2b – Data Flow Main prediction model

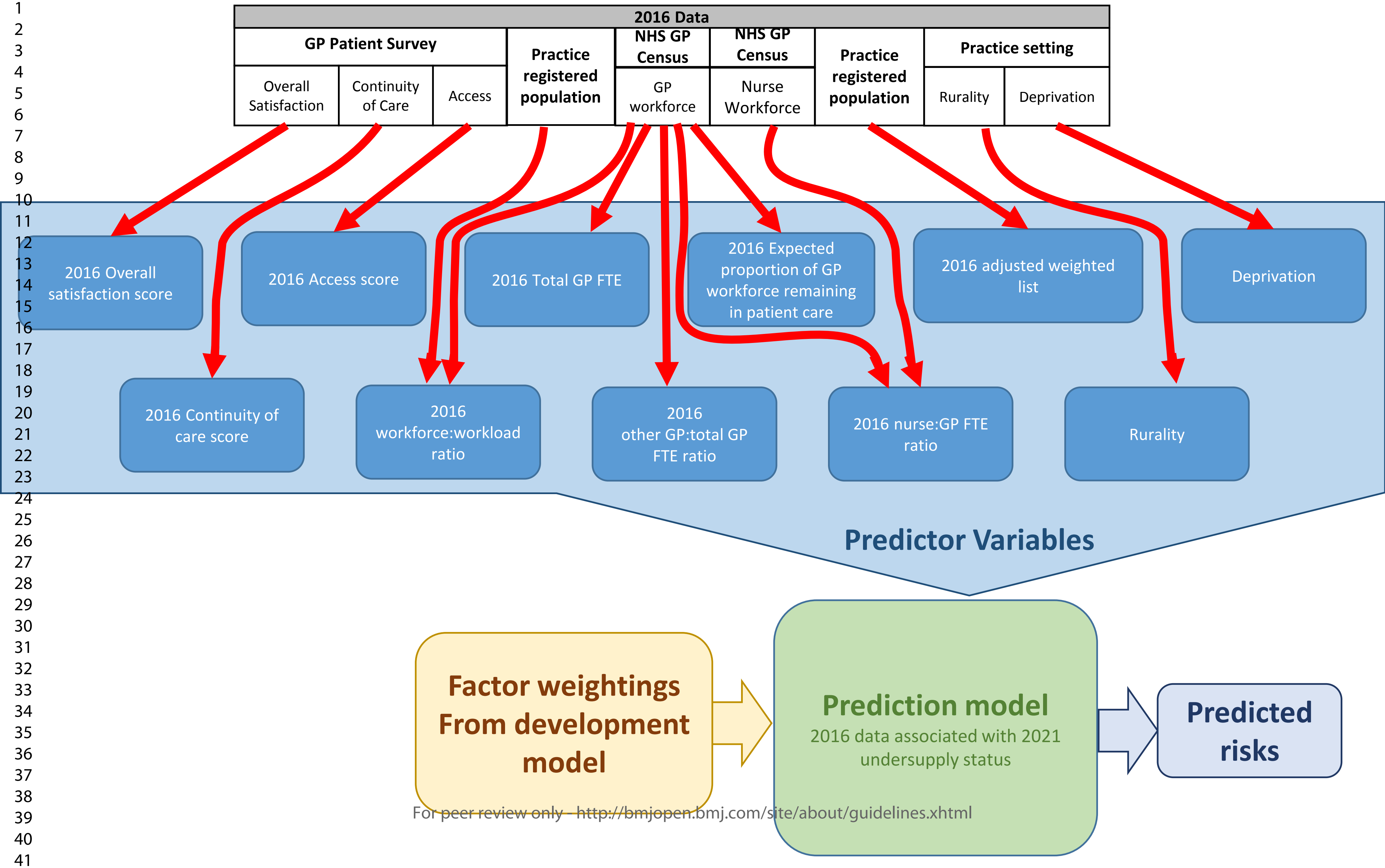

Appendix 2c – Data Flow Simpler development model

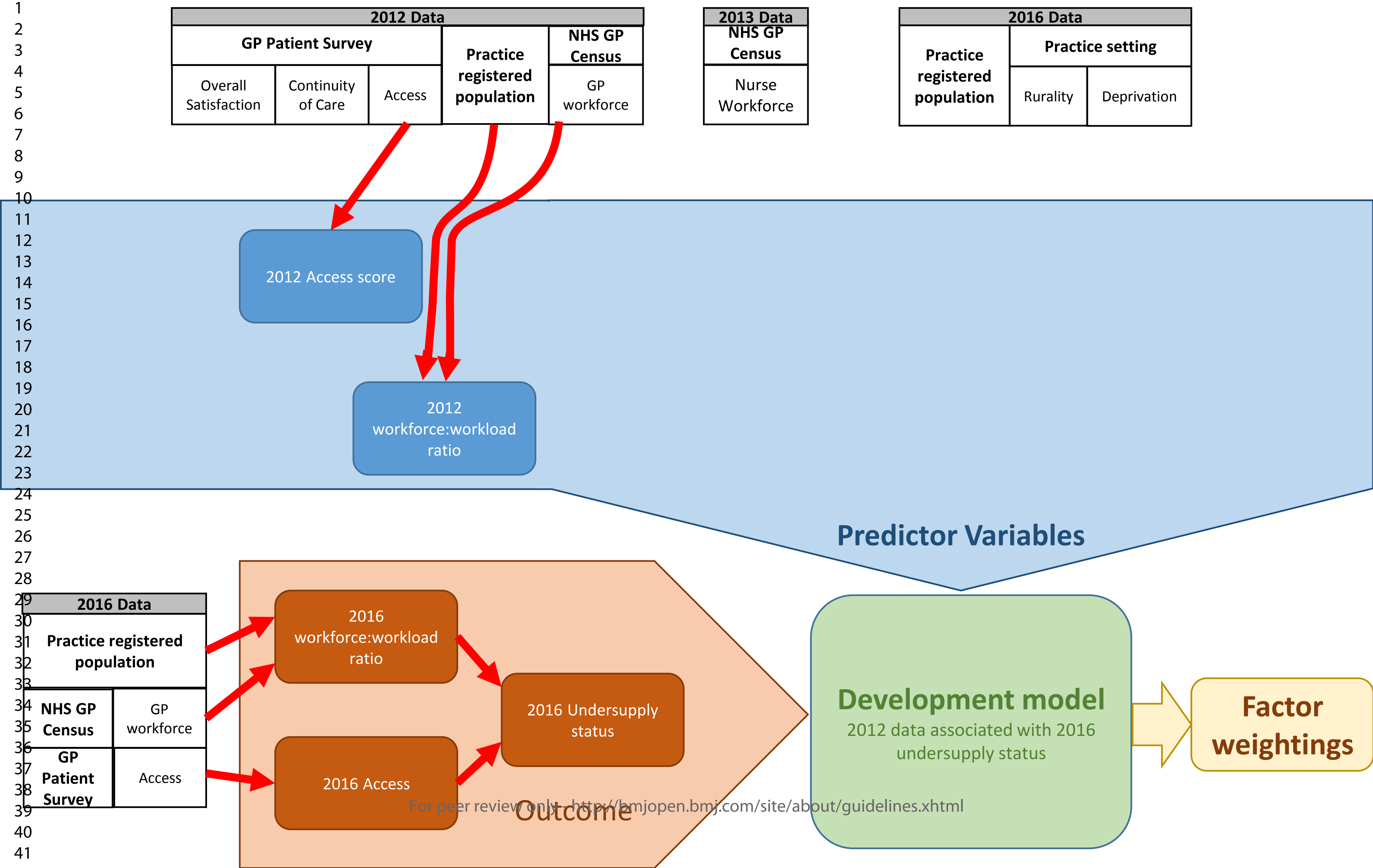

For peer review only - http://bmjopen.bmj.com/site/about/guidelines.xhtml

Appendix 2d – Data Flow Simpler prediction model

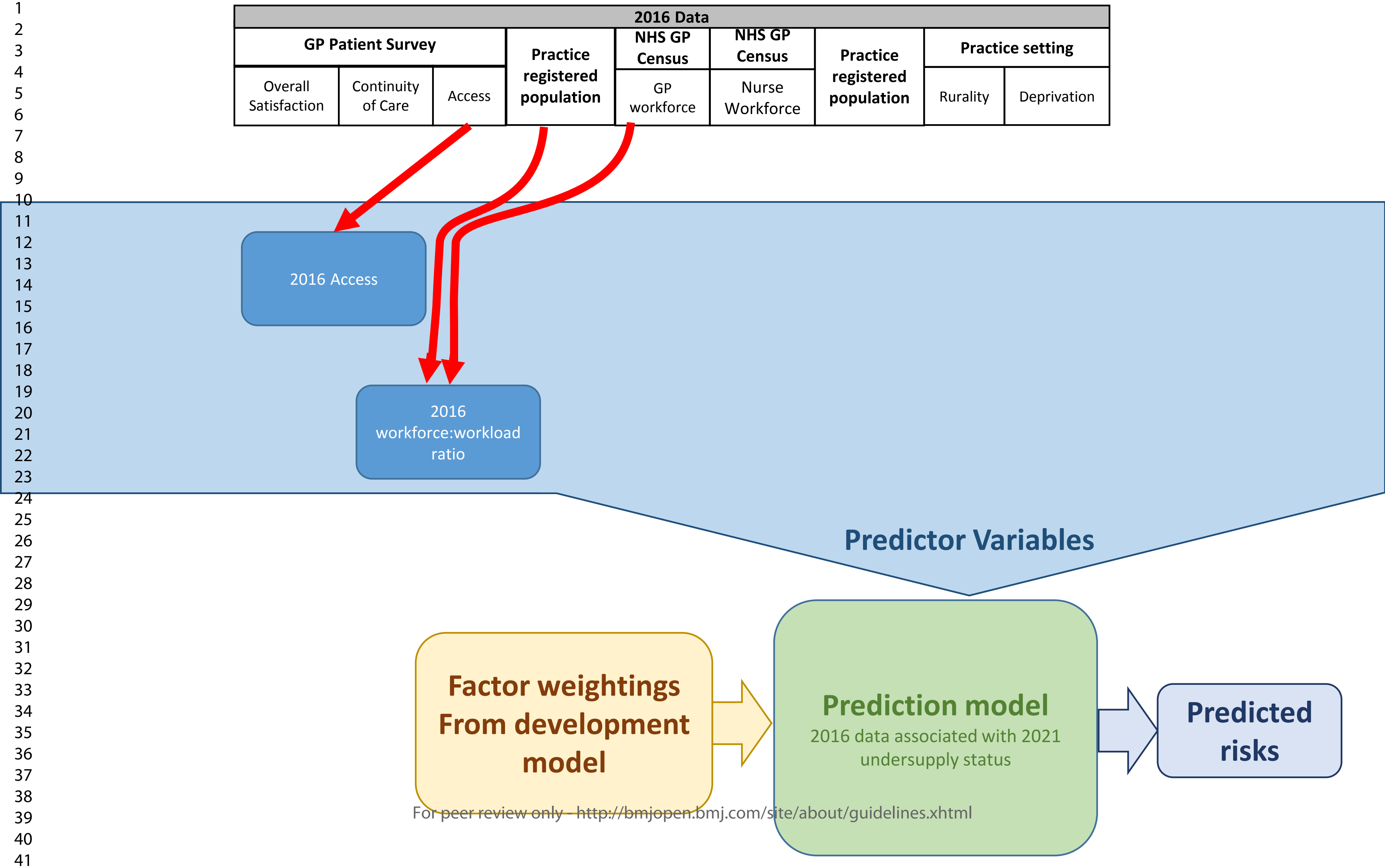

For peer review only - http://bmjopen.bmj.com/site/about/guidelines.xhtml

Appendix 3 – Calibration curve

In order to assess the calibration of the model we used predicted probabilities of being in undersupply from the development model (i.e. 2012 covariates and 2016 outcome) and split the practices into 10 groups according to deciles of this predicted probability. We then calculated the mean predicted probability in each group as well as the percentage of practices in undersupply in 2016. The relationship between these two quantities is shown in the figure below.

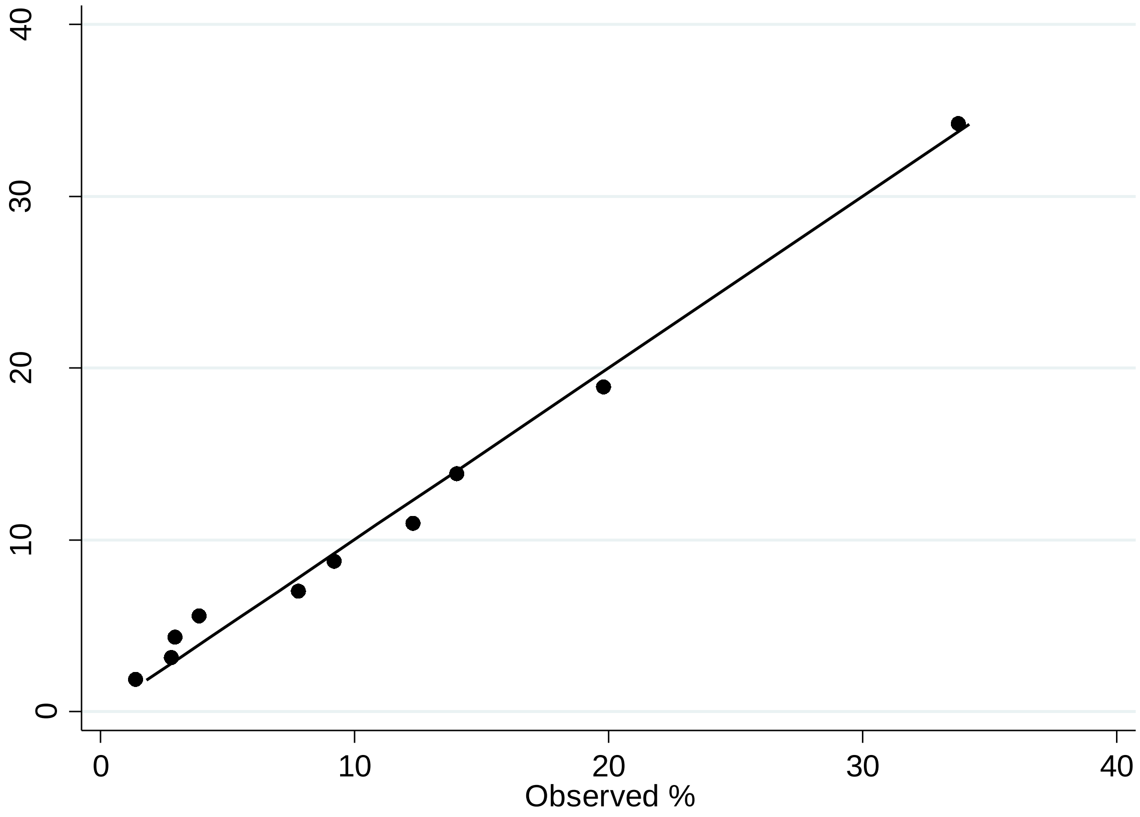

TRIPOD

TRIPOD Checklist: Prediction Model Development

Section/Topic	Item	Checklist Item	Page
**Title and abstract**			
Title	1	Identify the study as developing and/or validating a multivariable prediction model, the target population, and the outcome to be predicted.	1
Abstract	2	Provide a summary of objectives, study design, setting, participants, sample size, predictors, outcome, statistical analysis, results, and conclusions.	2
**Introduction**			
Background and objectives	3a	Explain the medical context (including whether diagnostic or prognostic) and rationale for developing or validating the multivariable prediction model, including references to existing models.	4
	3b	Specify the objectives, including whether the study describes the development or validation of the model or both.	4
**Methods**			
Source of data	4a	Describe the study design or source of data (e.g., randomized trial, cohort, or registry data), separately for the development and validation data sets, if applicable.	4-6
	4b	Specify the key study dates, including start of accrual; end of accrual; and, if applicable, end of follow-up.	4-9
Participants	5a	Specify key elements of the study setting (e.g., primary care, secondary care, general population) including number and location of centres.	4-6
	5b	Describe eligibility criteria for participants.	7
	5c	Give details of treatments received, if relevant.	N/A
Outcome	6a	Clearly define the outcome that is predicted by the prediction model, including how and when assessed.	7
	6b	Report any actions to blind assessment of the outcome to be predicted.	N/A
Predictors	7a	Clearly define all predictors used in developing or validating the multivariable prediction model, including how and when they were measured.	4-9
	7b	Report any actions to blind assessment of predictors for the outcome and other predictors.	N/A
Sample size	8	Explain how the study size was arrived at.	7
Missing data	9	Describe how missing data were handled (e.g., complete-case analysis, single imputation, multiple imputation) with details of any imputation method.	7
Statistical analysis methods	10a	Describe how predictors were handled in the analyses.	7-9
	10b	Specify type of model, all model-building procedures (including any predictor selection), and method for internal validation.	9
	10d	Specify all measures used to assess model performance and, if relevant, to compare multiple models.	8-9
Risk groups	11	Provide details on how risk groups were created, if done.	8
**Results**			
Participants	13a	Describe the flow of participants through the study, including the number of participants with and without the outcome and, if applicable, a summary of the follow-up time. A diagram may be helpful.	9
	13b	Describe the characteristics of the participants (basic demographics, clinical features, available predictors), including the number of participants with missing data for predictors and outcome.	9
Model development	14a	Specify the number of participants and outcome events in each analysis.	9
	14b	If done, report the unadjusted association between each candidate predictor and outcome.	N/A
Model specification	15a	Present the full prediction model to allow predictions for individuals (i.e., all regression coefficients, and model intercept or baseline survival at a given time point).	18
	15b	Explain how to the use the prediction model.	N/A
Model performance	16	Report performance measures (with CIs) for the prediction model.	11
**Discussion**			
Limitations	18	Discuss any limitations of the study (such as nonrepresentative sample, few events per predictor, missing data).	13
Interpretation	19b	Give an overall interpretation of the results, considering objectives, limitations, and results from similar studies, and other relevant evidence.	12-13
Implications	20	Discuss the potential clinical use of the model and implications for future research.	14
**Other information**			
Supplementary information	21	Provide information about the availability of supplementary resources, such as study protocol, Web calculator, and data sets.	N/A
Funding	22	Give the source of funding and the role of the funders for the present study.	3

We recommend using the TRIPOD Checklist in conjunction with the TRIPOD Explanation and Elaboration document.

For peer review only - http://bmjopen.bmj.com/site/about/guidelines.xhtml
